# Chronic Hypoxia Disrupts Spermatogenesis Through ASXL2–EZH2–Mediated Microtubule Destabilization

**DOI:** 10.1002/advs.202501266

**Published:** 2026-03-04

**Authors:** Jun Yin, Mengjie Zhang, Wenying Liu, Wenlong Shen, Debao Li, Hongming Miao, Fang Deng, Gang Zhang, Yi Tian, Yi Zhang, Zhihu Zhao, Bing Ni

**Affiliations:** ^1^ Department of Pathophysiology Army Medical University Chongqing China; ^2^ Key Laboratory of Extreme Environmental Medicine Ministry of Education of China Chongqing China; ^3^ Key Laboratory of High Altitude Medicine PLA Chongqing China; ^4^ Department of Dermatology Southwest Hospital Army Medical University Chongqing China; ^5^ National Key Laboratory of Advanced Biotechnology Academy of Military Medical Sciences Beijing Beijing China; ^6^ Chongqing International Institute for Immunology Chongqing China; ^7^ Department of Immunology Army Medical University Chongqing China

**Keywords:** ASXL2, CEP162, chronic hypoxia, EZH2, H3K27me3, PRC2, spermatogenesis

## Abstract

Chronic hypoxia disrupts spermatogenesis by blocking the round‐to‐elongated spermatid transition, a process governed by the additional sex combs‐like 2 (ASXL2)–enhancer of zeste homolog 2 (EZH2) axis. Hypoxia downregulates ASXL2 expression, which reduces EZH2 binding to the 3482–3511 bp region of the CEP162 promoter. This impairment decreases H3K27me3 modification while increasing CEP162 transcription. Overexpressed CEP162 competes with TUBA3A for TUBB3 binding. This competition depletes ciliary TUBB3 levels, destabilizing axonemal microtubules. These structural defects are correlated with sperm malformations and functional deficiencies. In infertile men, diminished ASXL2 and EZH2 expression highlights the therapeutic potential of targeting this axis for hypoxia‐related spermatogenic disorders.

## Graphical abstract

This study reveals the mechanism by which chronic hypoxia impairs spermatogenesis via the ASXL2–EZH2 axis, hindering the transition of spermatids from round to elongated forms. Key findings reveal that under hypoxic conditions, downregulated ASXL2 expression reduces EZH2 binding to the CEP162 promoter, leading to decreased H3K27me3 modification and increased CEP162 transcription. This mechanism results in microtubule instability, affecting sperm morphology and function. Clinically, reduced ASXL2 and EZH2 expression in infertile men highlights the therapeutic potential of the ASXL2–EZH2 axis in hypoxia‐related spermatogenic disorders.

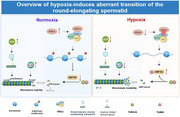

## 1 Introduction

Environmental hypoxia—a state of reduced oxygen availability caused by high‐altitude exposure, urbanization‐related air pollution, and pathological/iatrogenic factors (e.g., chronic obstructive pulmonary disease, radiation‐induced fibrosis)—pose a critical threat to male fertility by disrupting spermatogenesis and germ cell development [1]. Hypoxia manifests in two distinct forms in mammals: environmental hypoxia (low inhaled oxygen partial pressure due to high‐altitude or polluted environments, among other factors) and pathological hypoxia (impaired testicular oxygen delivery/utilization due to varicocele, chronic lung disease, or sickle cell disease) [2]. Both forms alter testicular transcriptomes and reduce sperm count, motility, and morphology, with the World Health Organization estimating that more than 300 million individuals experience chronic hypoxia caused by air pollution and sedentary lifestyles globally [3]. The clinical urgency of hypoxia extends beyond high‐altitude populations to cancer survivors (gonadotoxic therapy‐induced vascular damage), aging males (age‐related testicular hypoperfusion), and individuals with chronic inflammatory disorders (e.g., Crohn's disease), where oxidative testicular microenvironments exacerbate reproductive dysfunction [4]. Mechanistically, hypoxia disrupts epigenetic regulation (e.g., histone methylation/acetylation dynamics) and metabolic homeostasis in germ cells, destabilizing microtubule networks and impairing spermiogenesis [5]. However, the molecular pathways linking oxygen sensing to these dual pathogenic mechanisms remain poorly defined, limiting therapeutic strategies for hypoxia‐associated infertility.

In addition to understanding the broader context of environmental impacts, understanding the cellular processes underlying sperm development is critical for investigating therapeutic strategies for hypoxia‐associated infertility. Spermiogenesis, the intricate process that transforms haploid spermatids to spermatozoa, hinges on the critical transition from round to elongating spermatids. This pivotal step is marked by substantial alterations in the microtubule cytoskeleton. The dynamics of microtubule assembly within the ciliary cap are crucial for axoneme elongation [6], with microtubules extending toward the nascent sperm tail. Any disruption to these microtubules can halt proper elongation, leading to the persistence of condensed, round spermatid nuclei. This phenomenon is exemplified in mice deficient in Smg6, where spermatogenesis is arrested at the round spermatid stage because of the protein's role in facilitating the transition to elongating spermatids [7]. Concurrently, the manchette—a microtubule‐based structure—emerges during this transition and can be visualized using β‐tubulin antibodies [8]. The sensitivity of Drosophila spermatid flagella to nocodazole, a microtubule‐destabilizing drug, further underscores the importance of microtubule stability. Single‐cell RNA sequencing studies have elucidated the dynamic processes occurring during this transition in human spermatogenesis [9]. The role of the kinesin‐13 Klp10A, a microtubule‐depolymerizing enzyme, in the transition from round spermatids to spermatozoa underscores the importance of microtubule destabilization [6].

The transition zone, a specialized compartment at the base of cilia, plays a critical role in ciliary assembly and function, serving as a gatekeeper for the entry and exit of proteins into and out of the cilium [10]. Studies have shown that the transition zone is involved in axoneme formation in Drosophila male germ cells [11]. Additionally, ESCRT III components and VPS4 are localized to the ciliary transition zone, where they impact centrosome duplication and spindle pole stability. The identification of cell‐specific alpha‐tubulin TBA‐6 and periciliary IFT cargo RAB‐28 as regulators of noncanonical transition zones suggests the occurrence of unique structural events and mechanisms that govern ciliary architecture [12]. This intricate regulation of the transition zone is not only crucial for ciliary function but also points to the complexity of proteins that orchestrate these processes.

Another molecular player that governs the transition zone, CEP162, a 162 kDa centrosomal protein in *Homo sapiens*, has emerged as an essential axoneme recognition protein that facilitates the assembly of the transition zone in primary cilia by specifically recognizing and binding to axonemal microtubules [13]. A lack of CEP162 results in the delayed formation of dysmorphic cilia, particularly those impacting transition zone assembly [14]. Moreover, studies have revealed that overexpression of CEP162 results in its ectopic distribution within the distal segments of cilia in retinal pigment epithelial cells. This aberrant localization leads to swelling and ectopic accumulation of transition zone proteins, further emphasizing its essential role in ciliary function [13]. Considering the ability of CEP162 to promote the ectopic assembly of the transition zone, we hypothesize that its overexpression can alter the distribution of tubulin dimers within cilia by modulating the function of the transition zone.

ASXL2, a member of the ASXL family, has been demonstrated to interact with polycomb repressive complex 2 (PRC2), as evidenced by its colocalization with target promoters in the adult heart, suggesting its role in transcriptional regulation. This interaction is supported by the role of another family member, ASXL1, in transcriptional repression through its association with PRC2, which suggests a conserved function for ASXL2 in this process [15]. The in vivo interaction of ASXL2 with PRC2 potentially influences the transcription of EZH2, a key component of PRC2. Although previous studies have explored the impact of hypoxia on spermatogenesis and the interactions between EZH2 and hypoxia effectors [16], to date, studies have investigated only the collaborative regulation of coding gene transcription by ASXL2 and PRC2 in nonspermatogonial cells [17]. No research has directly addressed the interactions between ASXL2 and the three subunits of PRC2 or their collective role in regulating the transcription of spermatogonial coding genes.

In this study, we utilized single‐cell RNA sequencing (scRNA‐seq) and experimental evidence to elucidate the underlying mechanisms leading to a diminished mature sperm count and increased sperm deformity rates under environmental hypoxia in rats. Our results suggest that the suppressive transcriptional complex comprising ASXL2 and EZH2 may be critical in this process, because of its role in targeting genes essential for spermatid proliferation and flagellation, such as CEP162. This hypothesis aligns with clinical observations in patients with teratozoospermia, where reduced expression of polycomb genes is associated with sperm abnormalities.

## 2 Results

### 2.1 Chronic Hypoxia Markedly Inhibits the Transition of Round–Elongating Spermatids Through Microtubule Destabilization

Hypoxia affects spermatogenesis; however, the underlying mechanisms remain largely uncharacterized [18]. In this study, we first investigated the effects of severe chronic hypoxia on rat spermatogenesis by housing the rats in a hypobaric oxygen chamber with an 11.7% oxygen concentration (simulating an altitude of 5800 m) while keeping age‐ and sex‐matched control rats housed under a normal oxygen concentration (21%) for 10 weeks. Compared with those of the control rats, the walls of the spermatogenic tubules of the rats exposed to hypoxia were thinner, the polarity arrangement of the spermatogenic cells was disrupted, the number of secondary spermatocytes was reduced, and spermatids were infrequently observed in the cavity of the seminiferous tubules (Supplementary Figure S1A). In addition, we detected aberrantly decreased plasma levels of male sex hormones, including testosterone (T), androgen binding protein (ABP), luteinizing hormone (LH) and follicle‐stimulating hormone (FSH), in the hypoxia group, although the plasma gonadotropin‐releasing hormone (GnRH) level did not obviously differ between the groups (Supplementary Figure S1C). We further examined the penetration, viability, motility, motion parameters and number of spermatids in the cauda epididymis. While viability did not obviously differ between the two groups, sperm penetration, count and motility decreased considerably after exposure to hypoxia (11.7% oxygen concentration) for 10 weeks (Supplementary Figure S1D,E). We also observed an aberrantly increased rate of sperm deformation, including defects in the sperm tail, under hypoxic conditions. Atomic force microscopy (AFM) analysis of ciliary surface force revealed diminished rigidity and elasticity after exposure to hypoxia, indicating structural abnormalities in the ciliary cytoskeleton (Supplementary Figure S1B, Figure 1a A,B). Additionally, we utilized transmission electron microscopy to examine rat sperm cilia. Under hypoxic conditions, the centrosomes of axonemal microtubules tended to develop structural irregularities, including missing outer doublet microtubules (DMTs; indicated by black arrows in Figure 1a C) and disorganized microtubule arrangement. These defects were quantitatively correlated with reduced microtubule stability (Figure 1a C, right panel). These findings indicate that hypoxia results in a reduction in the stability of the microtubules of rat sperm cilia, potentially due to impaired microtubule assembly or increased depolymerization.

FIGURE 1(A–D) Hypoxia‐induced microtubule destabilization in spermatid axonemes. The rats were housed under normoxic (21% O_2_ concentration) or hypoxic (11.7% O_2_ concentration) conditions for 10 weeks, the testes were harvested, and the sperm in the epididymis were collected for serial examinations. (A) Papanicolaou staining was used to examine the morphology of the flagella in each group. The representative photos show the morphological changes in the sperm flagella in two fields of view for the normoxic and hypoxic groups, with arrows indicating sperm with a normal tail, head deformity, and tail deformity. Quantitative analysis of head and tail abnormalities between normoxic and hypoxic conditions (n = 12). (B) Representative adhesion force curves collected with the force mapping mode for cilia samples measured with silica tips. The force‐mapping mode enables statistical analyses of cilia adhesion on the basis of the random measurement of spots on the cilial surfaces with a scanning range of 40–60 µm. The x‐axis, ZSnsr (Z sensor), represents the displacement between the sample surface and the resting position of the cantilever. The red line represents the “approach curve,” whereas the blue line denotes the “retract curve,” illustrating the interaction between the probe and the sample surface during the approach and retraction phases of AFM measurement. The bar graph provided illustrates the data for the sample modulus and reduced modulus on the surface of the cilium (n = 12). (C) Cross‐sectional transmission electron microscopy (TEM) analysis of spermatozoa from rats exposed to hypoxia. Missing DMTs are designated by black arrows. Quantitative analysis of centrosome irregularity between normoxic and hypoxic conditions (n = 12). (D) Microtubule regrowth assay after nocodazole washout in G1 and G2 cells under 1% O_2_ exposure. The representative figures depict the ratio of GFP‐tagged α‐tubulin fluorescence intensity and the percentage of cells expressing γ‐tubulin (n = 12). The data are presented as the means ± SDs; ***p < 0.001, ****p < 0.0001; 2‐tailed, unpaired Student's *t*‐test (A–D). (E–I) Disruption of the round‐to‐elongating spermatid transition by hypoxia through inhibition of microtubule assembly. (E) After a 48‐h incubation under 1% O_2_, the time course of fluorescence recovery in the bleached area was examined. The graph shows the percentage of fluorescence recovery over time following photobleaching (n = 6). (F) Following lentivirus‐mediated overexpression of TUBA1A in G2 cells, FRAP analysis was conducted to compare the microtubule polymerization and depolymerization rates in G2 cells. (G) Cartoon diagram illustrating the digestion of testicular tissue to extract sperm cells, followed by sorting through flow cytometry to differentiate between round and elongated spermatids. Panel G was created using FigDraw (https://figdraw.com). (H) Overview of major cell types inferred from 10× Genomics single‐cell RNA‐seq data of rat testes. UMAP plots of all cells and cells assigned to known putative cell types. The identified spermatogonia, spermatocytes, round spermatids and elongating spermatids followed the sequential developmental trajectory of spermatogenesis. The observed “Y”‐shaped pattern at the terminal stage of spermatid differentiation is an artifact of nonlinear dimensionality reduction rather than a true biological branching event (see Supplementary Figure S1F,G). (I) Distribution profiles of cell attributes. chrM%, chrX%, and chrY% are the percentages of UMIs mapped to mitochondrial genes, chromosomal X genes and chromosomal Y genes, respectively. The Gini index indicates the gene expression inequality for expressed genes. The diversity values indicate the dissimilarities among cells within the same cell type, measured as the 1‐mean Pearson correlation coefficient.

**Figure:**
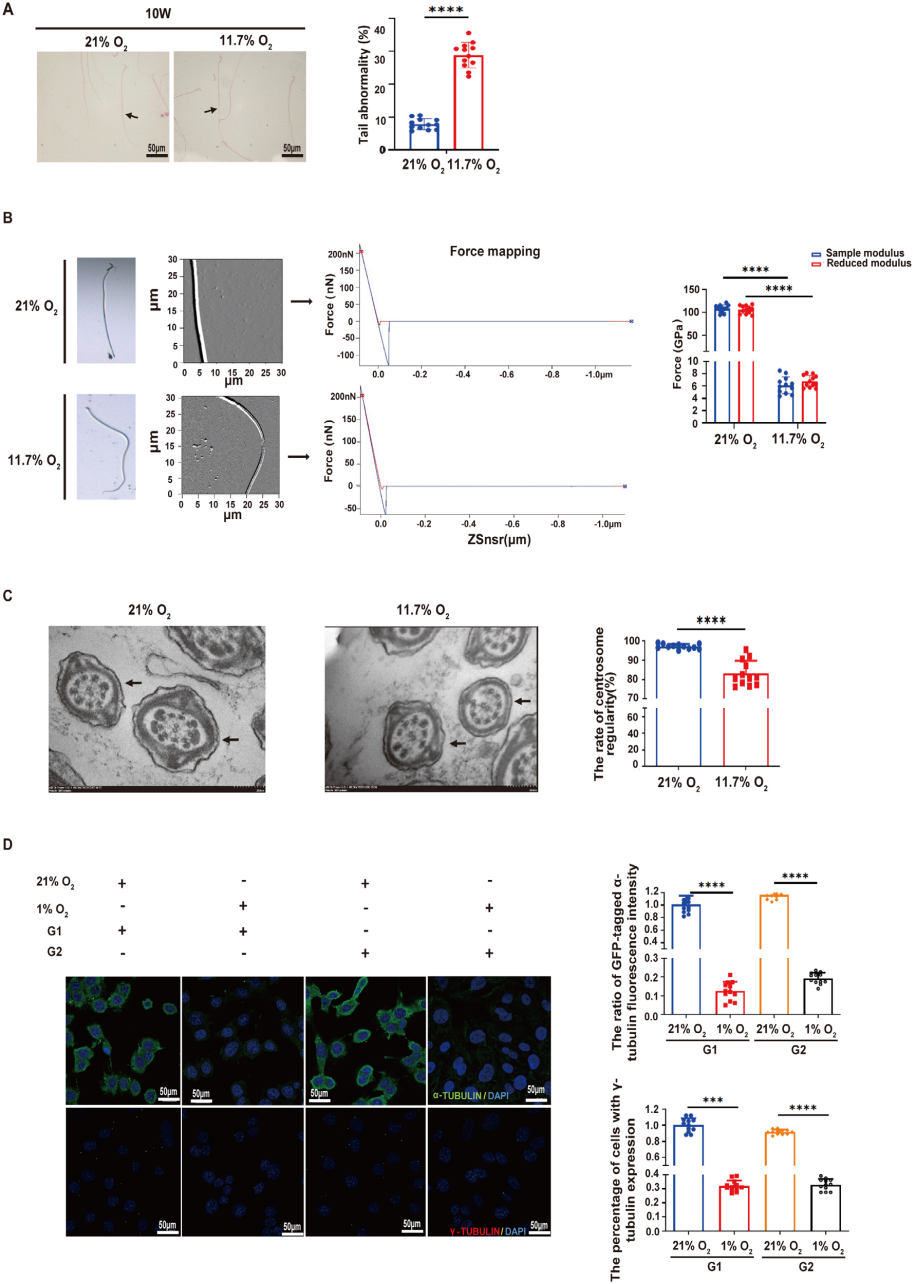

**Figure:**
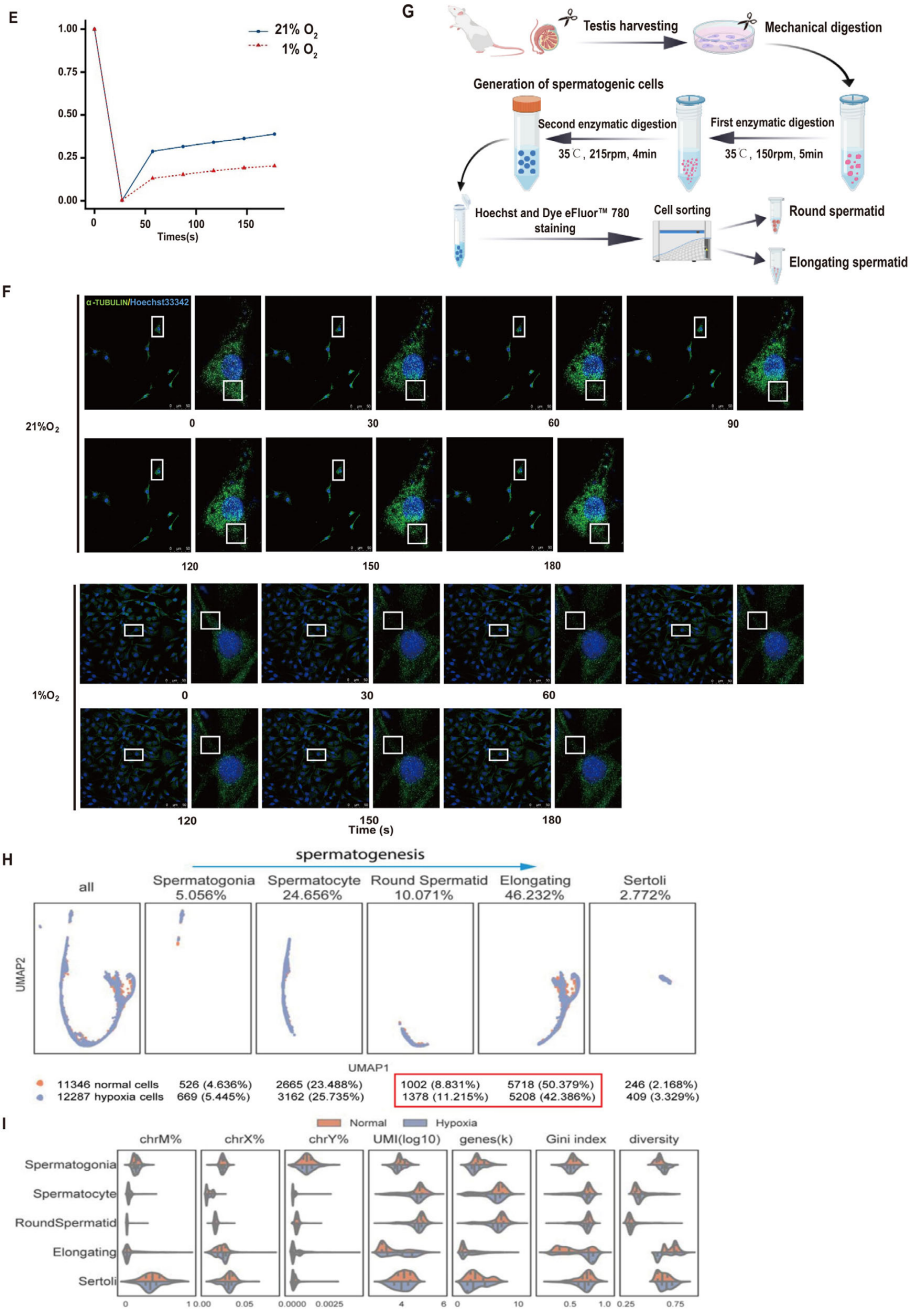

Through microtubule regrowth and fluorescence recovery after photobleaching (FRAP) experiments, we further observed that hypoxia exposure led to a reduction in microtubule assembly within the sperm flagella (Figure 1a D, Figure 1b E,F). Previous reports have suggested that diminished microtubule stability may be correlated with imbalance in ATP, GTP, and calcium ions levels. Our research suggests that hypoxia may trigger changes in the levels of these cellular factors within spermatogenic cells or sperm, thereby causing microtubule destabilization. Using liquid chromatography, we found that hypoxic conditions led to a reduction in ATP, GTP and calcium ion levels, which can contribute to microtubule instability. Previous reports have indicated that hypoxia can influence the distribution of anterograde intraflagellar transport motor proteins and tubulin dimers through ATP [19], and we similarly observed that hypoxia restricts the distribution of KIF3A, KIF3B, KIF5B, KIF21A, KLC4, TUBA1B, and TUBB3 within cilia (Supplementary Figure S2A–F). These findings suggest that hypoxia impedes spermatogenesis by promoting the destabilization of ciliary microtubules in sperm. Previous investigations into the effects of hypoxia on spermatogenesis have focused predominantly on its impact at specific cellular stages. However, the influence of hypoxia may extend beyond direct effects on sperm. Despite the importance of understanding these processes, a systematic exploration into the comprehensive effects and underlying mechanisms of hypoxia throughout the spermatogenic process is notably absent from the literature.

We next further prepared mixed cells from rat testes via a 10× Genomics scRNA‐seq protocol and sequenced 3 libraries of normal rat testes and 3 libraries of hypoxia‐treated rat testes, resulting in a total of approximately 26 000 cells and approximately 2.4 billion reads (Supplementary Table S1, Methods). We observed a continuous distribution pattern for a majority of the cells (Supplementary Figure S1D), which is consistent with the distribution patterns from principal component analysis (PCA) and t‐distributed stochastic neighbor embedding (tSNE) for both human and mouse testis single‐cell analyses in the literature [20]. Moreover, we attempted to assign a putative cell type to each cell on the basis of the marker genes identified from a study of approximately 35 000 single mouse testis cells [21]. The cell types were assigned on the basis of the highest enrichment score (ES) with a stringent false discovery rate threshold (FDR <0.001). Cells meeting these criteria were classified into definitive categories (e.g., spermatogonia, and spermatocytes), and their proportions were quantified (Supplementary Figure S1F,J). Additionally, examination of the percentage of cells in each stage revealed that hypoxia led to fewer elongating spermatids (normal: 50.397%; hypoxia: 42.386%) but more round spermatids (normal: 8.831%; hypoxia: 11.215%), suggesting that the transition from round to elongating spermatids may be affected (Supplementary Figure S1K–P, Figure 1b G–I). In summary, our findings highlight a considerable obstruction in the maturation phase of elongating spermatids, with a significant impact on the stability of ciliary microtubules. This instability could be the underlying cause for the marked decrease in the number of mature sperm observed in rats that have been chronically exposed to hypoxic conditions.

### 2.2 Key Potential Regulatory Factors in the Round–Elongating Spermatid Transition After Chronic Hypoxia

**FIGURE 2:**
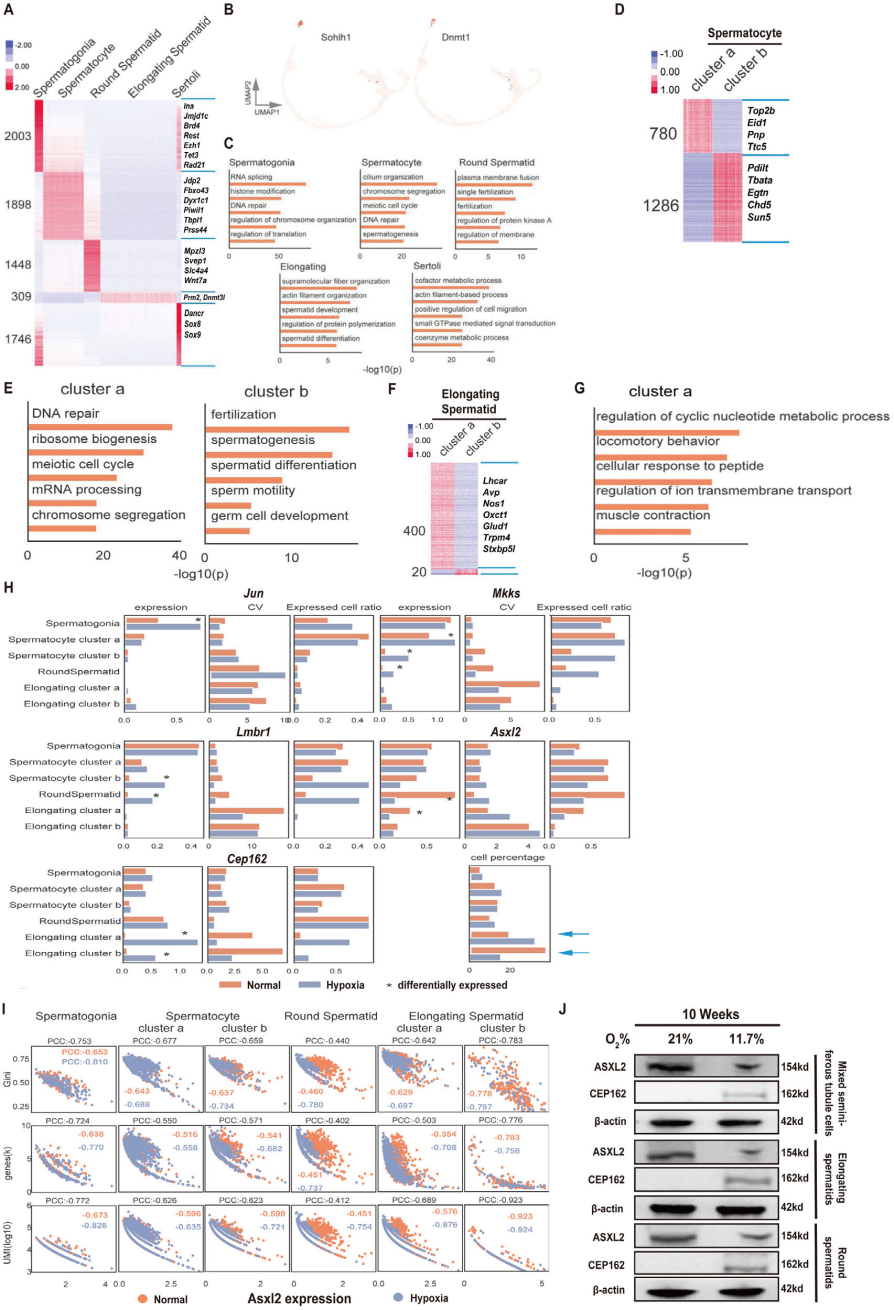
Differentially expressed genes between control and hypoxia‐treated rat testes. (A) Heatmap of putative cell type‐specific gene expression. (B) Two examples of spermatogonia‐specific gene expression patterns in the UMAP plot. (C) Top 5 enriched GO terms of specifically expressed genes for each cell type. See also Supplementary Figure S1, Supplementary Figure S2, and Supplementary Figure S3. (D) Heatmap of putative subtype‐specific gene expression. (E) Top 5 enriched GO terms among genes specifically expressed in each subtype. (F) Heatmap of putative subtype‐specific gene expression. (G) Top 5 enriched GO terms for genes specifically expressed in Cluster A. No enriched terms were found for Cluster B. (H) Examples of differentially expressed genes. CV stands for the coefficient of variation. The blue arrows indicate the stages with large differences in cell percentages between the normal and hypoxia treatment groups. * indicates the stages at which the gene was identified as a DEG. (I) Correlations between ASXL2 and the Gini index (first row), number of expressed genes (thousands) and number of UMIs (log10 transformed) in ASXL2‐expressing cells of different cell types. PCC stands for the Pearson correlation coefficient. (J) Western blot analysis of the protein expression of ASXL2 and CEP162 in lysates of mixed seminiferous tubule cells, elongating spermatids, and round spermatids from rats housed under normal conditions or hypoxic conditions for 10 weeks (n = 3). See also Supplementary Figure S3.

We further identified the marker genes for each putative cell type/subtype (Figure 2A,D,F), and the top enriched Gene Ontology (GO) terms of these marker genes were consistent with the known properties of each cell type/subtype (Figure 2C,E,G). The cell type annotation was rigorously validated using a panel of 22 well‐established lineage‐specific markers (Supplementary Table S2), including those of spermatogonia (Ina, Jmjd1c, Brd4, Rest, Ezh1, Tet3, and Rad21), spermatocytes (Jdp2, Fbxo43, Dyx1c1, Piwil1, Tbpl1, and Prss44), round spermatids (Mpzl3, Svep1, Slc4a4, and Wnt7a), elongating spermatids (Prm2 and Dnmt3l), and Sertoli cells (Dancr, Sox8, and Sox9). As expected, the essential spermatogenesis and oogenesis‐specific basic helix‐loop‐helix gene *Sohlh1* [22] and the important DNA methylation‐catalyzed gene *Dnmt1* [23] were specifically expressed in spermatogonia (Figure 2B). The specific expression of genes encoding histone modification enzymes, such as *Jmjd1c* and *Brd4*, and the chromosome structure and organization gene (cohesion protein component) *Rad21* indicate that spermatogonia undergo dramatic epigenetic changes [24]. The GO terms enriched among the specifically expressed genes in each cell type corresponded with known biological processes. For example, the genes expressed in elongating cells were related mostly to supramolecular fiber organization, actin filament organization, protein polymerization and spermatid development and differentiation (Figure 2C), which are clearly associated with the formation of the sperm flagellum [4].

We identified differentially expressed genes (DEGs) between normal and hypoxia‐treated cells for all putative cell types and subtypes. More DEGs were observed among spermatocyte cluster B, round spermatids and elongating spermatid cluster A (Supplementary Figure S3a A–G), indicating that the stages were strongly affected by hypoxia treatment, especially the transition from the round spermatid stage to the elongating spermatid stage. Several genes, such as *Mkks*, *Lmbr1*, *Asxl2* and *Cep162*, were shared among the different stages. We noted an interesting expression pattern of ASXL2 during spermatogenesis. Loss of *Asxl2* is associated with increased chromatin accessibility in leukemia [25], and this factor interacts with PRC2 to facilitate H3K27me3 enrichment [26]. We observed that under normal conditions, ASXL2 expression decreased gradually from the spermatogonial stage to the spermatocyte stage, suddenly increased at the round spermatid stage, and then decreased gradually (Figure 2H). However, in hypoxia‐treated cells, *ASXL2* expression decreased throughout spermatogenesis, with a dramatic difference in expression between normal and hypoxic conditions, especially in the round spermatid stage. We further investigated the correlation between cell attributes (expressed gene number and total UMI counts) and *ASXL2* expression to determine whether dysregulation of *ASXL2* leads to poor establishment of H3K27me3 and subsequent failure of chromatin compaction in hypoxic elongating spermatids compared with normal elongating spermatids. Interestingly, for those cells expressing *ASXL2*, the Gini index, expressed gene numbers and UMI counts were strongly negatively correlated with *ASXL2* expression levels (Figure 2I), indicating the role of *ASXL2* in inhibiting gene expression. To further substantiate these findings and verify the accuracy of our cell sorting system for spermatogenic cells, we utilized immunofluorescence staining and Sequential IF/smFISH. These methodologies effectively confirmed the precise identification of the sorted cell populations, including spermatogonia, primary and secondary spermatocytes, round and elongating spermatids, and Sertoli cells (Supplementary Figure S3b H–J). The above scRNA‐seq results indicated that *ASXL2* was downregulated and *CEP162* expression was upregulated under hypoxic conditions, and we further confirmed these observations via Western blot analysis of elongating and round spermatids and mixed seminiferous tubule cells from rats exposed to hypoxia (Figure 2J). These results revealed that under hypoxic conditions, during the transition from round to elongated spermatids, there was a significant decrease in the transcription level of *ASXL2* and a significant increase in the transcription level of *CEP162*. These two genes may act as key regulatory factors that participate in the regulation of the differentiation process from round to elongated spermatids, thereby influencing spermatogenesis.

### 2.3 ASXL2 Directly Interacts With EZH2 to Regulate Spermatogenesis

**FIGURE 3:**
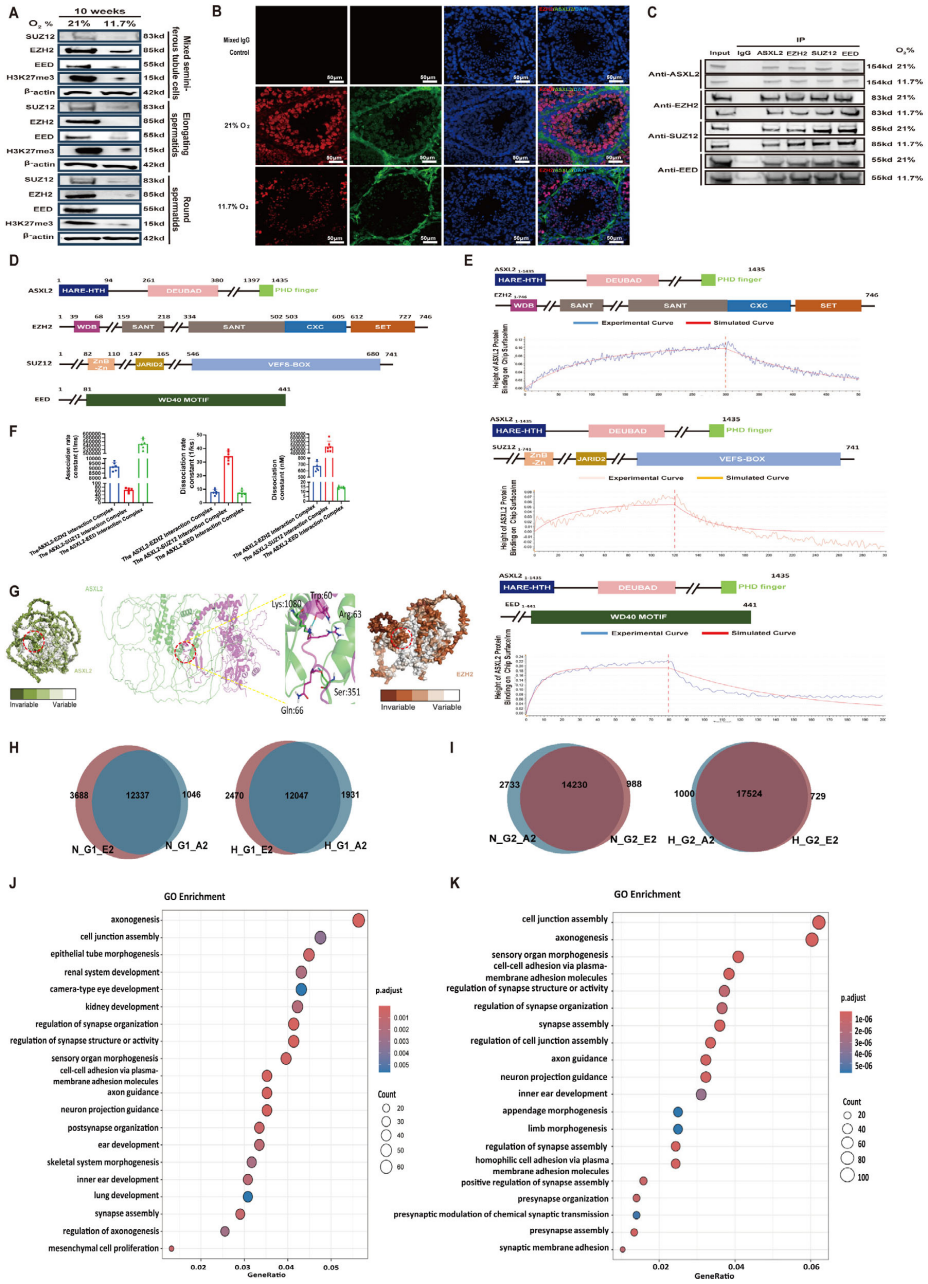
ASXL2 recruits PRC2 via direct interactions with EZH2, SUZ12, and EED. The rats were exposed to 11.7% O_2_ for 10 weeks, after which the testes were harvested for various assays. (A) Western blot analysis of the protein expression of SUZ12, EZH2, and EED in lysates of mixed seminiferous tubule cells, elongating spermatids, and round spermatids from rats housed under normal conditions or hypoxic conditions for 10 weeks (n = 3). (B) Immunofluorescence analysis of the colocalization of ASXL2 and EZH2. The 488 nm channel was used to detect ASXL2 staining (green), and the 555 nm channel was used to detect EZH2 staining (red). (C) Co‐IP assay for ASXL2 and PRC2. Total proteins were extracted from the testis tissues of rats under hypoxic conditions and control rats, and co‐IP assays were performed by using the indicated antibodies, followed by Western blot assays using the indicated antibodies. EZH2, SUZ12 and EED are subunits of PRC2. (D) Overview of the structural components of ASXL2, EZH2, SUZ12, and EED. (E) A biolayer interferometry assay was used to analyze the binding affinity of ASXL2 for EZH2, SUZ12, and EED. The representative figure shows the sensorgrams of purified ASXL2 protein immobilized on a biosensor chip. (F) Representative graphs depicting the association rate constants (K_on), dissociation rate constants (K_off), and dissociation constants (K_D) for the interactions of ASXL2 with EZH2, SUZ12, and EED (n = 6). Note: K_on represents the association rate, K_off denotes the dissociation rate, and K_D indicates the affinity, which is defined as the ratio of K_on to K_off. Kd represents the reciprocal of the KD value. The data are presented as the means ± SDs. (G) The panel shows both the docking of ASXL2 with EZH2 and the subsequent hydrogen bonds formed after docking. Genome‐wide colocalization of the ASXL2 and PRC2 target genes. (H) G1 and (I) G2 cells were exposed to 1% hypoxia for 48 h, and the cells were harvested for CUT&Tag‐seq to reveal the overlap of ASXL2 and EZH2 target genes in G1 and G2 cells. (J) GO analysis of ASXL2 common binding gene loci in rat testis round spermatids under 11.7% vs. 21% oxygen exposure. (K) GO analysis of EZH2 common binding gene loci in rat testis round spermatids under 11.7% vs. 21% oxygen exposure.

Although ASXL1 can recruit PRC2 to target gene loci and downregulate gene expression by facilitating H3K27me3 modification [27], the association between PRC2 and ASXL2 has not yet been reported. However, given the high degree of homology among ASXL family members, ASXL2 may use a mechanism similar to that of ASXL1 to regulate target gene expression [15, 28]. We observed marked downregulation of PRC2 subunits (EZH2, SUZ12 and EED) and H3K27me3 in the elongating and round spermatids and mixed seminiferous tubule cells of the rats exposed to hypoxia (Figure 3A). Immunofluorescence assays revealed that the proteins ASXL2 and EZH2 — the catalytic methyltransferase subunit of PRC2 responsible for H3K27 di‐ and trimethylation — exhibit clear nuclear colocalization in germ cells of rat seminiferous tubules under normoxic conditions (21% O_2_). This colocalization is still detectable under hypoxia (11.7% O_2_), although the signal intensity for both proteins is markedly reduced (Figure 3B). Co‐IP assays further confirmed the mutual interaction between the ASXL2 and PRC2 subunits in rat testis tissue under both normal and hypoxic conditions (Figure 3C).

To investigate whether ASXL2 directly interacts with three subunits of the PRC2 complex (EZH2, SUZ12, and EED), we used biolayer interferometry (BLI) to assess the binding affinity between ASXL2 and each subunit of the PRC2 complex. Our findings revealed that ASXL2 can indeed bind directly to EZH2, SUZ12, and EED, with binding affinities decreasing in the order EED > EZH2 > SUZ12. Consistent with these affinity rankings, the strength of the interactions was also found to follow the same pattern. (Supplementary Figure S4a A, Figure 3D–F). We subsequently used the molecular docking software PyMOL to predict hydrogen bonding between ASXL2 and each of the subunits. Our analysis revealed the presence of hydrogen bonds between ASXL2 and all three subunits, suggesting potential direct interactions (Supplementary Figure S4a B,C, Figure 3G). Furthermore, molecular dynamics simulations confirmed the existence of a stable binding interface between ASXL2 and EZH2 (Supplementary Figure S4a D–F).

To further investigate whether ASXL2 functions by associating with PRC2, we examined how ASXL2 target genes and EZH2 target genes overlap. CUT&Tag‐seq was performed as previously described [29] in the murine spermatogonial cell line G1 and the spermatocyte cell line G2. The results revealed that the target genes of ASXL2 and the target genes of EZH2 largely overlapped in G1 and G2 cells under both hypoxic and nonhypoxic conditions (Figure 3H,I). Moreover, hypoxia induces the expression of many unique genes. For example, after exposure to 1% hypoxia for 48 h, the expression of 2285 unique target genes of ASXL2 (Supplementary Figure S4a H and Supplementary Figure S4b I) and 3781 unique target genes of EZH2 (Supplementary Figure S4b K) was induced in G2 cells. GO analysis further demonstrated that hypoxia‐induced expression of these unique ASXL2 and EZH2 target genes is involved in several biological events, among which genes responsible for cell projection and plasma membrane‐bound cell projection were strongly induced (Supplementary Figure S4b J,L), indicating that ASXL2 and EZH2 might be deeply involved in the sharp morphological changes in spermatogenic cells, that is, the round‐elongating cell transition for the enflagellation of sperm. These biological events might be mediated by several pathways, as predicted by KEGG enrichment analysis in G2 cells (Supplementary Figure S4b M,N). Interestingly, we also observed changes in G1 cells (Supplementary Figure S4a G, S4b O–T), similar to those in G2 cells, suggesting that spermatogenic cells have an inherent program for committed differentiation to spermatids. Notably, in primary round spermatids, GO enrichment analysis revealed that ASXL2 and EZH2 binding sites were enriched in biological processes associated with cytoskeletal remodeling, such as “cell junction assembly” and “axonogenesis” (Figure 3J,K). To further validate the physical co‐occupancy of ASXL2 and EZH2 on chromatin in primary round spermatids, we performed co‐IP of chromatin‐immunoprecipitated complexes (co‐IP of ChIP‐seq). The results, visualized by Venn diagrams (Supplementary Figure S4b U), confirmed significant genome‐wide cobinding of ASXL2 and EZH2. Importantly, this co‐occupancy included loci implicated in cytoskeletal remodeling processes identified in our GO analysis, emphasizing their cooperative role in regulating spermatid morphological transitions.

These findings confirm that ASXL2 can directly interact with all three subunits of PRC2—EZH2, SUZ12, and EED—with the highest affinity for EED. Furthermore, ASXL2 and EZH2, as transcription factors, coregulate gene loci involved in the transition from round to elongated spermatids, which is a critical process in the formation of sperm flagella during spermatogenesis.

### 2.4 The ASXL2–EZH2 Complex is Responsible for Hypoxia‐Induced Microtubule Instability

**FIGURE 4:**
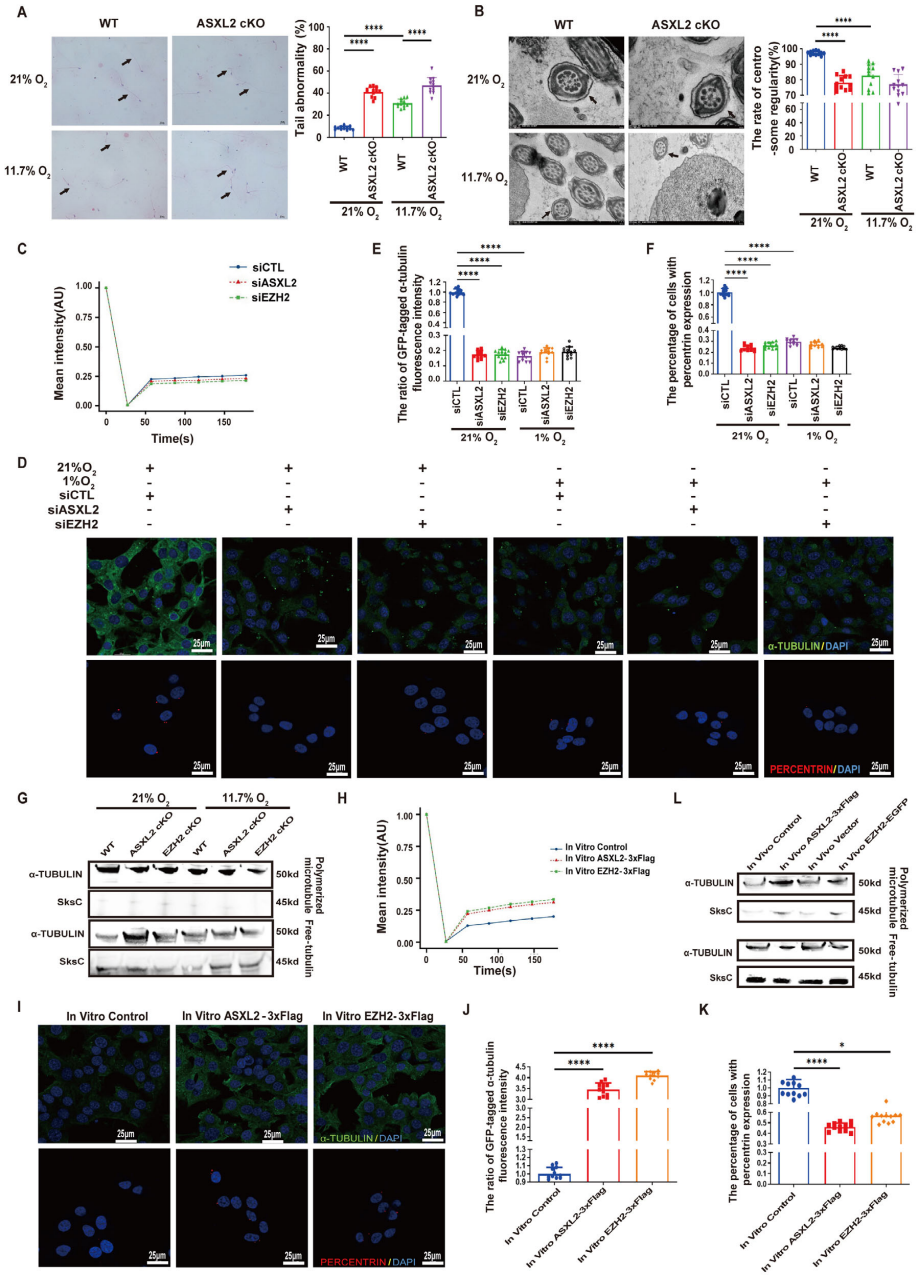
Exogenous activation of the ASXL2–EZH2 axis attenuates hypoxia‐induced microtubule instability. (A) Observation of the morphology of the sperm flagella using Papanicolaou staining (×400). Quantitative analysis of differences in tail abnormalities between WT and ASXL2^−/−^ mice (n = 12). (B) TEM image of sperm with flagella. Cross‐sectional TEM analysis of spermatozoa from ASXL2^−/−^ mice. Missing DMTs are indicated by black arrows. Additionally, a quantitative analysis of centrosome regularity was performed in ASXL2^−/−^ and WT mice (n = 12). (C) At 48 h after knockdown of the endogenous expression of ASXL2 and EZH2 in G2 cells cultured under 1% O_2_, FRAP technology was used to assess the dynamics of microtubule polymerization and depolymerization in these cells (n = 6). (D) Microtubule regrowth experiments were used to analyze the effects of ASXL2 and EZH2 interference on microtubule stability in G2 cells. Representative images illustrate (E) the ratio of GFP‐tagged α‐tubulin fluorescence intensity (n = 12) and (F) the percentage of cells with percentrin expression (n = 12). (G) Western blot analysis was conducted to examine the levels of free tubulin and polymerized microtubules in spermatogenic cells derived from ASXL2^−/−^ and EZH2^−/−^ mice. The samples were fractionated into soluble (S) (containing free tubulins) and insoluble (P) (containing microtubules) fractions, followed by immunoblot analysis with antibodies against a‐tubulin and SksC. G2 cells were transfected with In Vitro ASXL2‐3xFlag and In Vitro EZH2‐3xFlag under 1% O2 conditions for 48 h. (H) FRAP analysis was used to assess and compare the microtubule polymerization and depolymerization rates in G2 cells overexpressing ASXL2 and those overexpressing EZH2 under hypoxic conditions of 1% O_2_ (n = 6). (I) A microtubule regrowth assay was used to analyze the stability of microtubules in G2 cells under hypoxia following the overexpression of ASXL2 and EZH2. The quantitative data presented in the figures include (J) the ratio of GFP‐tagged α‐tubulin fluorescence intensity (n = 12) and (K) the percentage of cells with percentrin expression (n = 12). The mice were exposed to 11.7% oxygen for 10 weeks and injected with In Vivo ASXL2‐3xFlag or In Vivo EZH2‐3xFlag. (L) Western blot analysis was performed to assess the levels of free tubulin and polymerized microtubules in spermatogenic cells from mice overexpressing ASXL2 and EZH2. The data are presented as the means ± SDs; *p < 0.05, ****p < 0.0001; 1‐way ANOVA followed by Tukey's post hoc test (J, K); ***p < 0.0001; 2‐way ANOVA followed by Tukey's post hoc test (A, B, E, F).

The results from the above experiments revealed a correlation between downregulated ASXL2/PRC2 expression and upregulated CEP162 expression under hypoxia. We therefore next investigated whether ASXL2 knockout or EZH2 knockout could simulate the detrimental effects of chronic hypoxia exposure on spermatogenesis. We established spermatogenic cell‐specific conditional ASXL2‐knockout (KO) mice, which presented significantly impaired sperm motility parameters, including sperm motility, curvilinear velocity, straight‐line velocity, average path velocity, beat‐cross frequency and amplitude of lateral head displacement, compared with those of wild‐type control mice (Supplementary Figure S5A,B,E). Similarly, spermatogenic cell‐specific conditional EZH2‐KO mice presented reduced sperm motility parameters and tail abnormalities. Similar phenotypes were observed in ASXL2^−/−^ and EZH2^−/−^ mice under hypoxia (Supplementary Figure S5C,D,E). Furthermore, sperm flagella were prone to deformities and incomplete centrosome structures in ASXL2^−/−^ and EZH2^−/−^ mice (Supplementary Figure S5F,G, Figure 4A,B). We subsequently conducted FRAP and microtubule regrowth assays to investigate the impact of interference with endogenous ASXL2/EZH2 expression on microtubule polymerization and depolymerization in G2 cells. These findings revealed that disrupting the endogenous expression of ASXL2/EZH2 in G2 cells markedly slowed microtubule polymerization under hypoxic conditions, leading to microtubule instability (Figure 4C–F). Notably, under both normoxic and hypoxic conditions, we observed an increase in the proportion of free tubulin and polymerized microtubule proteins in the spermatogenic cells of ASXL2‐null and EZH2‐null mice. These findings suggest increased susceptibility to microtubule instability in ASXL2‐null and EZH2‐null mice (Figure 4G).

To gain further insight into the crucial role of the activated ASXL2–EZH2 axis in hypoxia‐induced aberrant spermatogenesis, we used an adenovirus to induce spermatogenic cell‐specific activation of endogenous ASXL2/EZH2 expression and examined the effect on spermatogenesis. Successful activation of the ASXL2–EZH2 axis was confirmed by staining testis slices from mice 3 weeks after virus injection (Supplementary Figure S5H,I). The results of the sperm motility assay demonstrated that activation of the ASXL2–EZH2 axis significantly alleviated the impairments in sperm motility parameters (Supplementary Figure S5J). We found that activation of the ASXL2–EZH2 axis significantly increased sperm penetration (Supplementary Figure S5K) and significantly inhibited sperm flagellar abnormalities (Supplementary Figure S5L). Notably, overexpression of ASXL2 and EZH2 led to a marked increase in the rate of microtubule polymerization, resulting in improved microtubule stability (Figure 4H–K). Next, we aimed to determine whether an increased proportion of polymerized and free microtubule proteins was responsible for the diminished sperm abnormalities observed in ASXL2/EZH2‐overexpressing cells. We detected the expression of polymerized microtubules and free tubulin in spermatogenic cells. Western blot analysis revealed that there was less free tubulin and significantly more polymerized microtubules in spermatogenic cells following the activation of ASXL2/EZH2 (Figure 4L). In summary, our findings demonstrate that inhibiting the ASXL2–EZH2 axis exacerbates hypoxia‐induced microtubule instability, whereas activating the ASXL2–EZH2 axis can ameliorate this instability. These analyses of the mechanisms by which the ASXL–EZH2 axis influences cellular microtubule dynamics under hypoxic conditions highlight the importance of this axis in maintaining microtubule stability.

### 2.5 The ASXL2–EZH2 Complex Counteracts Hypoxia‐Induced Microtubule Destabilization by Downregulating CEP162 Expression

Our scRNA‐seq data revealed that CEP162, a protein associated with the axoneme, was significantly upregulated in elongating spermatids, as shown in Figure 2H. Misexpression of CEP162 has been linked to cilia malformations [13], and it may at least partially account for the notably high incidence of flagellum deformities observed in Figure 1. Western blot assays confirmed that CEP162 protein expression was markedly increased in both elongating and round spermatids from rats exposed to hypoxic conditions for 10 weeks compared with those from rats under normal conditions. This upregulation was accompanied by significant downregulation of the suppressive ASXL2/PRC2 axis due to hypoxia, as depicted in Figure 2J and Figure 3A. Consequently, we investigated whether the downregulation of the ASXL2/PRC2 axis could be responsible for the significant upregulation of CEP162 expression.

**FIGURE 5:**
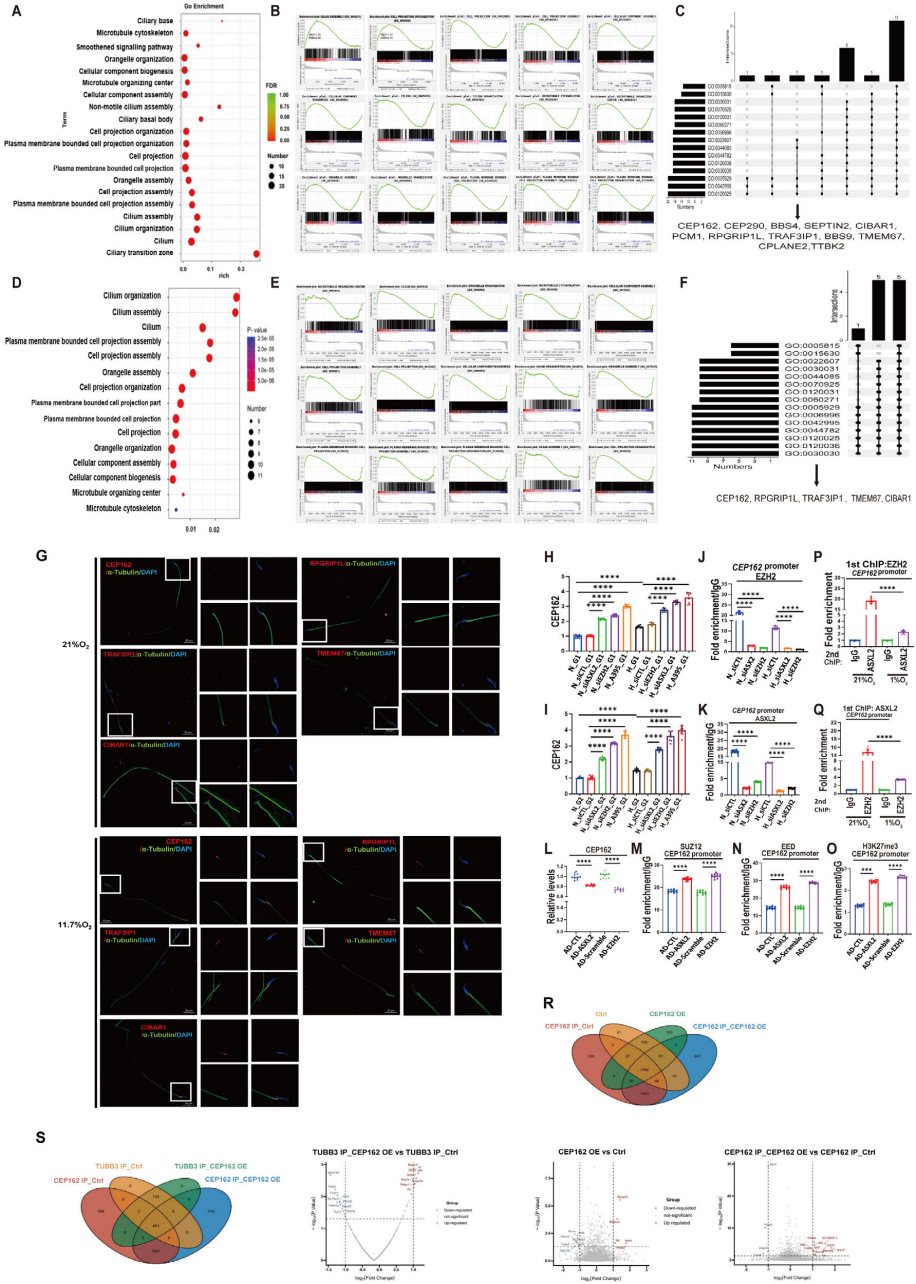
The ASXL2–EZH2 complex mitigates the destabilizing effects of hypoxia on microtubules through the downregulation of CEP162. WT, ASXL2^−/−^ and EZH2^−/−^ mice were housed under normal (21% O2 content) or hypoxic (11.7% O2) conditions for 10 weeks, the testes and epididymides were harvested, and spermatogenic cells from the testis and sperm from the epididymis were separated via digestion. (A) GO enrichment analysis of spermatogenesis‐related signaling pathways that were differentially expressed in primary spermatogenic cells between WT and ASXL2^−/−^ mice. The 15 GO pathways marked in red are the biological processes enriched by CEP162 (n = 3). (B) Gene set enrichment analysis (GSEA) of biological processes enriched by CEP162 in spermatogenic cells of ASXL2^−/−^ mice, including those associated with the microtubule cytoskeleton, organelle organization, cellular component biogenesis, microtubule organizing center, cellular component assembly, cell projection organization, plasma membrane‐bound cell projection organization, cell projection, plasma membrane‐bound cell projection, organelle assembly, cell projection assembly, plasma membrane‐bound cell projection assembly, cilium assembly, cilium organization and cilium. (C) The UpSet plot illustrates the overlap of 12 core genes upregulated across the aforementioned 15 biological processes. (D) GO enrichment analysis was performed on the 15 biological processes associated with the differentially expressed spermatogenesis‐related signaling pathways shown in (A) in primary spermatogenic cells from WT and EZH2^−/−^ mice. I GSEA of 15 biological processes in primary spermatogenic cells from EZH2^−/−^ mice. (F) The UpSet plot was used to cross‐analyze the 12 genes featured in (C) across 15 biological processes in primary spermatogenic cells from EZH2^−/−^ mice, thereby identifying the genes with upregulated transcription. (G) Immunofluorescence analysis was conducted on rat sperm exposed to 21% and 11.7% oxygen for 10 weeks to examine the expression of CEP162, RPGRIP1L, TRAF3IP1, TMEM67, and CIBAR1 within spermatozoa. ASXL2/EZH2‐deficient G1 and G2 cells were subjected to 1% oxygen conditions for 48 h. Quantitative PCR (qPCR) was used to measure the mRNA levels of CEP162 in both control and ASXL2/EZH2‐deficient (H) G1 (n = 10) and (I) G2 cells (n = 10). mRNA expression levels in the treatment groups were normalized against β‐actin levels and are expressed as the ratio of normalized mRNA expression to that in the control cells. Chromatin immunoprecipitation (ChIP) followed by qPCR was conducted to analyze the binding of (J) EZH2 (n = 10) and (K) ASXL2 (n = 10) to the CEP162 promoter in G2 cells treated with the control, ASXL2 siRNA, or EZH2 siRNA. In G2 cells, the overexpression of exogenous ASXL2/EZH2 was achieved through adenoviral transfection. (L) qPCR was also utilized to compare CEP162 mRNA levels between the control group and the AD‐ASXL2/EZH2 group in G2 cells. The mRNA expression levels were normalized to those of β‐actin and are expressed as the ratio of normalized mRNA expression to that of the control cells (n = 10). ChIP‒qPCR analysis was performed to assess the occupancy of (M) SUZ12 (n = 10), (N) EED (n = 10), and (O) H3K27me3 (n = 10) on the CEP162 promoter in G2 cells treated with control, AD‐ASXL2, or AD‐EZH2. Sequential ChIP‒qPCR analysis was conducted to determine the colocalization of (P) ASXL2 (n = 10) and (Q) EZH2 (n = 10) on the CEP162 promoter in G2 cells under normoxic and hypoxic conditions. The first and second ChIP antibodies used are indicated in the chart titles and x‐axis labels, respectivelI(R) After overexpression of CEP162 in spermatogenic cells, the increased binding of tubulin dimers obtained via CEP162 IP‒MS intersects with the decreased distribution of tubulin dimers in cilia. (S) In spermatogenic cells overexpressing CEP162, TUBB3 IP‐MS was used to identify the TUBA3A that exhibited binding to it, and the TUBA3A that bound to CEP162 was used to complement the TUBA3A that exhibited binding to TUBB3. The data are presented as the means ± SDs; ****p < 0.0001; 1‐way ANOVA followed by Tukey's post hoc test (P, and Q); ***p < 0.001, ****p < 0.0001; 2‐way ANOVA followed by Tukey's post hoc test (H‐O).

GO enrichment analysis of primary spermatogenic cells from ASXL2^−/−^ mice revealed significant differences in 15 biological processes compared with those in the wild‐type control group, including those associated with the microtubule cytoskeleton, organelle organization, cellular component biogenesis, microtubule organizing center, cellular component assembly, cell projection organization, plasma membrane‐bound cell projection organization, cell projection, plasma membrane‐bound cell projection, organelle assembly, cell projection assembly, plasma membrane‐bound cell projection assembly, cilium assembly, cilium organization and cilium (Figure 5A). Additionally, CEP162 was identified as a core gene in these differentially regulated biological processes and was significantly upregulated in ASXL2^−/−^ primary spermatogenic cells (Figure 5B). Among the upregulated genes, we identified 12 common genes among the core genes of the 15 differential biological processes, including CEP162, as well as RPGRIP1L, TMEM67, CEP290, PCM1, CPLANE1, TTBK2, CIBAR1, and NPHP8 (Figure 5C). Furthermore, among the 15 overlapping biological process genes identified through transcriptome sequencing of primary spermatogenic cells from EZH2^−/−^ mice, five genes—CEP162, RPGRIP1L, TRAF3IP1, TMEM67, and CIBAR1—displayed significant transcriptional upregulation among the 12 identified common genes (Figure 5D–F). Additionally, our immunofluorescence assays confirmed that the expression levels of these five proteins were elevated in the sperm of hypoxic mice, compared with those of normoxic wild‐type mice (Figure 5G).

We observed similar results in G1 and G2 cells exposed to hypoxia. Interfering with endogenous ASXL2/EZH2 expression with siRNA markedly upregulated the transcription level of CEP162/RPGRIP1L in hypoxic G1 and G2 cells. Moreover, treating G1/G2 cells with A395, a specific antagonist of the PRC2 complex that disrupts the interaction between the PRC2 subunits EZH2‐EED‐SUZ12, significantly increased the transcription level of CEP162/TRAF3IP1 (Supplementary Figure S6A,B, Figure 5H,I), which further supports the regulatory network between ASXL2‐PRC2‐CEP162. Furthermore, knockdown of ASXL2 reduced EZH2 binding to the CEP162/RPGRIP1L promoter. In addition, knockdown of EZH2 inhibited the binding of ASXL2 to the CEP162/RPGRIP1L promoter, indicating the dependency of ASXL2 on EZH2 at the same promoter (Supplementary Figure S6C,D, Figure 5J,K). Conversely, our findings revealed that exogenous overexpression of ASXL2/EZH2 led to a decrease in the transcription level of CEP162/RPGRIP1L in G2 cells (Supplementary Figure S6E, Figure 5L). Additionally, we observed a corresponding decrease in the binding of SUZ12, EED, and H3K27me3 within the promoter region of the gene encoding CEP162/RPGRIP1L (Supplementary Figure S6F–H, Figure 5M–O). To demonstrate the co‐occupancy of ASXL2 and EZH2 on the same promoter, we performed pairwise sequential ChIP‒qPCR analyses. After the first round of EZH2 immunoprecipitation, ASXL2 binding was also enriched in the EZH2‐bound CEP162 and RPGRIP1L promoters under normoxia. However, the colocalization of these regulators on the CEP162/RPGRIP1L promoter dynamically changed under hypoxia and ASXL2 enrichment was markedly reduced. We found similar results after changing the order of immunoprecipitation (Supplementary Figure S6I,J, Figure 5P,Q). These findings suggest that EZH2 and ASXL2 colocalize at the promoters of cilium assembly related markers, such as CEP162 and RPGRIP1L, where they reciprocally regulate the expression of these markers.

Currently, ciliary components, including CEP162 and CPLANE1, influence spermatogenesis by regulating the permeability of the transition zone. The literature shows that perturbation of CEP162 results in incorrect mitotic spindle organization or orientation [30], suggesting that CEP162 regulates microtubule stability in vivo. In our study, we initially investigated whether CEP162 directly influences tubulin cleavage and polymerization. Our results revealed that CEP162 had no effect on microtubule assembly or cleavage (Supplementary Figure S6K). Next, we explored whether CEP162 competes with tubulin for binding to α‐/β‐tubulin dimers and affects microtubule stability. We first overexpressed CEP162 in spermatogenic cells and then identified proteins associated with increased binding to tubulin dimers through CEP162 immunoprecipitation‒mass spectrometry (IP‒MS). We subsequently used proteomics sequencing to screen for tubulin dimer proteins with reduced distribution in sperm cilia overexpressing CEP162. The intersection of these two sets of tubulin dimers led us to investigate the role of TUBB3 (Supplementary Figure S6L, Figure 5R). Using spermatogenic cells overexpressing CEP162, we conducted TUBB3 IP‐MS to identify tubulin with decreased binding. We analyzed a subset of tubulin proteins that exhibited reduced binding to TUBB3 but were previously bound by CEP162, revealing that TUBA3A proteins no longer associated with CEP162 after overexpression of CEP162 but exhibited reduced binding to TUBB3 (Figure 5S). Collectively, these findings indicate that the activation of the ASXL2–EZH2 signaling pathway increases the activity of the H3K27 histone methyltransferase, which in turn suppresses CEP162 gene expression. This downregulation of CEP162 facilitates the efficient trafficking of TUBB3 from the cytoplasm to cilia, which is mediated by TUBA3A. Consequently, this mechanism helps to counteract the destabilizing effects of hypoxia on microtubules within the germline and spermatogenic cells of rats.

### 2.6 ASXL2 Recruits EZH2 to the CEP162 Promoter Region Spanning 3482–3511 bp to catalyze H3K27me3 Modification

ASXL1 has been reported to recruit PRC2 to specific genomic loci, where it plays a role in adding the repressive H3K27me3 modification, which in turn can recruit PRC1 for transcriptional repression [31]. ASXL1 interacts with components of PRC2, including EZH2 and SUZ12, and this interaction is crucial for the recruitment of PRC2 and subsequent H3K27me3 modification [32]. Given the structural and functional similarities between ASXL1 and ASXL2, ASXL2 may function like ASXL1 in recruiting PRC2, particularly EZH2, to the H3K27 site on nucleosomes to catalyze the formation of highly methylated modifications.

**FIGURE 6:**
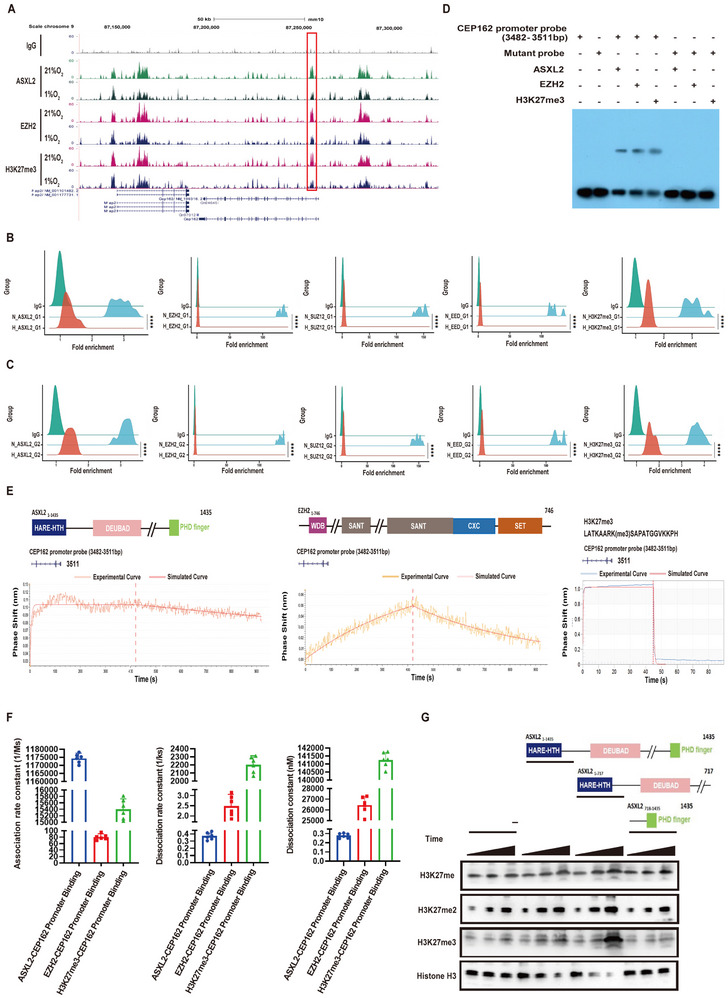
ASXL2 recruits EZH2 to the 3482–3511 bp segment of the CEP162 promoter, where it mediates H3K27me3 epigenetic modification. (A) In G2 cells exposed to hypoxia, snapshots of CUT&Tag‐Seq traces for ASXL2, EZH2, and H3K27me3 on the promoter region of the CEP162 coding gene were captured. The FASTQ reads were aligned to the mouse reference genome mm10 using Bowtie [33], and the alignment results were then converted into BigWig files to generate signal tracks. These tracks were ultimately visualized via the UCSC Genome Browser. (B) G1 cells were exposed to 21% oxygen and 1% oxygen for 48 h. A ChIP‒qPCR assay was performed with the indicated antibodies to detect the enrichment of ASXL2 (n = 10), EZH2 (n = 10), SUZ12 (n = 10), EED (n = 10) and H2K27me3 (n = 10) at the CEP162 promoter. (C) G2 cells were exposed to 21% oxygen and 1% oxygen for 48 h. ChIP‒qPCR was used to detect the enrichment of ASXL2 (n = 10), EZH2 (n = 10), SUZ12 (n = 10), EED (n = 10) and H2K27me3 (n = 10) at the CEP162 promoter. ASXL2, EZH2, SUZ12, EED and H3K27me3 levels were normalized to the ChIP data for IgG. (D) EMSA were performed to evaluate the binding of ASXL2, EZH2, and H3K27me3 to the CEP162 promoter region at 3482–351Ip. (E) Biolayer interferometry assays were conducted to analyze the binding affinities of ASXL2, EZH2, SUZ12, EED, and H3K27me3 for the CEP162 promoter region, which spans from 3482 to 3511 bp. (F) The graphs illustrate the interaction kinetics of the CEP162 promoter segment (3482–3511 bp) with ASXL2, EZH2, and H3K27me3, including the association rate constants (K_on), dissociation rate constants (K_off), and dissociation constants (K_D) (n = 6). (G) Anti‐H3K27me/H3K27me2/H3K27me3/Histone H3 Western blot showing that the ASXL2‐N truncated fragment but not the ASXL2‐C truncated fragment of ASXL2 could stimulate EZH2 activity in a methylation assay against K27‐methylated H3 in oligonucleosomes (time points: 0, 10, and 20 min). The reactions involving only EZH2 are indicated with ‘−’. The data are presented as the means ± SDs; ****p < 0.0001; 1‐way ANOVA followed by Tukey's post hoc test (B, and C).

CUT&Tag‐Seq analysis revealed that CUT&Tag‐Seq signaling is attenuated by hypoxia exposure within a genome‐wide region centered 6 kb from the transcriptional start site of the ASXL2, EZH2 and H3K27me3 genes (Supplementary Figure S7A,B). A ChIP‒qPCR assay demonstrated that ASXL2, EZH2, SUZ12 and EED were enriched in the CEP162 promoter region under normoxic conditions. However, the colocalization of these regulators on the CEP162 promoter was markedly reduced under hypoxic conditions. Consequently, the inhibitory H3K27me3 modification of CEP162 was significantly downregulated by hypoxia (Figure 6B,C). Building upon our previous findings, we conducted CUT&Tag‐Seq analysis of the mm10 genome and discovered that under hypoxic conditions, G2 cells exhibited a reduction in the binding of ASXL2, EZH2, and H3K27me3 to the CEP162 promoter region spanning nucleotides 3482–3511 (Figure 6A). Furthermore, our electrophoretic mobility shift assay (EMSA) revealed that ASXL2 and EZH2, along with H3K27me3, can directly interact with the CEP162 promoter region at nucleotides 3482–3511 (Supplementary Figure S7C,D, Figure 6D). BLI analysis also revealed that ASXL2, EZH2, and H3K27me3 had dissociation constants of 0.284, 26 544.5, and 14 1316.7 (Ms)^−1^, respectively, when they interacted with the CEP162 promoter probe at 3482–3511 bp (Supplementary Figure S7E, Figure 6E,F). Furthermore, after treating oligonucleosomes with KDM2B, which is a demethylase specific for histone H3 at the K27 residue, we observed an increase in mono‐, di‐, and trimethylation of histone H3K27 when ASXL2 was cotreated with EZH2 compared with that of oligonucleosomes treated with EZH2 alone. Concurrently, there was a decrease in nonmethylated histone H3 (Supplementary Figure S7F, Figure 6G). Our findings indicate that ASXL2 can recruit EZH2 to the CEP162 promoter region, specifically between 3482 and 3511 bp, to catalyze the trimethylation of histone H3 at the K27 residue. In contrast, hypoxia promotes the transcription of CEP162 by inhibiting this process.

### 2.7 Clinical Relevance of ASXL2 and EZH2 Expression in Male Infertility Outcomes

Building upon evidence from the MIK public database, which highlights the role of polycomb genes such as *ASXL2* and *EZH2* in male infertility, we further investigated the molecular mechanisms underlying these conditions. Specifically, we focused on teratozoospermia, a condition characterized by sperm with abnormal morphology, for which both *ASXL2* and *EZH2* are implicated in gene expression irregularities and genetic mutations (Figure 7a A).

FIGURE 7(A–D) Teratozoospermia clinical characteristics. (A) The MIK public database (http://mik.bicnirrh.res.in) was used to search for genes reported to be associated with male infertility. Using public GEO datasets, we conducted a comprehensive analysis of gene expression in patients with teratozoospermia and idiopathic NOA. For teratozoospermia, we explored gene expression profiles using PCR array analysis on the (B) Affymetrix platform from GSE6872 (n = 13 for healthy, n = 8 for teratozoospermic) and (C) Illumina platform data from GSE6967 and GSE6968 (n = 9 for normozoospermic, n = 14 for teratozoospermic). In parallel, we investigated gene expression in nonobstructive azoospermia patients through RNA‐seq analysis. For dataset (D) GSE190752 (n = 3 for healthy, n = 3 for NOA), we standardized the expression levels with TPM. All the data are presented as the means ± SDs; ns nonsignificant, *p < 0.05, **p < 0.01, ***p < 0.001, as determined by 2‐tailed, unpaired Student's *t*‐test (D). ns nonsignificant, *p < 0.05, **p < 0.01, ***p < 0.001, ****p < 0.0001, as determined by 1‐way ANOVA with Tukey's post hoc test (B, C).**—** (E–I) Clinical characteristics nonobstructive azoospermia. We analyzedItaset (E) GSE216907 (n = 2 for healthy, n = 8 for NOA), and used TPM for gene expression normalization. In a complementary approach, we examined gene expression data from nonobstructive azoospermia patients using (F) Agilent PCR array platform data from GSE145467 (n = 15 for healthy individuals, n = 5 for NOA patients). In these analyses, the y‐axis represents the gene expression levels within each group. (G) Immunofluorescence staining showing the expression of ASXL2 and EZH2 in seminiferous tubules of testicular tissues from the normozoospermic group and NOA group. Bar charts represent quantification of mean fluorescence intensity per cell, normalized to the ASXL2‐Normozoospermic group. Fluorescence signals were measured per DAPI‐defined nucleus after background subtraction; ≥100 cells were analyzed per sample (n = 12 individuals per group). Scale bar: 50 µm. (H) Immunofluorescence analysis of ASXL2 and EZH2 expression in spermatozoa from normozoospermic and teratozoospermic groups, with relative fluorescence intensity per spermatozoon quantified separately in two subcellular compartments — the sperm head (defined by DAPI staining, representing the nuclear region) and the sperm flagellum (defined by α‐tubulin co‐staining, representing the cytoplasmic tail region) — where mean fluorescence intensity for each compartment was measured and normalized to the average value of the ASXL2‐Normozoospermic group; at least 100 spermatozoa were analyzed per sample (n = 12 individuals per group). scale bar: 10 µm. (I) Western blot detection of ASXL2 and EZH2 protein expression levels in spermatozoa from the normozoospermic group and teratozoospermic group. All the data are presented as the means ± SDs; ns nonsignificant, **p < 0.01, ***p < 0.001, ****p < 0.0001, as determined by 2‐tailed, unpaired StudentI*t*‐test (E). ns nonsignificant, *p < 0.05, **p < 0.01, as determined by 1‐way ANOVA with Tukey's post hoc test (F). ****p < 0.0001, as determined by 2‐way repeated‐measures ANOVA with Tukey's post hoc test (G, H). (J) Schematic model of hypoxia‐induced disruption of spermatogenesis. (K) Schematic diagram showing how hypoxia induces aberrant spermatogenesis. Hypoxia impairs spermiogenesis through two mechanisms: (i) ATP depletion directly inhibits microtubule polymerization; (ii) downregulation of ASXL2 reduces EZH2 binding and H3K27me3 modification at the CEP162 promoter, enabling CEP162 overexpression. Excess CEP162 competes with TUBA3A for TUBB3 binding, depleting ciliary TUBB3 and triggering microtubule collapse. Under normal conditions, the repressive ASXL2 gene interacts with and recruits PRC2 to target genes to mediate H3H27me3 modification to direct elongating spermatid development by inhibiting the expression of genes that should be programmed to be silenced during the transition of round spermatids to elongating spermatids. However, hypoxia exposure markedly downregulates ASXL2 expression, which induces the abnormal expression of genes that should be shut down during the round‐to‐elongating transition, contributing to the accumulation of round spermatids and total reduced sperm counts and significant deformation of the sperm flagellum owing to the abnormal ectopic expression of the CEP162 gene. This model was generated using FigDraw (https://figdraw.com).

**Figure:**
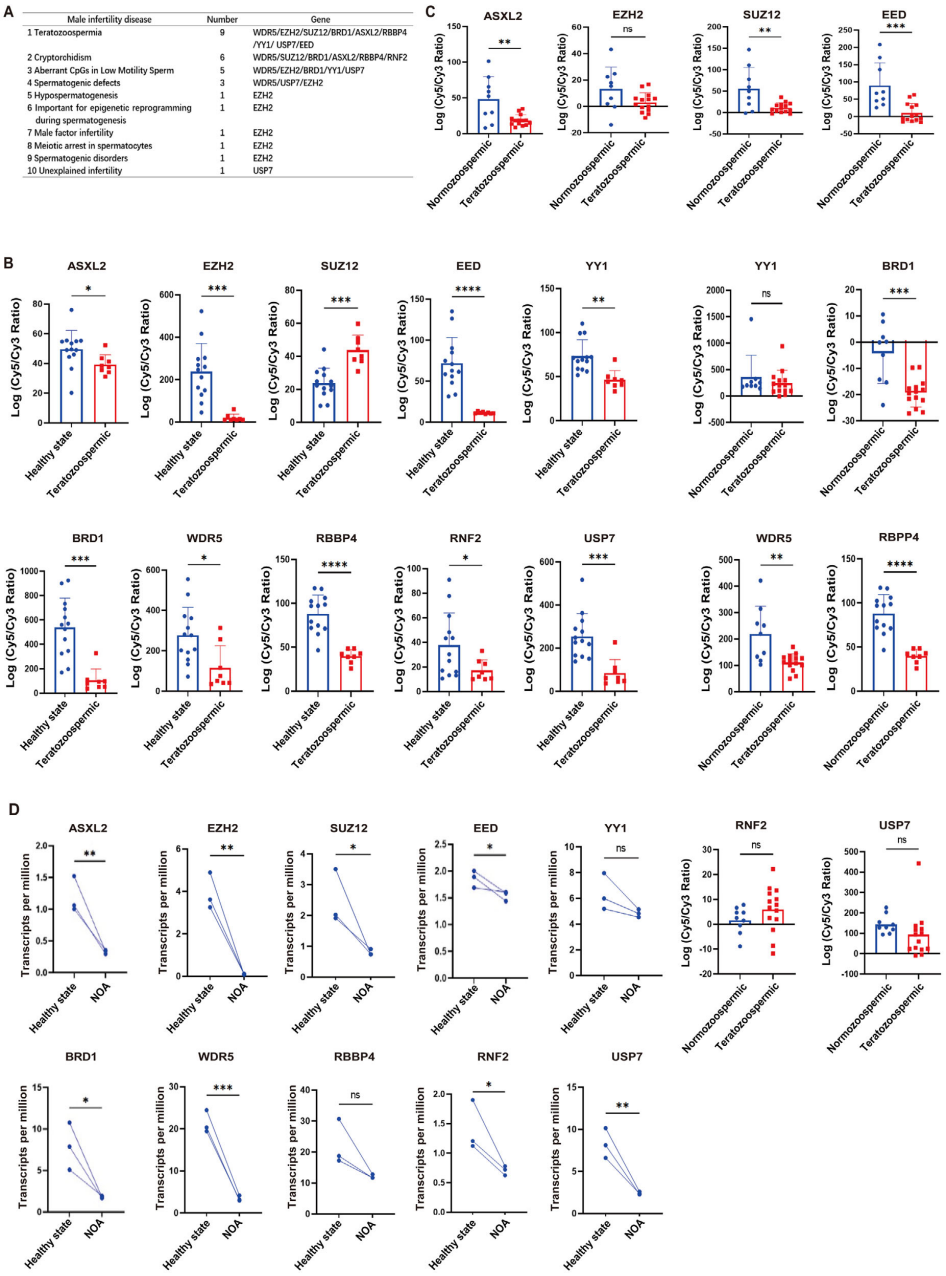

**Figure:**
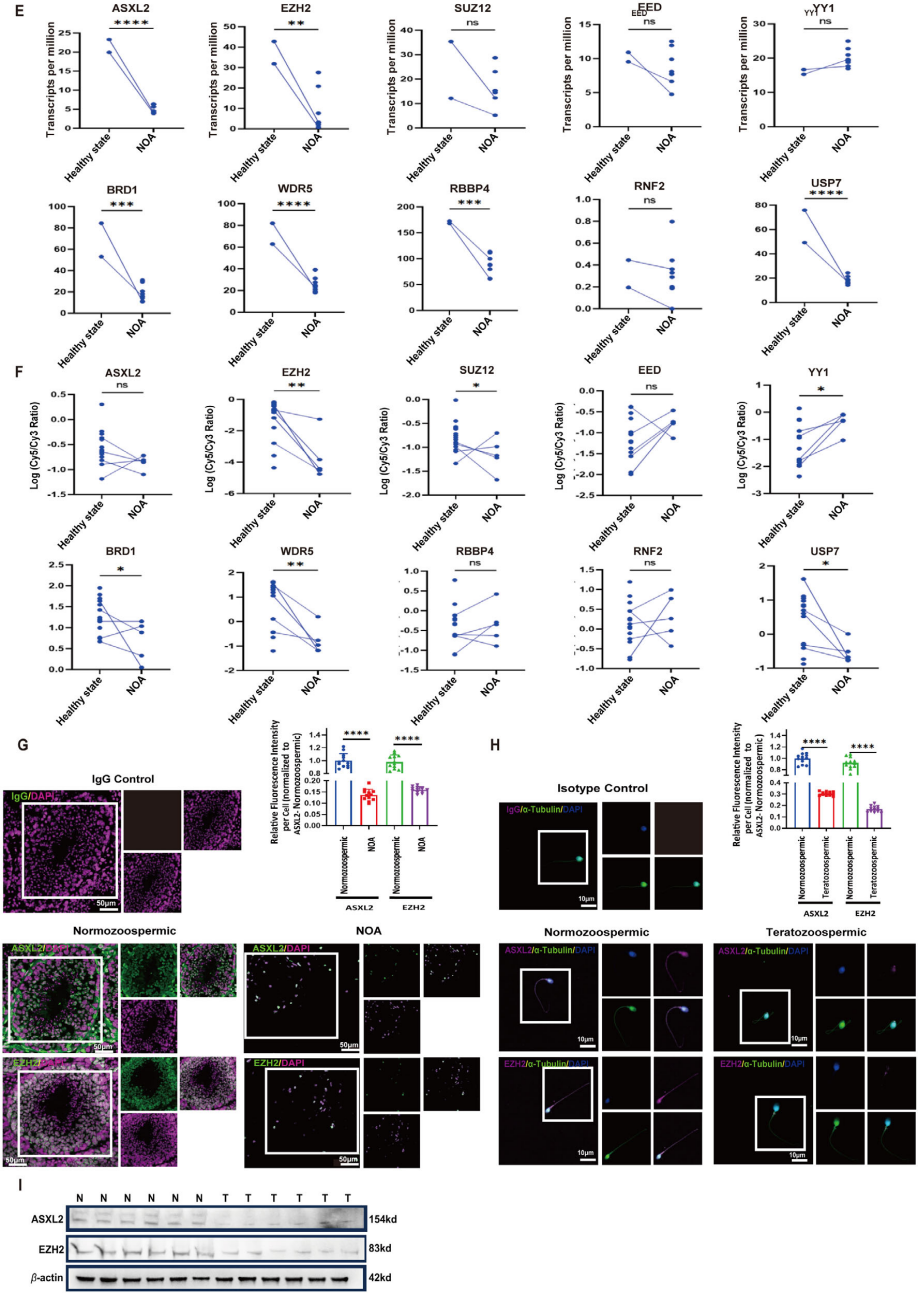

**Figure:**
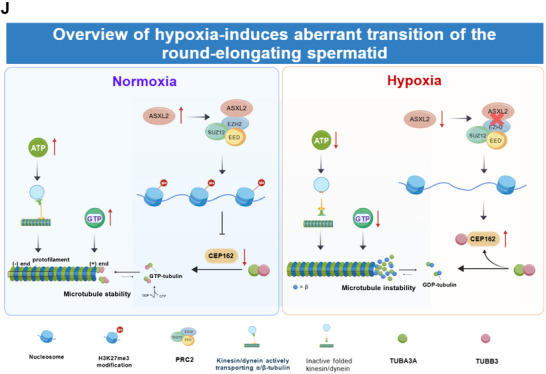

To further elucidate the relationship between these proteins and male infertility, we used the GEO database to analyze the expression patterns of polycomb genes, such as *ASXL2* and *EZH2*, in sperm samples from patients with teratozoospermia and nonobstructive azoospermia. Our analysis revealed that patients diagnosed with teratozoospermia presented decreased transcription levels of *ASXL2* and *EZH2* in their sperm, with a concurrent reduction in the expression of other reported polycomb proteins, including *SUZ12*, *EED*, *YY1*, *BRD1*, *WDR5*, *RBBP4*, and *USP7* (Supplementary Figure S8a A,B, Figure 7a B,C). Moreover, our examination of sperm from nonobstructive azoospermia patients revealed a decrease in the transcription levels of *ASXL2*, *EZH2*, *SUZ12*, *BRD1*, *WDR5*, and *USP7* (Supplementary Figure S8b C–E, Figure 7a D, Figure 7b E,F).

To validate the translational relevance of our findings, we conducted immunohistochemical (IHC) analysis of ASXL2 and EZH2 protein expression in sperm samples from 6 patients with teratozoospermia and 6 normozoospermic controls, as well as in sperm samples from 1 patient with nonobstructive azoospermia (NOA) and 1 normozoospermic control. Additionally, to ensure the accuracy of our pathological subtype classification and rule out the presence of Sertoli cell‐only syndrome (SCOS), we performed immunofluorescence staining for the spermatogonia‐specific marker PLZF in testicular tissue sections from the NOA patient. The presence of PLZF‐positive cells confirmed the existence of spermatogonia within the seminiferous tubules, thereby distinguishing our NOA case from SCOS, which is characterized by a complete absence of germ cells (Supplementary Figure S8b F). Quantitative analysis of immunofluorescence signal intensity—measured within DAPI‐defined nuclei for testicular germ cells and in anatomically delineated sperm subcompartments (head vs. flagellum)—revealed a significant reduction in both ASXL2 and EZH2 protein expression in testicular seminiferous tubules from patients with nonobstructive azoospermia (NOA) compared to normozoospermic controls (Figure 7b G and Supplementary Figure S8c G). Similarly, in spermatozoa from teratozoospermic patients, ASXL2 and EZH2 levels were markedly diminished in both the nuclear (head) and cytoplasmic (flagellum) regions relative to normozoospermic individuals (Figure 7b H). Western blot results also indicated decreased expression of ASXL2 and EZH2 in the sperm of patients with teratozoospermia (Figure 7b I). These findings highlight the importance of polycomb gene dysregulation in the pathophysiology of male infertility and collectively demonstrate that polycomb genes, including *ASXL2* and *EZH2*, exhibit decreased transcriptional activity in sperm from patients with both teratozoospermia and nonobstructive azoospermia. This observation not only bolsters the experimental data from our study but also validates and supports our initial hypothesis that the ASXL2 and EZH2 complex may be critical for male fertility (Figure 7c J).

## 3 Discussion

Spermatogenesis involves a complex network of processes that occur in the seminiferous tubules and culminate in the production of mature male gametes. These processes include the proliferation of spermatogonia, spermatogonial differentiation into spermatocytes, the meiotic division of spermatocytes producing spermatids, the maturation of round spermatids, and the release of highly specialized mature spermatozoa into the testicular tubule lumen [4]. All factors affecting this development process could be causative factors for male infertility. In addition to other known factors associated with male infertility, such as obesity, psychological stress and exposure to environmental pollutants, hypoxia is also a predominant cause [34]. In mammals, two types of hypoxia are known, namely, environmental hypoxia and pathological hypoxia. The former occurs mainly because of the low partial pressure of inhaled oxygen due to high altitude, whereas the latter comprises impaired testicular oxygen delivery or utilization caused by pathologies, such as varicocele [2], chronic lung disease [35], sleep apnea [36] and sickle cell disease [37]. Both types of hypoxia markedly impair male fertility in both animals and humans, resulting in a reduced sperm count, low sperm motility and abnormal sperm morphology or sperm output [3], as evidenced in chronic lung disease patients with systemic hypoxia who present reduced sperm counts and increased sperm DNA fragmentation [38]. These effects may occur due to impairments in key developmental events during early embryonic development.

While current evidence suggests that high‐altitude exposure may affect sperm motility, there is no clear consensus on whether the incidence of asthenospermia differs between high‐ and low‐altitude populations; several studies have reported reduced sperm motility at high altitudes [39, 40], but none specifically assessed this using standard diagnostic criteria—progressively motile sperm <32% or total motile sperm <40%. In our experimental model, we evaluated normoxic (N10W) and hypoxic (H10W) rat cohorts under simulated high‐altitude conditions and found no cases of asthenospermia in either group, as both exhibited sperm motility parameters above these thresholds. These findings suggest that chronic hypoxia alone may not be sufficient to induce clinically diagnosable asthenospermia, indicating that additional environmental or genetic factors may be necessary for its manifestation; further large‐scale epidemiological studies are needed to clarify the relationship between altitude‐related hypoxia and male fertility outcomes.

Under normal physiological conditions, the oxygen tension in the testis is maintained at approximately 2% O_2_ (12–15 mmHg), which plays a critical role in spermatogenesis [41]. However, pathological conditions such as varicocele or testicular torsion can lead to a reduced blood supply to the testis, thereby decreasing testicular oxygen tension below 2% O_2_ and inducing hypoxic conditions that disrupt sperm development. Consequently, our in vitro model at 1% O_2_ was designed to replicate these clinically relevant hypoxic conditions. In addition, high‐altitude hypoxia at 5800 m corresponds to approximately 11.7% oxygen tension. As highlighted by John West in “High Altitude Medicine and Physiology” prolonged exposure to such hypoxic conditions induces physiological adaptations in model organisms (e.g., rodents), manifesting as reduced sperm motility, increased DNA damage, and testicular oxidative stress [42]. These phenotypes are clinically relevant to teratozoospermia. Consequently, we employed a simulated altitude of 5800 m (equivalent to 11.7% O_2_ tension) for the in vivo experiments conducted in this study.

The harmful effects of hypoxia on spermatogenesis are widely recognized, and many studies have investigated the underlying mechanisms, including excessive ROS‐mediated oxidative stress, HIF‐1α‐mediated germ cell apoptosis, inhibition of proliferation, systematic inflammation and epigenetic changes [43]. To perform in‐depth and systemic investigations of spermatogenesis, several studies have investigated spermatogenesis in different models using scRNA‐seq [44]. Our study, involving scRNA‐seq and subsequent in‐depth bioinformatics analysis, revealed that hypoxia exposure led to reduced production of elongated spermatids but an increased yield of round spermatids. These findings suggest that an obstacle to this important step in the spermatid development process may be one of the main factors responsible for the marked decrease in mature sperm under exposure to chronic severe hypoxia.

The transformation of round spermatids into elongated spermatids involves a cascade of morphological and biochemical changes, encompassing the transition from histones to protamines, nuclear condensation, cytoplasmic extrusion, and the development of the acrosome and cilium. Throughout this process, microtubules are instrumental in the transport of cellular organelles, cell division, and the formation of cilia. Conversely, microtubule instability can result in incomplete axonemal structures, leading to defects in the morphology and motility of spermatozoa tails, which in turn can impact male fertility [45]. Our study indicates that chronic hypoxia can lead to a reduction in microtubule stability in the axonemes of rat spermatozoa cilia and in murine spermatogenic cell lines. Specifically, we observed a decrease in microtubule stability in G1 cells and a concurrent reduction in both assembly and stability in G2 cells. Additionally, hypoxia is known to modulate microtubule stability through the regulation of GTP, ATP, and calcium ions, which are crucial for cellular respiration and signaling [46]. Furthermore, we detected a decrease in the intracellular GTP, ATP, and calcium ion levels in both spermatogenic cells and spermatozoa, suggesting that hypoxia‐induced microtubule destabilization is the result of a complex interplay of factors.

The transition zone (TZ), situated between the basal body and the cilium, acts as a diffusion barrier or “molecular gate,” primarily preventing the nonspecific entry and exit of ciliary membrane proteins, thereby maintaining their homeostasis. The pore complex of the TZ regulates the diffusion of cytoplasmic proteins in a size‐dependent manner, facilitating the selective transport of larger molecules and complexes [47]. Ben Chih et al. demonstrated that disruption of the B9D1–TMEM231 complex, a key constituent of the transition zone protein complex, leads to a heightened rate of diffusion into the ciliary membrane and an increased presence of plasma membrane proteins within the cilia [48]. Ruxandra Bachmann‐Gagescu demonstrated that the transition zone protein CC2D2A interacts with NINL, playing a pivotal role in vesicle trafficking, which is regulated by the RAB8‐MICAL3 axis [49].

CEP162 has been shown to interact with core TZ components and facilitate their association with microtubules. Notably, overexpression of CEP162 has been reported to result in its ectopic localization within the distal cilia of retinal pigment epithelial cells, subsequently leading to the formation of abnormally elongated cilia with swollen tips [13]. In the context of our study, we hypothesized that hypoxic conditions upregulate the expression of the centrosomal protein CEP162. This increase in CEP162 expression has been proposed to recruit additional TZ proteins to the basal body, leading to TZ assembly. Consequently, this assembly is thought to reduce the permeability of the TZ and result in an aberrant distribution of soluble proteins within the cilium, potentially impacting ciliary function and morphology. Importantly, our study revealed that chronic hypoxia upregulated the expression of the centrosomal protein CEP162 and the transition zone proteins RPGRIP1L, TRAF3IP1, TMEM67, and CIBAR1 in rat sperm. Additionally, we observed that overexpression of CEP162 in mouse spermatozoa led to a diminished distribution of TUBB3 within the cilia. CLASP2 has been reported to use its C‐terminal TOG4 domain to recognize and bind proteins that contain the TOG4 binding motif (TBM), which is essential for the attachment of condensates such as CLIP170 and ELSK1 to CLASP2. Within these condensates, the association of phosphorylated CLASP2 with ELSK1 condensates is increased, whereas its interaction with CLIP170 condensates is reduced. This competition mediates the attachment of their respective condensates and promotes the targeted positioning of microtubules to specific cellular regions [50]. Interestingly, TUBB3, recognized for its role as a GTP‐binding protein, can be phosphorylated, which plays a pivotal role in regulating its interactions with other protein condensates, notably CEP162 and TUBA3A. The phosphorylation state of TUBB3 can modulate the affinity and specificity of these interactions, thereby influencing the structural integrity and functional dynamics of the protein complexes involved. This phosphorylation‐dependent regulation is crucial for the orchestration of cellular processes that hinge on the precise assembly and disassembly of microtubule‐associated protein networks. CEP162 engages in competitive binding with TUBA3A for TUBB3, potentially explaining, at least partially, the observed decrease in TUBB3 distribution and the associated destabilization of the axonemal microtubules within the cilia. Further analysis of the shared binding site or motif between the proteins CEP162 and TUBA3A and the protein TUBB3 is needed, particularly the use of BLI to assess the affinities between individually purified truncated proteins.

Additional sex comb‐like (ASXL) proteins are mammalian homologs of the addition of sex combs (Asx), proteins that regulate the balance of trithorax and polycomb functions in Drosophila. All three ASXL family members (ASXL1, ASXL2, and ASXL3) are involved in cancer or rare developmental syndromes [28]. ASXL1, ASXL2, and ASXL3 share approximately 70% amino acid homology in the conserved domains of the proteins. ASXL1 and ASXL2 are widely expressed in mammalian tissues, while ASXL3 expression is mostly restricted to the brain and eye [28]. In addition, ASXL1 and ASXL2 have been proposed to physically interact. ASXL1 loss has been repeatedly associated with the global loss of histone H3 lysine 27 trimethylation (H3K27me3), which is uniquely performed by the PRC2 complex, which includes the EZH2, SUZ12 and EED subunits [31]. Therefore, ASXL2 is potentially associated with the function of PRC2, a relationship that has not yet been widely reported. Our data indicate that hypoxia significantly downregulates the expression of ASXL2 and subunits of the PRC2 complex, including EZH2, SUZ12, and EED.

Moreover, ASXL2 directly interacts with EZH2, SUZ12, and EED, as confirmed by coimmunoprecipitation (co‐IP), immunofluorescence, CUT&Tag sequencing, and biolayer interferometry assays. Owing to the transcriptional repression function of ASXL2/PRC2, the hypoxia‐induced decrease in ASXL2/PRC2 likely mediates the expression of a series of genes that are normally downregulated as a prerequisite for the round–elongating spermatid transition [44, 51]. Similarly, in mice with conditional knockout of ASXL2 and EZH2, specifically in spermatogenic cells, we observed an increase in the transcription of CEP162. CUT&Tag sequencing and EMSAs revealed that ASXL2, EZH2, and H3K27me3 all bind to the promoter region of the CEP162‐encoding gene, particularly within the 3482–3511 bp fragment, where hypoxia reduces the binding of ASXL2, EZH2, and H3K27me3 to this region.

ASXL2 is known for its role in recognizing histone modifications through its plant homeodomain (PHD) and for mediating protein‒protein interactions via its ASXH, ASXM1, and ASXM2 domains. These interactions are crucial for the recruitment of epigenetic regulators and transcription factors, such as BAP1, KDM1A, NCOA1, and nuclear hormone receptors (NHRs), to specific genomic loci [15]. The absence of ASXL2 results in significant alterations in histone enhancer marks, including H3K27ac, H3K4me1, and H3K4me2, which are closely associated with changes in gene expression levels. Furthermore, ASXL2 interacts with the histone deacetylases HDAC1 and HDAC2, potentially modulating H3K27 acetylation levels by recruiting or stabilizing the HDAC complex [15]. Interestingly, our results indicate that ASXL2 can recruit EZH2 to catalyze the formation of H3K27me3 modifications on oligonucleosomes, adding another layer to the complex interplay between ASXL2 and the epigenetic machinery. These findings suggest that ASXL2 plays a multifaceted role in the regulation of gene expression through its interactions with various components of the epigenetic landscape.

Building upon these molecular insights into ASXL2‒PRC2 interactions, we used severe hypoxia (1% O_2_) in our in vitro experiments to elicit detectable molecular responses. While this acute hypoxic exposure may amplify microtubule destabilization compared with the chronic moderate hypoxia (11.7% O_2_) observed in vivo, dose‒response analyses (Supplementary Figure S4b A–C) confirmed that both conditions similarly impair the ASXL2‒EZH2 axis. This functional conservation across hypoxic intensities supports the mechanistic relevance of our findings. Future studies using physiological hypoxia models (e.g., intermittent hypoxia or 5–10% O_2_) will further refine these insights while preserving translational relevance to human pathological contexts.

Complementary to these epigenetic perturbations, mitochondrial dysfunction—a hallmark of hypoxic stress—may synergistically exacerbate microtubule destabilization through ATP depletion. Our observed hypoxia‐induced reductions in ATP and GTP levels (Supplementary Figure S2A–D) align with established links between mitochondrial respiratory chain impairment and axonemal defects in asthenozoospermia [52]. Specifically, hypoxia may disrupt mitochondrial crista architecture in spermatids, compromising oxidative phosphorylation and intraflagellar transport energetics [53]. Notably, reactive oxygen species (ROS) overproduction under hypoxia can oxidize tubulin dimers, directly destabilizing microtubules—a mechanism potentially intersecting with ASXL2‐EZH2 axis dysfunction via redox‐sensitive histone modifications [54]. This finding aligns with clinical observations in varicocele patients, who exhibit elevated testicular ROS levels and abnormal histone retention in sperm, paralleling the oxidative stress and chromatin defects observed in our hypoxia models [55]. Wang et al. reported that sperm abnormalities (e.g., head defects and reduced motility) are correlated with both altitude and varicocele severity, mirroring the hypoxia‐induced microtubule destabilization reported in our study [56]. While our study focused on epigenetic regulation, future work should explore the crosstalk between mitochondrial homeostasis and the ASXL2‐EZH2 axis, particularly in metabolic reprogramming during spermiogenesis.

Through our thorough review of the MIK literature database, we delineated the collaborative role of ASXL2 and EZH2 in male infertility. Our analysis, which utilized RNA transcriptome and PCR array data from the GEO public database for teratozoospermia and nonobstructive azoospermia, revealed a significant downregulation of the transcriptional levels of PRC components, notably ASXL2 and EZH2, in these specific infertility conditions. These findings underscore the potential significance of these genes in the pathogenesis of spermatozoa abnormalities and spermatogenic failure.

## 4 Conclusions

Our comprehensive study revealed that chronic hypoxia hinders spermatogenesis by impeding the transition of spermatids from round to elongated forms. Our findings establish that hypoxia disrupts spermatogenesis via: (i) ATP depletion‐driven microtubule instability, and (ii) ASXL2‐EZH2‐mediated epigenetic dysregulation, which leads to CEP162‐induced TUBB3 mislocalization. Specifically, this transition is controlled by the ASXL2–EZH2 axis. Under hypoxic conditions, ASXL2 is downregulated, which reduces EZH2 binding to the 3482–3511 bp region of the CEP162 promoter. This leads to a decrease in H3K27me3 modification and an increase in CEP162 transcription. Elevated CEP162 then competes with TUBA3A for binding to TUBB3, causing a reduction in TUBB3 levels within cilia and contributing to microtubule destabilization, which is associated with sperm morphological and functional defects. Clinical observations of reduced ASXL2 and EZH2 in men with infertility underscore the ASXL2–EZH2 axis as a significant target for therapeutic intervention in hypoxia‐related spermatogenesis disorders.

## 5 Materials and Methods

### 5.1 Research Ethics

This study was conducted in accordance with the principles of the Declaration of Helsinki and was approved by the Medical Ethics Committee of the Second Affiliated Hospital of Army Medical University, PLA (Approval Number: 2025‐135‐01). Written informed consent was obtained from all participants prior to their inclusion in the study. The animal experiments in this study were approved by the Army Medical University Institutional Animal Care and Use Committee (AMUWEC20230266) and were conducted following the National Institutes of Health Guide for the Care and Use of Laboratory Animals (NIH Publications No. 8023, revised in 1978).

For the collection of semen samples, we included 6 patients with teratozoospermia and 6 normozoospermic controls. All semen analyses were performed according to the World Health Organization (WHO) guidelines.

With respect to the testicular biopsy samples, 1 patient with nonobstructive azoospermia was recruited, and biopsies were performed to assess spermatogenesis. Additionally, 1 normozoospermic testicular biopsy sample was obtained from a patient who underwent surgical procedures for clinical indications such as testicular torsion. These normozoospermic individuals had normal semen parameters as defined by the WHO criteria. Importantly, normozoospermic testicular biopsies were obtained only from patients who underwent clinically indicated surgeries, as it is not ethically feasible to perform biopsies on healthy individuals for research purposes alone.

### 5.2 Rats and Mice

The rats subjected to hypoxic conditions were raised in a hypobaric oxygen chamber, where the atmospheric pressure was reduced to simulate an altitude of 5800 m. The partial pressure of oxygen decreased as the total pressure decreased during ascent, but the oxygen percentage in the atmosphere did not change. The control rats were raised at an altitude of 300 m outside the hypobaric oxygen chamber. All the animals were maintained until 8 weeks of age before hypoxia exposure, and these rats had free access to standard pellet food and water. The animals were maintained under controlled lighting conditions (12 h light:12 h darkness), the ambient temperature was maintained at 22°C–24°C, and the relative humidity was maintained at 40–60%.

Spermatogenic cell‐specific conditional ASXL2/EZH2‐knockout (KO) mice (project no: CKOAI211124LL1‐B‐UP and CKOAI211124LL2‐B‐UP) were generated by Cyagen Biotechnology Co., Ltd., Guangzhou, China. Briefly, a tamoxifen‐inducible Cre system was used to generate ASXL2‐cKO C57BL/6J mice (Stra8‐iCreERT2; ASXL2^lox/lox^) and EZH2‐cKO mice (Stra8‐iCreERT2; EZH2lox/lox) by crossing Stra8‐iCreERT2 mice with ASXL2^lox/lox^ or EZH2^lox/lox^ mice, which possess loxP sites flanking exon 5. The genotypes of the offspring were determined via PCR.

### 5.3 Cell Culture, Hypoxia Treatment and Transfection

The mouse spermatogonium‐derived cell line GC‐1 and mouse pachytene spermatocyte‐derived cell line GC‐2 were obtained from Procell Ltd. (Wuhan, China) and cultured in DMEM supplemented with 10% fetal bovine serum. Hypoxia (1% O_2_) was achieved by mixing high‐purity gases (5% CO_2_, 10% air and 85% N_2_) in a 37°C hypoxia workstation (Invivo2, Baker Ruskinn, Sanford, ME, USA). All experimental procedures, including protein isolation and RNA isolation, were performed on a hypoxia workstation.

A hypoxic model of GC‐1/2 cells was established under 48 h of hypoxia exposure. A total of 5 × 10^5^ cells/well were cultured in six‐well dishes (diameter 35 mm) at 37°C in a 21% O_2_ and 5% CO_2_ incubator. The cells were washed once with PBS after 24 h, after which they were cultured at 37°C in a 1% O_2_ hypoxia workstation for 48 h. Hypoxia at 1% O_2_ was selected for in vitro experiments on the basis of pilot studies demonstrating its efficacy in inducing hypoxia‐responsive pathways (e.g., HIF‐1α stabilization and ASXL2 downregulation) within a 48‐h timeframe, which is consistent with the transcriptional changes observed under chronic moderate hypoxia in vivo.

For cell line experiments, the hypoxic condition was standardized at 1% O_2_, in accordance with prior studies and widely accepted as a representative model of hypoxia in cellular research [57, 58]. This level of severe hypoxia was chosen to simulate the extreme oxygen‐deprived conditions observed in clinical scenarios such as varicocele or testicular torsion, where tissue oxygen levels can fall substantially below 2% [41]. Such a low‐oxygen environment is critical for investigating the impact of acute hypoxia on spermatogenesis and overall testicular function.

The following small interfering RNA (siRNA) sequence was used to inhibit mouse ASXL2/EZH2 expression: ASXL2 (*GCCCAAAGCAGGUUCUAAUTT* (sense)) and *AUUAGAACCUGCUUUGGGCTT* (antisense)). EZH2 (GCACAAGUCAUCCCGUUAATT (sense) and UUAACGGGAUGACUUGUGCTT (antisense)). The siRNA sequences for the negative controls were as follows: *UUCUCCGAACGUGUCACGUTT* (sense) and *ACGUGACACGUUCGGAGAATT* (antisense). Transient transfection experiments were conducted using Lipofectamine 3000 (Invitrogen) following the manufacturer's protocols. All experiments adhered to the following replication strategy: Figure 4E,F: twelve biological replicates; Figure 5H–K and Supplementary Figure S6A–D: ten biological replicates.

To activate ASXL2 in mice, AAV2‐EFs‐ASXL2‐3xFlag‐pA was used to overexpress mouse ASXL2 (NM_001270988). ASXL2 was synthesized from nucleic acid sequences obtained from the NCBI database and cloned and inserted into an AAV vector. To package ASXL2 tagged with Flag (approximately 4.3 kb) under the control of a ubiquitous promoter into AAV vectors, we utilized the pAAV2‐EFS‐pA vector (Taitool Bioscience, Shanghai, China), which has a short form of the constitutively acting elongation factor 1α (EFS) promoter (approximately 0.25 kb) and a minimal synthetic polyadenylation signal (48 bases). The final recombinant mASXL2‐overexpression expression cassette was designated pAAV2‐EFS‐mASXL2‐3xflag‐pA. The viral particles were packaged into AAV9 vectors and purified at titers ≥ 2.2E+13 V.G./ml by Taitool Bioscience Co.

To activate EZH2 in mice, an AAV vector plasmid (pAAV2‐CAG‐EZH2‐3xFlag‐WPRE‐pA) was used to overexpress mouse EZH2 (NM_007971.2). EZH2 was synthesized on the basis of nucleic acid sequences obtained from the NCBI database and cloned and inserted into an AAV vector with a CAG promoter. The AAV vector was generated by transfecting three plasmids (pAAV2‐CAG‐EZH2‐3xFlag‐WPRE‐pA containing the EZH2 gene flanked by the AAV inverted terminal repeat sequences, the pAAV9 trans‐plasmid with the AAV rep and cap genes, and the pAAV helper plasmid) into HEK293T cells. Viral particles were harvested at 3 days post‐transfection and purified via iodixanol gradient ultracentrifugation. The titers of the vector genome were measured via qPCR with vector‐specific primers.

To overexpress ASXL2/EZH2 in G2 cells, the target gene ASXL2 was inserted into the vector pGC‐FU‐3FLAG‐CBh‐IRES‐puromycin. The target gene EZH2 was inserted into the vector pGC‐FU‐3FLAG‐CBh‐IRES‐puromycin. The packaging plasmid mixture and the target gene adenoviral vector plasmid were cotransfected into the packaging cells of the 293T cell line. After transfection for 48–72 h, the cell supernatant was collected, and the collected supernatant was concentrated and purified to improve the titer and purity of the virus. Finally, a titer ≥ 1E+8 TU was detected via a fluorescence counting method.

We overexpressed TUBA1A in cells using a lentivirus carrying the TUBA1A‐GFP sequence. The lentiviral construct was constructed from the pHBLV‐CMV‐TUBA1A‐GFP vector from Hanbio, and the virus was produced by cotransfecting 293T cells with the PSPAX2 and PMD2G plasmids using LipoFiter. The virus‐containing supernatant was collected 48 h post‐transfection, filtered, and concentrated via ultracentrifugation.

For in vivo overexpression of CEP162, we engineered a lentiviral vector by integrating the CEP162 coding sequence downstream of a potent promoter in the pLVX vector. The recombinant lentivirus was produced by cotransfecting 293T cells with the constructed vector and packaging plasmids, followed by virus harvesting, concentration, and purification to yield high‐titer viral stocks for in vivo transduction.

### 5.4 Prokaryotic Expression and Purification

The genes encoding ASXL2 (4122 bp), EZH2 (2250 bp), SUZ12 (2235 bp), and EED (1335 bp) were amplified using PCR with specific primers containing the NdeI and BamHI restriction sites. The amplified products were digested with NdeI and BamHI and ligated into the pET‐28a(+) expression vector, which was subsequently transformed into chemically competent *Escherichia coli* cells for propagation. The transformed *E. coli* cells harboring the recombinant plasmids were cultured in LB medium supplemented with kanamycin (15 µg/mL) at 37°C until an OD600 of 0.6–0.8 was reached. Protein expression was induced with 1 mM isopropyl β‐D‐1‐thiogalactopyranoside (IPTG), and the cultures were further incubated at 18°C for 16 h to allow the accumulation of the recombinant proteins, which were fused with a His‐tag for purification purposes. The cells were harvested by centrifugation at 4000 × g for 20 min at 4°C and resuspended in lysis buffer (50 mM NaH_2_PO_4_, 300 mM NaCl, and 10 mM imidazole, pH 8.0) containing a protease inhibitor cocktail and 1 mg/mL lysozyme. The cells were lysed by incubation on ice for 30 min, followed by sonication to ensure complete disruption. The cleared lysate was obtained by centrifugation at 12 000 × g for 30 min at 4°C. The supernatant was then incubated with Ni‐NTA agarose beads for 1 h at 4°C with gentle rotation to bind the His‐tagged proteins. The beads were washed with wash buffer (50 mM NaH_2_PO_4_, 300 mM NaCl, 20 mM imidazole, pH 8.0) to remove unbound proteins. The target proteins were eluted with elution buffer (50 mM NaH_2_PO_4_, 300 mM NaCl, 250 mM imidazole, pH 8.0). The expression and purity of the recombinant proteins were analyzed by SDS‒PAGE. The protein bands were visualized by Coomassie Brilliant Blue staining. The molecular weights of the expressed proteins were compared to the expected sizes on the basis of the gene sequences.

### 5.5 Gene Expression Dataset Analysis

To determine the expression levels of ASXL2, EZH2, EED, YY1, BRD1, WDR5, RBBP4, RNF2 and USP7 and the expression of their target genes in patients with teratozoospermia, normalized mRNA expression data derived from BioProject “PRJNA104107” of the NCBI platform, were obtained from the Gene Expression Omnibus (https://www.ncbi.nlm.nih.gov/geo/query/acc.cgi?acc=GSE6872). To determine the expression levels of the above target genes in idiopathic NOA patients, normalized mRNA expression data derived from BioProject “PRJNA788390” were obtained from the Gene Expression Omnibus (https://www.ncbi.nlm.nih.gov/geo/query/acc.cgiacc=GSE190752).

The RNA abundance profiles of normospermic individuals and teratozoospermic individuals were downloaded from the NCBI GEO dataset GSE6872. The RNA abundance profiles of healthy individuals and idiopathic NOA individuals were downloaded from the NCBI GEO dataset GSE190752. Gene expression analysis was performed following the author's instructions. Specifically, the data were analyzed with DChip using invariant set normalization and PM‐only MBEI model settings (GSE6872), or the raw expression data were summarized after background correction and normalization steps using the rma methodology in the affy package of the Bioconductor project (GSE190752). Gene expression signal intensity was used to compare multiple samples from different groups. To stratify samples into high or low groups, the mean intensity was calculated across all samples, and the expression levels were categorized as “high‐level” (i.e., above the mean value) or “low‐level” (i.e., below the mean value).

### 5.6 Cell Grading Separation of Free Tubulin and Polymerized Tubulin

The cells (from 1 mL of cell suspension) were collected by centrifugation at 6000 × g for 1 min at room temperature. The cell precipitate was resuspended in 25 mL of TMMET buffer (20 mM Tris HCl, pH 6.8, 0.14 M NaCl, 1 mM MgCl_2_, 2 mM EGTA, 4 mg/ml paclitaxel, 0.5% NP40) for 5 min. After centrifugation at 14 000 × g for 10 min at room temperature, equal proportions of the supernatant (free microtubule protein) and precipitate (polymerized microtubule protein) were transferred to 5 × SDS loading buffer, and SDS‒PAGE and Western blot analysis were performed.

### 5.7 Microtubule Assembly

For microtubule polymerization by Taxol, short microtubule seeds were prepared by incubating a 32 µM porcine brain tubulin mixture containing 10% TAMRA‐labeled tubulin with 20 µM Taxol, 4 mM MgCl_2_ and 4% DMSO. After incubation on ice for 5 min, the mixture was polymerized in a 37°C water bath for more than 30 min in the dark. The reaction was terminated by adding 400 µL of BRB80 buffer (preequilibrated to 37.0 ± 0.5°C) supplemented with 20 µM Taxol and a gradient of GTP concentrations (0.25, 0.5, 0.75, 1.0, and 1.25 mM), followed by immediate processing for confocal microscopy using an Olympus IX81 system (Olympus Corporation, Tokyo, Japan).

### 5.8 Tubulin Turbidity Assay

Microtubule polymerization or depolymerization was monitored by measuring the changes in absorbance (340 nm) via a microplate reader. In brief, all of the components of the reactions in the 96‐well plates were incubated at 37°C for 1 min. After gentle mixing, the reaction mixtures were immediately analyzed via a microplate reader at 37°C.

### 5.9 Microtubule Regrowth Assay

The cells were grown on pre‐sterilized 8‐mm‐diameter glass coverslips (Thermo Fisher Scientific) in a 3.5‐cm tissue culture dish. Coverslips for individual timepoints were taken from the same culture dish. One coverslip for each experiment was fixed in fixation buffer (described above) prior to nocodazole treatment to confirm the phenotype of the cell sample. To minimize the loss of mitotic cells, the coverslips were transferred to new dishes containing the indicated buffers. First, the coverslips were incubated with 10 µM nocodazole diluted in DMEM/F12 + 10% FBS or DMEM/F12 + 5% HS + 2.5% FBS for 1 h at 37°C. Then, the coverslips containing ARPE‐19 cells were transferred to DMEM + 10% FBS for 5 min, and the coverslips containing IMCD3 cells were transferred to DMEM + 5% HS + 2.5% FBS for 10 min, after which the coverslips were incubated at 37°C, followed by fixation at the indicated time points. One coverslip for each experiment was fixed. The cells were processed for immunofluorescence as described below.

### 5.10 Cell Viability Assay

Cell proliferation was determined using the WST‐8 tetrazolium salt assay (Cell Counting Kit‐8, Beyotime). The cells were cultured in 96‐well plates at a density of 2 × 10^4^/well in 0.1 mL of culture medium before hypoxic treatment. 1 h before the indicated incubation period, 10 µL of WST‐8 reagent was added to the cells. At the end of the incubation, the cell density was estimated by measuring the absorbance of the colored formazan reaction product at 450 nm using a Multiskan Go Microplate Absorbance Reader (Thermo, Massachusetts, USA). Cell viability was assessed by calculating the ratio of the absorbance of the treatment group to that of the control group.

### 5.11 Sperm Motility, Count and Motion Parameter Analyses Using a Sperm Class Analyzer (SCA)

All protocols were approved by the Army Medical University Institutional Animal Care and Use Committee and were conducted following the National Institutes of Health Guide for the Care and Use of Laboratory Animals (NIH Publications No. 8023, revised in 1978). Hypoxic mice were raised in a hypobaric chamber, where the atmospheric pressure was reduced to simulate an altitude of 5800 m. The partial pressure of nitrogen decreased as the total pressure decreased during ascent, but the nitrogen percentage in the atmosphere did not change. The control mice were raised at an altitude of 300 m outside the hypobaric chamber. All the animals were maintained until 10 weeks of age before hypoxia exposure, and these mice had free access to standard pellet food and water. The animals were maintained under controlled lighting conditions (12 h light:12 h darkness), the ambient temperature was maintained at 22°C–24°C, and the relative humidity was maintained at 40–60%.

The sperm motility, count, and other motion parameters, including the curvilinear velocity, straight‐line velocity, average path velocity, straightness, beat‐cross frequency and amplitude of lateral head displacement, were analyzed by a sperm class analyzer (SCA). The mice were anesthetized via inhalation of 4% isoflurane (TargetMol, CAS T19651) in 100% oxygen using a calibrated vaporizer (Harvard Apparatus Model 722). Anesthesia was maintained at 1.5–2% isoflurane delivered through a nose cone, with the respiratory rate (60–80 breaths/min) and pedal reflex monitored every 10 min to ensure adequate depth. The mice were sacrificed by an overdose of anesthesia. The epididymides were removed and weighed. The epididymides were subsequently divided into three parts: head, body and cauda. The sperm in the epididymal cauda were analyzed for their count, motility and viability. In brief, the caudal epididymides were isolated and minced. The caudal tissue was placed in preheated (at 37°C) F12 with 0.5% BSA and liquefied for 10 min. An aliquot (0.05 mL) of the resulting sperm suspension (1 mL) was diluted with 1:50 PBS (pH 7.2) and mixed thoroughly. An aliquot of the diluted mixture (50 µL) was subsequently analyzed for sperm motility and concentration using an SCA (MICROPTIC S.L., Barcelona, Spain).

### 5.12 Sperm Penetration Assay

The epididymides were removed and weighed. The sperm suspensions were prepared by mincing cauda in PBS containing 200 µg/ml trypsin and 400 U/ml DNase I and centrifuging at 35°C and 215 × g for 4 min, after which FBS was added to terminate the digestion. The erythrocytes were centrifuged and lysed with erythrocyte lysing reagent (C3702, Beyotime, Shanghai, China). The suspension was filtered through 40‐µm mesh to remove tissue fragments. The filtered cell suspension was collected and centrifuged (600 × g for 5 min). The isolated sperm were placed into F12 media (GF3105, Genview, Shanghai, China) supplemented with 0.5% BSA at 37°C for 10 min.

10‐week‐old females were superovulated via the intraperitoneal injection of 10 IU of pregnant mare serum gonadotropin (G4877, Sigma), followed by the injection of 10 IU of human chorionic gonadotropin (CG10, Sigma) 48 h later. Oocytes were collected from oviducts into M‐2 medium (MR‐015‐D, Millipore) 20 h after hCG injection, and cumulus oophorus cells were removed by treatment with 0.1% hyaluronidase in M‐2. The cumulus‐free oocytes were subsequently washed in M‐2 medium.

The sperm suspensions were cultured in BWW medium for 1 h at 37°C with 21% O2 and 5% CO2. Then, the superovulated mouse oocytes were transferred to sperm suspensions at a ratio of 3.5 × 10^6^ sperm per oocyte. The amount of spermatogonia that entered the oocyte was determined via acetic acid magenta staining, and the percentage of sperm that penetrated the oocyte was calculated.

### 5.13 Measurement of Serum Hormone Levels

Blood was collected, coagulated for 2 h and then centrifuged at 4°C and 4000 rpm for 15 min to separate the serum. Then, the serum hormone (GnRH, LH, FSH, ABP, and T) levels were measured using commercial ELISA kits (Meimian Industry Co., Ltd., Jiangsu, China) according to the manufacturer's instructions. Other serum samples were stored at −80°C.

### 5.14 Assessment of Epididymal Sperm Morphology

For transmission‐mode scanning electron microscopy (TSEM) analysis, the sperm samples were subjected to optimized chemical fixation with 2.5% glutaraldehyde (0.1 M cacodylate buffer, pH 7.4 ± 0.1, osmolarity of 320 ± 5 mOsm/kg), followed by post‐fixation with 1% osmium tetroxide containing 1.5% potassium ferrocyanide. Critical point drying was performed using a Leica EM CPD300 system with dry ice‐cooled liquid CO_2_ (triple purge cycle, 31.1°C critical temperature). TSEM imaging was conducted on a ZEISS CROSSBEAM 340 system equipped with a STEM3 detector (30 µm aperture, 5 kV accelerating voltage, 50 pA probe current), with instrument parameters calibrated against NIST SRM 2066 resolution standards.

### 5.15 Adhesion Force Measurements by AFM

AFM force measurements were performed using an Asylum MFP‐3D AFM system, which was integrated on top of an Olympus IX51 inverted optical microscope. This setup allows visualization of the sample and manual positioning of the regions to be probed with precision. The force measurements, specifically force‒distance curves, were executed at a frequency range between 0.3 and 2.0 Hz to ensure accurate data acquisition. For these measurements, we used Si3N4 or Si cantilevers that had been modified with various types of spherical tips to suit the specific requirements of the samples being analyzed. The tips used included a glass sphere with a radius of 10 µm, a glass sphere coated with gold layers of the same radius, and a polystyrene sphere with a slightly larger radius of 12.5 µm, all obtained from Novascan. These cantilevers were calibrated to have spring constants within the range between 0.6 and 14 N/m, which is crucial for accurate force measurements.

The sample modulus, denoted as E_sample_, reflects the stiffness of the sample material, which is the ability of the sample to resist deformation when subjected to an external force. The higher the sample modulus is, the harder or less deformable the sample is. The reduced modulus, denoted as E_0_, is used to describe the average elastic properties of the probe and the sample when they are in contact. The higher the reduced modulus is, the greater the average elasticity of the contact area between the probe and the sample, meaning that it is harder or less prone to deformation.

### 5.16 Periodic Acid‐Schiff (PAS) Staining of Glycogen

The animals were euthanized via decapitation. The right testis was excised, fixed in 10% formalin, dehydrated, and embedded in paraffin. Sections were cut to 5 µm thickness and stained with PAS for light microscopic observation.

### 5.17 Papanicolaou Staining

The dried sperm smears were fixed in 95% alcohol for 15 min. Then, the smears were immersed in 75% ethanol, 50% ethanol, or water for 30 s. Next, the smears were stained with hematoxylin staining solution (Harris) for 5 min and washed with water for 1 min. Subsequently, the smears were differentiated in 1% hydrochloric acid alcohol solution for several seconds and returned to blue in running water for 10 min. Then, the smears were immersed in 50% ethanol, 75% ethanol, and 95% ethanol for 30 s, 30 s, and 2 min, respectively. Next, the smears were stained with orange G staining solution for 1 min and immersed in 95% ethanol twice for 15 s each time. Afterward, the smears were stained with EA36 staining solution (bright green and eosin) for 5 min. Finally, the smears were immersed in 95% ethanol twice for 15 s and anhydrous ethanol twice for 45 s each.

### 5.18 10× Genomics Single‐Cell RNA‐Seq Preprocessing

Sequencing was performed by Shanghai Liebing Biomedical Technology Co., Ltd. Reference sequences and gene annotations from the reference rat genome rn6 were downloaded from UCSC and then converted to Cell Ranger (v3.1.0) reference data via the ‘cellranger mkref’ command. The raw reads were processed by “cellranger count.” The identified cells and genes were further filtered. Only cells in which at least 500 expressed genes were detected were retained, and only genes expressed in more than 10 cells were retained. The expression levels of each cell were then normalized by dividing by a scaling factor, which equals the ratio of that cell's total reads to the median total reads of all cells. Finally, the matrix was transformed with log2(x+1), where x is the individual expression value. The raw data were deposited to the National Center of Biotechnology Information (NCBI) database with the accession number BioProject PRJNA926018, SRA accession number SRP421799, and experiments numbers SRR23389463‐ SRR23389468.

### 5.19 Doublet Detection and Removal

To minimize the impact of artificial doublets—arising from the co‐encapsulation of multiple cells during droplet‐based single‐cell library preparation—we computationally identified and removed putative doublets using DoubletFinder (v2.0.3). Following quality control and initial preprocessing (including normalization, scaling, principal component analysis, and clustering), we estimated the expected number of doublets based on the cell recovery rate and a standard doublet formation rate of 8 × 10^−6^ per cell. Sample‐specific optimal parameters were determined through a parameter sweep and BCmetric evaluation, with a fixed neighbor proportion (pN) of 0.25 and an optimized pK value selected for each dataset (ranging from 0.005 to 0.28). Doublets were predicted using the DoubletFinder function with the first 30 principal components. Cells classified as “Doublet” were excluded from all downstream analyses, including clustering, trajectory inference, and differential gene expression.

### 5.20 Dimensional Reduction and Visualization With UMAP

The first two reduced components from the expression matrix were processed by UMAP (https://github.com/lmcinnes/umap) (n_neighbors = 100, n_components = 2, min_dist = 0, init = “random,” metric = “Euclidean,” random_state = 123, n_epoch = 1000) and are shown as scatter plots in the figures.

### 5.21 Identification of the Cell Type for Each Cell

The marker genes of 11 mouse testis‐specific cell types, namely, innate lymphoid cells, macrophages, endothelial cells, myoid cells, Sertoli cells, Leydig cells, unknown spermatogonial cells, spermatocytes, round spermatids and elongating spermatids, have been previously described [21]. Because of the experimental enrichment of rare cell types such as spermatogonia and Sertoli cells in 35 000 single‐cell studies, these different cell types could be better separated according to the PCA and t‐distributed stochastic neighbor embedding (tSNE) results [21]. The mean expression level of the marker genes for each cell type was calculated for each cell, together with 1000 permutations of shuffled gene expression values for the marker genes to obtain the expected random background. Using the 1000 permutation background values and the signal values of the mean expression levels for each cell type, the false discovery rate (FDR) and enrichment score (ES) could be calculated. ES equals the mean of marker expression divided by the mean of the permutated background. The FDR equals the ratio of permutation values that are higher than the observed value. When more than 20% of the marker genes were expressed, an FDR < 0.001 and an ES ≥ 5 were used as the criteria for potential annotation of a cell type. We also randomly selected 500 genes that did not overlap with any of the marker genes as a negative control. Therefore, if a cell's top enriched putative cell type ES was smaller than its ES for random genes, the cell was assigned as random_others. If more than one potential cell type was assigned to a cell, the cell type with the higher ES value was assigned as the putative cell type. If no cell type satisfied the criteria, the cell was classified as having no type match. After the initial assignment, we further evaluated the distribution of cells among cell types in the global UMAP plot. For example, a majority of putative spermatocyte cells (5827) clustered together, and only 288 cells were distributed separately; therefore, 288 cells were removed before subsequent analysis.

### 5.22 Per‐Cell Attributes

We calculated per‐cell attributes with reference to [21]. chrM% is the ratio of UMIs mapped to 13 mitochondrial genes; a smaller chrM% indicates a healthier cell. chrX% is the ratio of UMIs mapped to 1114 chromosomal X genes, whereas chrY% is the ratio of UMIs mapped to 45 chromosomal Y genes. UMI(log10) shows the log10‐transformed number of UMI reads. The Gini index measures the gene expression inequality of expressed genes and is calculated as $Gini=\frac{1}{n}\;\left(n+1-2\frac{\sum_{i=1}^{n}\left(n+1-i\right)y_{i}}{\sum_{i=1}^{n}y_{i}}\right)$, where *n* is the number of expressed genes and where *y_i_* is the expression value for the ith gene (sorted in ascending order). The diversity values indicate the dissimilarity among cells within the same cell type, measured by the 1‐mean Pearson correlation coefficient.

### 5.23 Cell Type‐Specific Gene Identification

Cell type‐specific genes were obtained by comparing a given cell type with all others using a two‐sided Student test, implemented as scipy.stats.ttest_ind in the SciPy package [59]. The significance criteria were as follows: 1) ≥20% of cells in the given cell type expressed the gene; 2) ≥10% of the genes were detected for the given cell types vs. others; 3) the *p* value < 1e‐10; 4) the log2‐transformed fold change ≥ 2; and 5) the mean value of the gene's expression in the given cell type and others was greater than the median value for all genes.

### 5.24 Functional Enrichment Analysis

The enriched GO terms within the gene lists were obtained using findGO.pl in the HOMER package [60]. Biological process GO terms assigned to fewer than 500 genes but more than 5 overlapping genes from the gene list and with a *p* value ≤1e‐5 were retained.

### 5.25 Spermatocyte and Elongating Spermatid Subtype Identification

We attempted to identify subtypes contained within the major cell types, and only spermatocytes and elongating spermatids could be stably classified. For putative spermatocyte subtypes, the first 2D vectors from UMAP were clustered using HDBSCAN (https://hdbscan.readthedocs.io/en/latest) (min_cluster_size = 100). For putative subtypes of elongating spermatids, the first 2D vectors from PCA of the normalized expression matrix were clustered by HDBSCAN (min_cluster_size = 100).

### 5.26 Identification of DEGs Between Control and Hypoxia‐Treated Cells

Condition‐specific genes were obtained by comparing the control and hypoxia treatments separately for each cell type/subtype using a two‐sided Student's *t*‐test, implemented as scipy.stats.ttest_ind in the SciPy package [59]. The significance criteria were as follows: 1) *p* value < 1e‐10; 2) log2‐transformed fold change ≥1; and 3) the mean value of the gene's expression in a given cell type and others was greater than the median value for all genes.

### 5.27 Assessment and Exclusion of Somatic Cell Contamination

To minimize potential contamination from Sertoli cells during scRNA‐seq sample preparation, fluorescence‐activated cell sorting (FACS) was performed based on DNA content to isolate highly purified populations of diploid and haploid spermatogenic cells. To further differentiate between genuine elongating spermatids and Sertoli cell‐encapsulated elongating spermatid cytoplasm, co‐staining was conducted using SOX9 (a well‐recognized Sertoli cell marker) and PRM2 (an elongating spermatid‐specific nuclear protein) prior to FACS. Flow cytometry analysis demonstrated that the sorted diploid spermatogenic cell population contained no PRM2‐positive cells, thereby excluding contamination from elongating spermatids. The sorted elongating spermatid fraction exhibited 100% PRM2 positivity and lacked detectable SOX9‐positive signals, effectively eliminating Sertoli cell contamination. Furthermore, to computationally identify and remove doublets resulting from incomplete tissue dissociation, the scRNA‐seq dataset was re‐analyzed using Scrublet (v0.2.3). Cells classified as putative doublets—based on transcriptomic similarity to simulated doublets—were excluded from downstream analyses. This integrated experimental and computational approach ensured high‐quality, contamination‐free data for subsequent trajectory inference and clustering analyses.

### 5.28 Isolation of Spermatogenic Cells, Round Spermatids and Elongating Spermatids

Prepubertal 12‐day‐old male mice were anesthetized, and both testes were extracted, decapsulated and digested with 25 µg/ml collagenase IV (Sigma, St. Louis, MO, USA) and 400 U/ml DNase I (Sigma) for 5 min at 35°C in DMEM:F12. The resulting seminiferous tubules were transferred to DMEM:F12 medium containing 200 µg/ml trypsin and 400 U/ml DNase I and digested at 35°C and 215 rpm for 4 min, after which FBS was added to terminate the digestion. The above sample was filtered through a cell sieve, and spermatogenic cells were collected, centrifuged, washed once with FACS buffer, and then passed through the cell sieve again. Cell viability was observed, and the cells were counted via trypan blue staining. Hoechst (Invitrogen, Carlsbad, CA, USA) and dye780 (Invitrogen) were added for staining, and the round spermatids (haploid cells/RS) were separated via flow cytometry.

The cauda epididymis was extracted and liquefied with F12 medium containing 0.5% BSA. The obtained cauda epididymis was subsequently filtered through a cell sieve, and the filtered cell suspension was collected, centrifuged and lysed with erythrocyte lysis buffer. The isolation of spermatogenic cells, round spermatids, and elongated spermatids was performed as follows. 8‐week‐old adult mice were anesthetized, and both testes were extracted, decapsulated and digested with 25 µg/ml collagenase IV (11088858001, Sigma, St. Louis, MO, USA) and 400 U/ml DNase I (AMPD1, Sigma) for 5 min at 35°C in PBS. The resulting seminiferous tubules were transferred to PBS containing 200 µg/ml trypsin and 400 µl/ml DNase I and vortexed at 215 × g at 35°C for 4 min, after which FBS was added to terminate the digestion. The erythrocytes were centrifuged and lysed with erythrocyte lysing reagent (C3702, Beyotime, Shanghai, China). The above system was filtered through a 40 µm cell sieve (F613461, Sangon, Shanghai, China), and the filtered cell suspension was collected, centrifuged (600 × g for 5 min), washed once with FACS buffer, and then passed through the cell sieve again. Single‐cell viability was observed, and the cells were counted by trypan blue staining under a light microscope. Hoechst (Invitrogen, Carlsbad, CA, USA) and dye780 (62910‐00, Invitrogen, CA, USA) were added for staining for 25 min according to 15 µL of Hoechst/2 µL of dye780/4 × 10^7^ cells. The flow cytometric DNA content distribution of various germ cells in the control mice was well characterized by the presence of four main distinct peaks, representing elongating spermatids, round spermatids, diploid spermatogenic cells (including Sertoli cells, spermatogonia and secondary spermatocytes), and tetraploid primary spermatocytes (also known as primary spermatocytes). The round spermatids and elongating spermatids were separated via flow cytometry. Data analysis was performed using BD FACSDiva software. Hoechst was excited using a 375 nm laser, and the wide emission spectrum of the dye was detected in two distinct channels: “Ho Blue” (450/40 nm bandpass filter) and “Ho Red” (670 nm longpass filter). The latter was also used to detect dye780. A dichroic mirror (610 nm longpass filter) was used to split these emission wavelengths. Forward scatter (FSC‐A) and side scatter (SSC‐A) were detected using a 488 nm laser. Two‐way sorting was performed using a seventy‐micron nozzle. The sorting flow rate was adjusted to 2000–3500 events/second. A minimum of 500 000 events were recorded before the gates were set. The cells were sorted into 5 mL polypropylene round‐bottom tubes (by pipetting) containing 1 mL of 5% FBS in Gey's balanced salt solution (GBSS).

### 5.29 Immunofluorescence Staining

Paraffin‐embedded sections were cut into thin slices (4 µm) and deparaffinized. The cells were washed three times with wash buffer (Immunol Fluorescence Staining Kit, Beyotime). Nonspecific binding sites were blocked for 60 min at room temperature with blocking solution. The cells were subsequently incubated with ASXL2 rabbit polyclonal antibody (1:200 dilution, ab106540, Abcam) for 60 min at room temperature. ASXL2 staining was revealed by incubation with Alexa Fluor 488‐conjugated donkey anti‐rabbit IgG (H+L) (1:500 dilution, A21206, Invitrogen) for 60 min at room temperature. Next, the cells were incubated with EZH2 rabbit polyclonal antibody (1:200 dilution, ab283270, Abcam) for 60 min at room temperature. EZH2 staining was revealed by incubation with Alexa Fluor 555‐conjugated donkey anti‐mouse IgG (H+L) (1:500 dilution, A31570, Invitrogen) for 60 min at room temperature. The cell nuclei were stained with DAPI (C1005, Beyotime) for 5 min at room temperature. The cells were subsequently observed under a laser scanning confocal microscope (LSCM, IX81, Olympus, Tokyo, Japan) in random microscopic fields at ×400 magnification.

Furthermore, seminiferous tubule tissues from the azoospermia group and the healthy control group underwent immunofluorescence staining using BRDT (1:500 dilution, ab288435, Abcam). The staining procedure was carried out in accordance with a previously described protocol.

### 5.30 Dual‐Channel Detection of ASXL2 and AURKB in Testicular Torsion: Sequential Immunofluorescence and smFISH in Paraffin Sections

For the simultaneous detection of ASXL2 protein and AURKB mRNA in torsion‐control testicular sections, paraffin‐embedded tissues (4 µm) were sequentially processed by immunofluorescence followed by single‐molecule RNA fluorescence in situ hybridization (smFISH). After deparaffinization in xylene and rehydration through graded ethanols, heat‐mediated antigen retrieval was performed in citrate buffer (pH 6.0, 95(C, 20 min). Sections were permeabilized with 0.5% Triton X‐100/PBS (15 min, RT), blocked in 5% donkey serum/1% BSA (60 min, RT), and incubated with rabbit anti‐ASXL2 antibody (1:200, ab106540, Abcam) overnight at 4(C. ASXL2 was detected using Alexa Fluor 555‐conjugated donkey anti‐rabbit IgG (1:500, A31572, Invitrogen, 60 min, RT) imaged at 561 nm excitation, followed by post‐fixation in 4% PFA (10 min). For AURKB mRNA visualization, sections underwent pre‐hybridization in buffer (10% formamide, 2× SSC, 37(C, 30 min), then hybridized with Stellaris CAL Fluor 488‐labeled AURKB probes (designed against NM_004217.4; 48 oligos, 40‐nt spacing) in hybridization buffer supplemented with 10% dextran sulfate overnight at 37(C. Stringent washes included formamide/SSC buffers (10% formamide/2× SSC, 37(C, 30 min; 2× SSC RT, 5 min ×2; 1× SSC RT, 2 min). Nuclei were counterstained with DAPI (1 µg/mL, 5 min, RT), mounted in ProLong Diamond Antifade Mountant, and imaged on an Olympus FV3000 confocal microscope using 405 nm (DAPI), 488 nm (AURKB puncta), and 561 nm (ASXL2) lasers.

### 5.31 Photobleaching

Fluorescence recovery after photobleaching (FRAP) experiments with cells infected with the pHBLV‐CMV‐TUBA1A‐GFP fusion protein were conducted using a laser scanning confocal microscope (LSCM) model LSM 900 from ZEISS. For epifluorescence excitation, a 63× objective lens with a numerical aperture (NA) of 1.4 and oil immersion was utilized, which was illuminated by a xenon lamp. The photobleaching process was carried out using a dedicated FRAP laser system, MicroPoint, manufactured by Photonic Instruments. Imaging was performed using a high‐quality CoolSNAP HQ camera, and the entire system was operated using SlideBook software.

Using a standard bleaching procedure, the cells were visualized within a single focal plane, capturing one frame per second for a duration of 3 s prior to the bleaching event. Following bleaching, the cells were continuously imaged for 180 s to monitor fluorescence recovery. This protocol allowed the observation of the dynamic behavior of α‐Tubulin within the cells, providing insights into the mobility and exchange rates of the protein.

### 5.32 RNA Isolation and Real‐Time Polymerase Chain Reaction

Total RNA was extracted from treated cells using 1 mL of TRIzol reagent (Sangon). cDNA was synthesized from 1 µg of total RNA through reverse transcription using a TaKaRa RNA PCR kit (TaKaRa Bio). The following sequences were used for the primers: CEP162 (forward, 5′‐*TCCTTATGGACAAAGCAGTGGTG*‐3′ and reverse, 5′‐*CAAATCGGACTCGGTGGTAGAG*‐3′), CEP290 (forward, 5′‐*ATGGAGCAGACAGTAGCAGAA*‐3′ and reverse, 5′‐*GGAGCCGTAGTTT GGTATTTTCA*‐3′), RPGRIP1L (forward, 5′‐*GCCGGTGAAAGATACA GGTCT*‐3′ and reverse, 5′‐*ACGCAAAAATCTGTCTTCCAGT*‐3′), TMEM67 (forward, 5′‐*CACCGCCTACCTTCTCTTAGT*‐3′ and reverse, 5′‐*GAGCGCCGAGATGTCAAAGT*‐3′), CEP164 (forward, 5′‐*AGAGTGACAACC AGAGTGTCC*‐3′ and reverse, 5′‐*GGAGACTCCTCGTACTCAAAGTT*‐3′), NPHP1 (forward, 5′‐*GATAGTTTGGTGACTGAAAGCCA*‐3′ and reverse, 5′‐*CCCACAGGTGCAGCTTCAT*‐3′), and PCM 1 (forward, 5′‐*CCACAGGAGGAGGTCCTTTTG*‐3′ and reverse, 5′‐CATATTGTTGTT). Quantitative real‐time PCR was performed using 1 µg of cDNA and SYBR Green (Bio‐Rad, California, USA) in 96‐well plates in a light cycler rapid thermal cycler system (MJ Research, Thermo Cycler). The expression of individual genes was calculated via a standard curve method and normalized to the expression of β‐actin.

### 5.33 Western Blotting

Total protein was extracted using a Western & IP cell lysis kit (Beyotime) containing 1 mM PMSF. Nuclear and cytoplasmic proteins were separated using a nuclear and cytoplasmic protein extraction kit (Beyotime) according to the manufacturer's instructions. Whole‐cell extracts were then separated using 10% sodium dodecyl sulfate–polyacrylamide gel electrophoresis and electrotransferred to a polyvinylidene difluoride (PVDF) membrane (Beyotime). After blocking, the membrane was incubated overnight at 4°C with rabbit polyclonal antibodies against ASXL2 (1:1000 dilution, ab106540, Abcam), EZH2 (1:1000 dilution, ab283270, Abcam), SUZ12 (1:1000 dilution, ab175187, Abcam), CEP162 (1:1000 dilution, PA5‐56383, Thermo), and β‐actin (1:1,000 dilution, AA128, Beyotime). The membranes were subsequently washed with TBST and incubated with horseradish peroxidase‐linked secondary antibodies (ZDR‐5306, ZDR‐5307, Zhong Shan, Beijing, China). After washing with TBST, immunoreactive bands were visualized using NBT/BCIP (Beyotime) as a substrate.

### 5.34 Coimmunoprecipitation

The coimmunoprecipitation (co‐IP) assay was performed as described previously [13]. In brief, the cells were lysed in co‐IP buffer (20 mM Tris, pH 7.5, 150 mM NaCl, 1% NP40, 10% glycerol and 1 mM EDTA) containing protease inhibitors (Beyotime, Shanghai, China) on ice for 60 min. Then, the cells were centrifuged, and the supernatants were collected, followed by incubation with primary antibodies with gentle rocking overnight at 4°C. On the following day, the mixture was pelleted, washed four times with prechilled buffer (4.0 ± 0.5°C; 20 mM Tris, pH 7.5, 150 mM NaCl, 0.1% NP40, 10% glycerol and 1 mM EDTA), and then analyzed by Western blotting.

### 5.35 CUT&Tag‐Seq

CUT&Tag was performed as described previously [29] using a CUT&Tag 4.0 High‐Sensitivity Kit (N259‐YH01; Novoprotein, Suzhou, China) in the mouse spermatogonial cell line G1 and the mouse spermatocyte line G2 (Procell, Wuhan, China). Briefly, formaldehyde‐crosslinked cells were thawed, and the nuclei were mixed with activated concanavalin A beads. The mixture was then magnetized to remove the liquid with a pipettor and resuspended in wash buffer (20 mM HEPES, pH 7.5; 150 mM NaCl; 0.5 mM spermidine; and Roche EDTA‐free protease inhibitor). After successive incubations with primary antibody (1–2 h) and secondary antibody (0.5–1 h) in wash buffer, the beads were washed and resuspended in 12.5 nM pA(G)‐Tn5 in 300‐wash buffer (wash buffer containing 300 mM NaCl) for 1 h. Incubations were performed at room temperature either in bulk or in volumes of 25–50 µL in low‐retention PCR tubes. For CUT&Tag, tagmentation was performed in a 1 h reaction in 300‐Wash buffer supplemented with 10 mM MgCl_2_ in a 50 µL volume. Termination buffer and protease K were added to the incubated sample, and the mixture was incubated at 55°C for 2 h to terminate the fragmentation reaction and prevent cross‐linking. DNA extraction and PCR amplification were performed to construct a DNA library and purify the PCR products. Supplementary Table S3 provides the quality control metrics for the CUT&Tag sequencing data, including Q30, mapping rate, duplication rate, rRNA contamination.

The samples were sequenced on the platform to obtain image files, which were then transformed by the sequencing platform's software to generate the original data in FASTQ format (raw data). Sequencing data often contain numerous connectors and low‐quality reads. Therefore, we first filtered the raw data using Cutadapt (v1.16, –discard‐trimmed ‐n 3 ‐e 0.1 ‐a AGATCGGAAGAGC ‐A) to remove these contaminants. This step ensured that we obtained high‐quality sequences (clean data) for further analysis.

Next, the filtered reads were aligned to the GRCm38 reference genome using Bowtie2 (v2.4.1, default parameters), and only uniquely mapped reads were retained. This alignment step ensured that we used only the reads that mapped to unique positions in the genome for downstream analysis.

Peaks were called using HOMER (v4.11.1) with default parameters (*p*‐value < 0.0001, fold enrichment ≥ 4). This ensured that we identify significant peaks in the data. The nearest gene to each peak within the group was extracted, and Venn analysis was performed using the R package VennDiagram (v1.6.20) to identify overlapping and unique peaks among different groups.

### 5.36 ChIP‒qPCR and Sequential ChIP‒qPCR

ChIP was performed as described previously. Briefly, precleared chromatin was immunoprecipitated with the following antibodies: anti‐ASXL2 (PA570292, Thermo), anti‐EZH2 (ab307646, Abcam), anti‐H3K27me3 (ab6002, Abcam) and nonspecific IgG (ab172730, Abcam). For sequential CUT&Tag, after the first antibody pulldown, the chromatin was further eluted with 10 mM DTT by gentle shaking at 37°C for 30 min, followed by a second antibody pulldown. The immunoprecipitated DNA and input DNA were quantified via qRT‒PCR analysis. Promoter binding enrichment was quantified as the percentage of the whole‐cell lysate relative to the input DNA. The fold enrichment was calculated by normalizing the specific antibody‐enriched chromatin to the nonspecific IgG‐enriched chromatin. The following sequences were used for the primers: CEP162 (forward, 5′‐ *TGGTGGATAGCCGAAGATGAT*‐3′ and reverse, 5′‐*GGGCTCAGAAACAACCAATTCA*‐3′) and CEP290 (forward, 5′‐*GAGGGGCTGGTAG AAGTGG*‐3′ and reverse, 5′‐*CCGTTCATTTTCGGC AACAATTT*‐3′).

### 5.37 Coimmunoprecipitation of Chromatin‐Immunoprecipitated Complexes

To map ASXL2‐EZH2 co‐occupied genomic loci, primary round spermatids (10^7^/sample) were crosslinked with formaldehyde (1%, 10 min), quenched with glycine, lysed sequentially in buffers LB1 (50 mM HEPES‐KOH (pH 7.5), 140 mM NaCl, 0.5% NP‐40/0.25% Triton X‐100) and LB2 (10 mM Tris‐HCl (pH 8.0), 200 mM NaCl), and chromatin‐sheared to 200–500 bp fragments (Bioruptor Pico); for primary ChIP, 5 µg of anti‐ASXL2 (PA570292, Thermo) was used with Protein A/G Magnetic Beads followed by stringent washes (low/high‐salt, LiCl, TE buffers), after which ASXL2‐bound complexes were coimmunoprecipitated in NP‐40 buffer (50 mM Tris‐HCl (pH 7.4), 150 mM NaCl, 1% NP‐40) with 5 µg of anti‐EZH2 (ab307646, Abcam) alongside controls (IgG); DNA was recovered through crosslink reversal, purified, and processed into sequencing libraries for 150‐bp paired‐end sequencing on an Illumina NovaSeq 6000.

### 5.38 Molecular Dynamics Simulations

The amino acid sequences of ASXL2 (UniProt ID: Q76L83) and EZH2 (UniProt ID: Q15910) were obtained from the UniProt database. The structural models of the ASXL2/EZH2 protein complex were predicted using the AlphaFold Server (https://alphafoldserver.com/), generating multiple candidate conformations. To evaluate the binding affinities of these predicted complexes, the binding free energies were calculated using the Uni‐GBSA method (https://github.com/dptech‐corp/Uni‐GBSA). On the basis of the calculated free energy values, the most thermodynamically favorable conformation was selected for further analysis. A full‐atom molecular dynamics (MD) simulation was subsequently carried out using GROMACS 2023.1 to assess the stability and dynamic behavior of the selected complex over time. Residue‐wise decomposition of the binding free energy was performed to identify key interacting residues that contribute significantly to complex stability across different conformations. These critical residues were then mapped onto the 3D structure of the complex to determine functionally relevant interaction hotspots, providing insights into the molecular determinants of ASXL2/EZH2 complex formation and stabilization.

### 5.39 Electrophoretic Mobility Shift Assays

The promoter regions of *CEP162* were amplified via PCR and subsequently biotinylated using the Biotin 3′ End Labeling Kit (Beyotime Biotechnology, GS008) following the manufacturer's protocol for annealing. The EMSA probes spanned chr9:87 252 022–87 252 051 (mm10; sequence: 5′‐TCTCTAGATGGTCCATCCTTTTGTCTCAGC‐3′), covering a critical regulatory region upstream of the transcriptional start site. Either ASXL2/EZH2 (10 µg) or histone H3 trimethyl lysine 27 (10 µg) was incubated with 10 nM biotin‐labeled DNA oligonucleotides in binding buffer (Beyotime Biotechnology, GS009) for 20 min at room temperature. The reaction mixtures were then subjected to electrophoresis on 1 mm‐thick 15% nondenaturing polyacrylamide gels and transferred onto Hybond‐N+ membranes (Beyotime Biotechnology, FFN13). The DNA oligomers were fixed to the membrane using UV crosslinking, and the labeled probes were detected using a LightShift Chemiluminescent EMSA Kit (Beyotime Biotechnology, GS009).

### 5.40 RNA‐Seq

After being subjected to hypoxia (11.7% oxygen concentration) or normoxia (21% oxygen concentration) as a control for 10 w, spermatogenic cells were collected, and total RNA was extracted using TRIzol reagent (Invitrogen, Carlsbad, CA, USA). RNA‐seq analysis was performed by Personalbio Genomic Technology Co., Ltd. (Shanghai, China). Briefly, total mRNA was extracted with a Dynabeads mRNA DIRECT Kit (Ambion). Thereafter, a cDNA library was constructed using the NEBNext Ultra Directional RNA Library Prep Kit for Illumina (New England Biolabs, Inc.). After fragmentation and conjugation with sequencing adaptors, the cDNA libraries were sequenced on an Illumina HiSeq 2500 platform using the PE100 strategy. The raw RNA‐seq data were filtered and mapped to a reference genome (Mus_musculus. GRCm38.90. chr) by using FASTX. The fragments per kilobase of exon model per million reads mapped (FPKM) value was calculated from the filtered and mapped clean raw reads. Furthermore, we used the tximport package with default parameters to remove abnormally low‐/high‐abundance transcripts from the transcriptome profiles. Eventually, the DEGs were obtained from DESeq2 analysis and used for further bioinformatics studies.

### 5.41 Gene Expression Dataset Analysis

To determine the mRNA expression levels of ASXL2, EZH2, SUZ12, EED, YY1, BRD1, WDR5, RBBP4, RNF2, and USP7 in NOA patients, we conducted a transcriptome analysis using public RNA‐seq datasets. We collected normalized gene expression data for NOA from GSE6872, GSE6967, and GSE6968 and for teratozoospermic patients from GSE190752, GSE216907, and GSE145467. The raw sequencing fastq files were sourced from the NCBI Sequence Read Archive and mapped to the human reference genome hg38 using STAR [61]. Gene expression was quantified using the TPM/FPKM/Log (Cy5/Cy3 ratio) Calculator, which uses transcripts per million (TPM), fragments per kilobase million (FPKM), and the logarithmic ratio of fluorescence intensities (Log(Cy5/Cy3 ratio)) to normalize gene expression across different samples. We categorized samples into high‐ or low‐expression groups on the basis of the mean TPM/FPKM/Log (Cy5/Cy3 ratio) values, labeling those above the mean as “high‐level” and those below as “low‐level.” This method ensured a standardized comparison of gene expression profiles across various patient groups.

### 5.42 Immunohistochemistry

Human semen samples (from 6 teratozoospermic patients, 6 healthy controls, 1 nonobstructive azoospermia patient, and 1 additional normozoospermic control) were fixed in 4% paraformaldehyde (PFA) for 24 h, dehydrated through a graded ethanol series, and embedded in paraffin. Tissue sections (4 µm thickness) were mounted on poly‐L‐lysine‐coated slides. Antigen retrieval was performed by boiling the slides in 10 mM citrate buffer (pH 6.0) for 20 min in a microwave. Endogenous peroxidase activity was quenched with 3% H_2_O_2_ in methanol for 10 min. The sections were blocked with 5% bovine serum albumin (BSA) in PBS (pH 7.4) for 1 h at room temperature (RT) to reduce nonspecific binding. The following primary antibodies were applied overnight at 4°C: anti‐ASXL2 (1:200 dilution, Abcam, cat# ab12345, clone EPR23456) and anti‐EZH2 (1:300 dilution, Cell Signaling Technology, cat# 6789S, clone D2C9). After being washed with PBS, the sections were incubated with HRP‐conjugated secondary antibodies (goat anti‐rabbit IgG, 1:500, Vector Laboratories, cat# PI‐1000) for 1 h at RT. Signal detection was performed using a DAB substrate kit (Vector Laboratories, cat# SK‐4100) for 5 min, followed by counterstaining with Mayer's hematoxylin for 30 s. The slides were dehydrated, cleared in xylene, and mounted with Permount mounting medium (Fisher Scientific). Bright‐field images were captured using a Nikon Eclipse Ni‐E microscope (20× objective) equipped with a DS‐Ri2 camera. The staining intensity was quantified using ImageJ (v1.53) with thresholding set to exclude background signals (threshold: 150–255 grayscale). IHC staining was evaluated using the H‐score system. Positive cells were defined as those with DAB intensity ≥2‐fold above background. All procedures were approved by the Medical Ethics Committee of the Second Affiliated Hospital of Army Medical University, PLA (Approval Number: 2025‐135‐01), and informed consent was obtained from all participants.

### 5.43 Sequencing and Analysis

A Qubit Fluorometer or microplate reader (PicoGreen fluorescence quantification) was used to quantify the samples, and an Agilent 2100 was used for quality control to verify the size and distribution of the DNA fragments. Through the interaction of 3'‐5' exonuclease and polymerase, DNA fragments with protruding ends were repaired. A single “A” base was introduced at the 3' end of the repaired flat DNA fragment, and the 3' end of the connector contained a single “T” base to ensure that the DNA fragment and the connector could be connected through “A” and “T” complementary pairing and prevent the DNA insert fragments from annealing with each other during this process. Under the action of ligase, the labeled splices were incubated to bind to DNA fragments. VAHTS DNA clean beads were used to purify the free splices and segments without splices. PCR was used to selectively enrich DNA fragments with connectors at both ends and amplify the DNA library in fewer cycles to avoid errors in PCR amplification. An Agilent 2100 instrument was used to control the quality of the PCR‐enriched fragments and verify the size and distribution of the DNA library fragments. Multiplexed DNA libraries were homogenized to 10 nM and then mixed in equal volumes. The mixed library (10 nM) was diluted to 4∼55 pM and sequenced. Next‐generation sequencing (NGS) was used to perform paired‐end sequencing on these libraries (paired‐end, PE) via the Illumina sequencing platform. The Venn diagrams and average profiles of the RefSeq gene bodies were generated using ngsplot.

### 5.44 Statistical Analysis

For the RNA‐seq data, the raw counts were normalized using the R package DESeq2 (v1.36.0) with variance‐stabilizing transformation. For the Cut&Tag sequencing peak data, normalization was performed to account for sequencing depth and technical variation, followed by log‐transformation to stabilize variance and improve normality. Flow cytometry and immunohistochemistry data were background‐corrected and normalized using z‐score transformation where applicable. All quantitative data are presented as the mean ± standard deviation (SD), with details provided in the figure legends. The sample size (n) for each analysis is explicitly stated in the legends to ensure transparency. Replicates (biological or technical) are clearly indicated for all the assays. Statistical significance was assessed using the following methods: 2‐tailed, unpaired Student's t‐test for pairwise comparisons; 1‐way ANOVA with Tukey's post hoc test for multiple groups, or 2‐way repeated‐measures ANOVA with Tukey's post hoc test for repeated measurements. Adjusted P‐values (family‐wise error rates) are reported using the standard notation: ns (not significant), *p < 0.05, **p < 0.01, ***p < 0.001, ****p < 0.0001. All analyses were conducted using GraphPad Prism (version 9.0) to ensure rigor and reproducibility.

## Author Contributions

Conceptualization: B. Ni, Z. Zao, and Y. Zhang. Methodology: J. Yin, M. Zhang, W. Liu, W. Shen, and G. Zhang. Investigation: J. Yin, M. Zhang, W. Liu, W. Shen, and D. Li. Visualization: M. Zhang, H. Miao, and F. Deng. Data Analysis: J. Yin, Y. Zhang, D. Li, and Y. Tian. Supervision: B. Ni and Z. Zao. Writing – Original Draft: B. Ni and J. Yin. Writing – Review & Editing: B. Ni, Z. Zao, and Y. Zhang.

## Conflicts of Interest

The authors declare no conflict of interest.

## Supporting information




**Supporting File 1**: advs74564‐sup‐0001‐SuppMat.docx

**Supporting File 2**: advs74564‐sup‐0002‐SupplementaryTable1.xlsx

**Supporting File 3**: advs74564‐sup‐0003‐SupplementaryTable2.xlsx

**Supporting File 4**: advs74564‐sup‐0004‐SupplementaryTable3.xlsx

**Supporting File 5**: advs74564‐sup‐0005‐Data.zip

## Data Availability

The data that support the findings of this study are available in the supplementary material of this article.
