# Supplementary material for: Chronic Hypoxia Disrupts Spermatogenesis Through ASXL2–EZH2–Mediated Microtubule Destabilization
Source: Adv Sci (Weinh). 2026 Mar 4;13(26):e01266. doi: 10.1002/advs.202501266 (PMC13159132; doi:10.1002/advs.202501266)
Supplement: Supplementary file 1 — Supporting File 1: advs74564‐sup‐0001‐SuppMat.docx [file ADVS-13-e01266-s003.docx]

Supplementary Figures for

**Chronic Hypoxia Disrupts Spermatogenesis through ASXL2–EZH2–Mediated Microtubule Destabilization**

Jun Yin^1,2,3†^, Mengjie Zhang^1,2,3†^, Wenying Liu^1,2,3,4†^, Wenlong Shen^5†^, Debao Li^6^, Hongming Miao^1,2,3^, Fang Deng^1,2,3^, Gang Zhang^2,3^, Yi Tian^7^, Yi Zhang^6*^, Zhihu Zhao^5*^, Bing Ni^1,2,3*^

^1^ Department of Pathophysiology, Army Medical University, Chongqing 400038, China

^2^ Key Laboratory of Extreme Environmental Medicine, Ministry of Education of China, Chongqing 400038, China

^3^ Key Laboratory of High Altitude Medicine, PLA, Chongqing 400038, China

^4^ Department of Dermatology, Southwest Hospital, Army Medical University, Chongqing 400038, China

^5^ Laboratory of Advanced Biotechnology, Beijing Institute of Biotechnology, Beijing 100071, China

^6^ Chongqing International Institute for Immunology, Chongqing 401338, China

^7^ Department of Immunology, Army Medical University, Chongqing 400038, China

^†^These authors contributed equally

^*^To whom correspondence should be addressed:

Yi Zhang, Tel: +86-23-68752188; email: zhangyi@iiicq.vip

Zhihu Zhao, Tel: +86-10-65334657; email: zhaozh@bmi.ac.cn

Bing Ni, Tel: +86-23-68771740; email: [nibing@tmmu.edu.cn](mailto:nibing@tmmu.edu.cn); ORCID ID：0000-0002-4297-5346

**This document includes:** Supplementary Figures 1 to 8 (Figures S1-S8)

**Supplementary Figure 1**


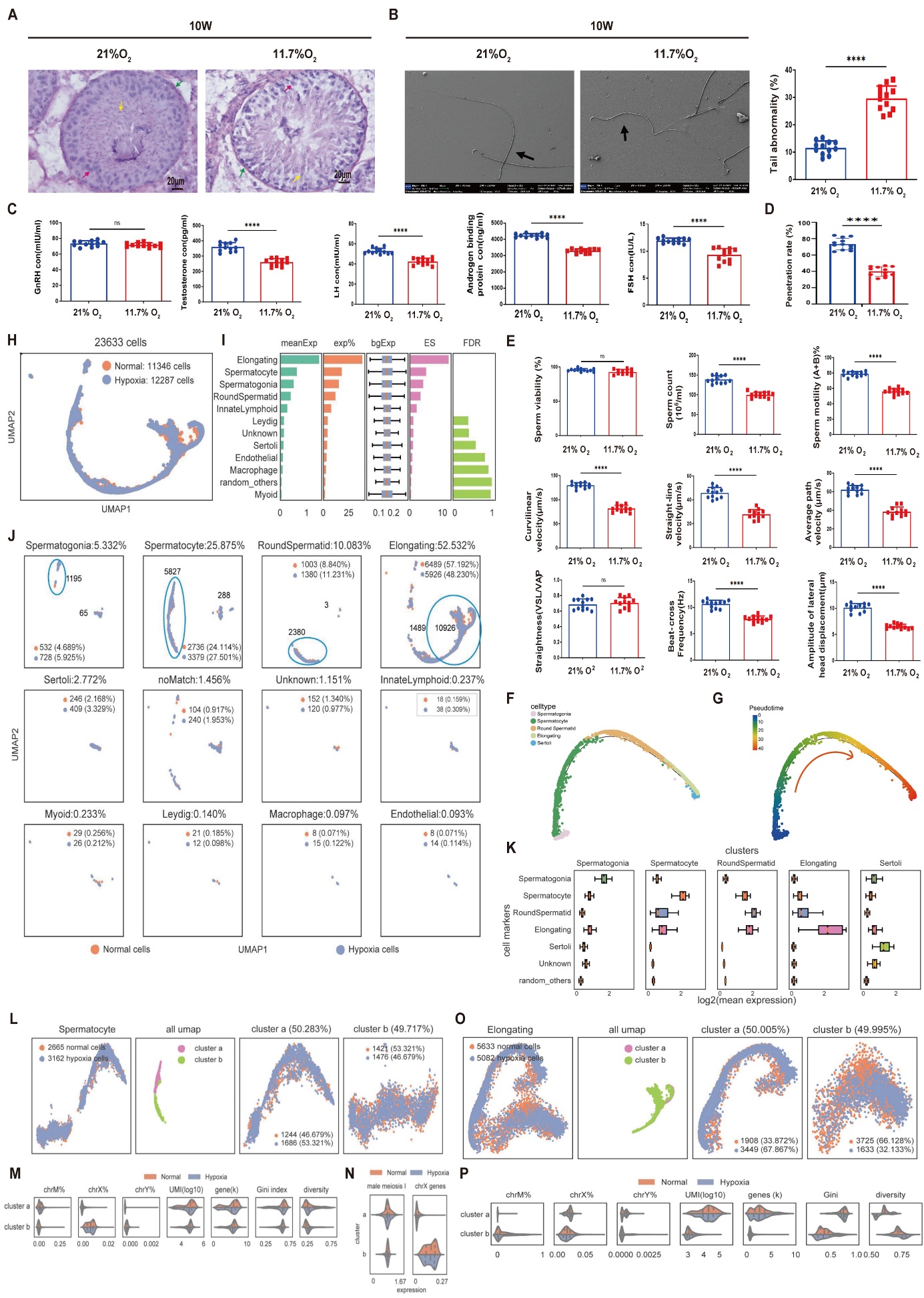


**Supplementary Figure 1.** Chronic hypoxia inhibits the transition of round–elongating spermatids.

**(A)** Glycogenic acid–Schiff/PAS staining of seminiferous tubule sections. The figure shows the morphological changes in the seminiferous tubules in two different fields of view in the normoxic and hypoxic groups. The green arrow represents spermatogonia, the red arrow represents spermatocytes, and the yellow arrow represents round spermatids. **(B)** Scanning electron micrographs of the sperm of the flagella. Quantitative analysis of tail abnormalities between normoxic and hypoxic conditions (n = 12). **(C)** Serum levels of sex hormones in rats exposed to 11.7% O2 for 10 weeks (n = 12). GnRH, gonadotropin-releasing hormone (n = 12); LH, luteinizing hormone; FSH, follicle-stimulating hormone; T, testosterone; ABP, androgen-binding protein. **(D)** Acetic acid magenta staining was used to observe the entry of spermatogonia into oocytes, and the penetration rate of sperm into oocytes was calculated (%) (n = 12). **(E)** The viability of sperm subjected to 11.7% oxygen for 10 weeks (n = 12). The sperm count (n = 12) and sperm motility parameters, including progressive motility (n = 12), curvilinear velocity (n = 12), straight-line velocity (n = 12), average path velocity (n = 12), straightness (n = 12), beat-cross frequency (n = 12) and amplitude of lateral head displacement (n = 12), were examined with a sperm class analyzer system (CSA, Microptic S.L., Barcelona, Spain). Putative cell type assignment for each cell. **(F)** All cells identified in Figure 1b H were subjected to pseudotemporal ordering and trajectory inference using Monocle2. Distribution of distinct cell types across different positions along the inferred developmental trajectory. **(G)** The red arrow indicates the direction of cellular differentiation. The color gradient from blue to red represents advancing pseudotime, corresponding to progression from early to late stages of development. **(H)** Scatter plot for each cell of the first two reduced components from the expression matrix processed by UMAP (n = 3). **(I)** Example cell type assignment of a cell classified as a putative elongating spermatid cell. The mean expression values (first column panel) and number of expressed genes (second column panel) of the marker genes obtained from 15 datasets were calculated for each cell type. One thousand permutations of the cell’s gene expression values were established to obtain the expected background expression for each cell type marker gene (third column panel). The enrichment score (ES, fourth column panel) and FDR (fifth column panel) were calculated on the basis of marker gene expression values and background distribution. Expressed marker gene ratios >=20%, FDR <=0.001 and ES >=5 were used to obtain putative cell types, and the cell type with the highest ES was assigned to the cell. **(J)** Scatter plot of all putative cell types in the UMAP plot from (D). The cells in separate minor groups were discarded, and the blue circles indicate the cells used for subsequent analysis. Overall, only cells in putative cell types comprising more than 2% of all cells, namely, spermatogonia, spermatocytes, round spermatids, elongating spermatids and Sertoli cells, were considered for subsequent analysis. **(K)** Marker gene expression distribution in putative cell types. Putative subtypes of spermatocytes. **(L)** Scatter plots of identified putative spermatocyte subtypes. The first column is a magnified view of the UMAP plot for spermatocytes. The first two components of UMAP were obtained from the expression matrix of putative spermatocyte cells. The second column shows the putative subtype locations on the scale of whole UMAP plots. The scale is the same as that in Figure 1H, and the first two components of UMAP are the same as those in Figure 1H. The third column is a magnified view of the UMAP plot for subtype Cluster A, and the two components are the same as those of the first column plot. The last column is a magnified view of the UMAP plot for subtype Cluster B, and the two components are the same as those of the first column plot. **(M)** Distribution profiles of cell attributes for the two putative subtypes. **(N)** Expression distribution of male meiosis I genes and chromosomal X genes. Putative subtypes of elongating spermatids. **(O)** Scatter plots of identified putative elongating spermatid subtypes. The first column is a magnified view of the principal component analysis (PCA) plot for elongating spermatids. The first two PCA components were obtained from the expression matrix of putative elongating spermatid cells. The second column shows the putative subtype locations on the scale of the whole UMAP plot. The scale is the same as that in Figure 1H, and the first two components of UMAP are the same as those in Figure 1H. The third column is a magnified view of the PCA plot for subtype Cluster A, and the two components are the same as those of the first column plot. The last column is a magnified view of the PCA plot for subtype Cluster B, and the two components are the same as those of the first column plot. **(P)** Distribution profiles of cell attributes for the two putative subtypes. The data are presented as the means ± SDs; ns nonsignificant, *****P* < 0.0001, as determined by 2-tailed, unpaired Student’s t test (B, C, D, and E).

**Supplementary Figure 2**


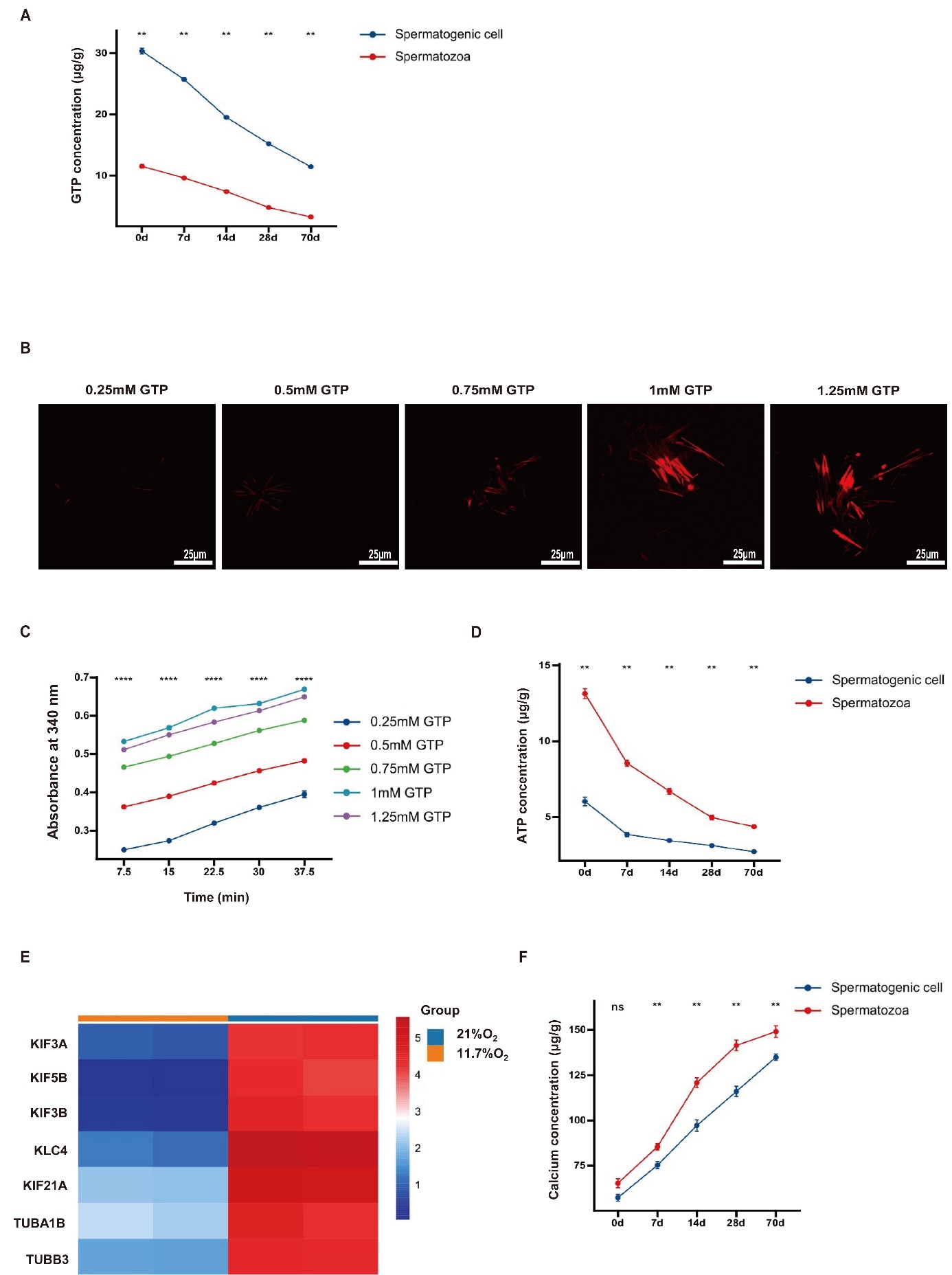


**Supplementary Figure 2.** Chronic hypoxia induces instability in the microtubules of cilia, accompanied by a reduction in ATP and GTP levels, as well as an increase in calcium ions, in both spermatogonial cells and sperm.

**(A)** GTP concentrations in both spermatogenic cells and spermatozoa were analyzed via liquid chromatography‒mass spectrometry (n = 6). **(B)** Microtubules were assembled with rhodamine-labeled and unlabeled tubulin (1:9) in the presence of Taxol (20 μM). The assembled microtubules were then supplemented with the indicated concentrations of GTP (0.25 mM, 0.5 mM, 0.75 mM, 1 mM and 1.25 mM) and processed for confocal microscopy. Scale bar, 20 μm. **(C)** Microtubules were assembled with tubulin (17 μM) in the presence of Taxol (20 μM) at the indicated GTP concentrations (0.25 mM, 0.5 mM, 0.75 mM, 1 mM and 1.25 mM) and then measured by light scattering at a wavelength of 340 nm with a microplate reader. The data were analyzed with GraphPad software (n = 6). **(D)** The concentrations of ATP in both spermatogenic cells and spermatozoa were quantified via liquid chromatography‒mass spectrometry (n = 6). **(E)** Following 10 weeks of exposure to 11.7% oxygen in rats, sperm cilia were isolated via density gradient centrifugation. Subsequent quantitative proteomics and bioinformatics analyses revealed the distributions of KIF3A, KIF3B, KIF5B, KIF21A, KLC4, TUBA1B, and TUBB3 within the cilia through heatmap visualization (n = 3). **(F)** The calcium ion concentration within both spermatogenic cells and spermatozoa was determined via liquid chromatography‒mass spectrometry (n = 6). The data are presented as the means ± SDs; ns nonsignificant, ***P* < 0.01, *****P* < 0.0001, as determined by 2-tailed, unpaired Student’s t test (A, C, D, and F).

**Supplementary Figure 3a
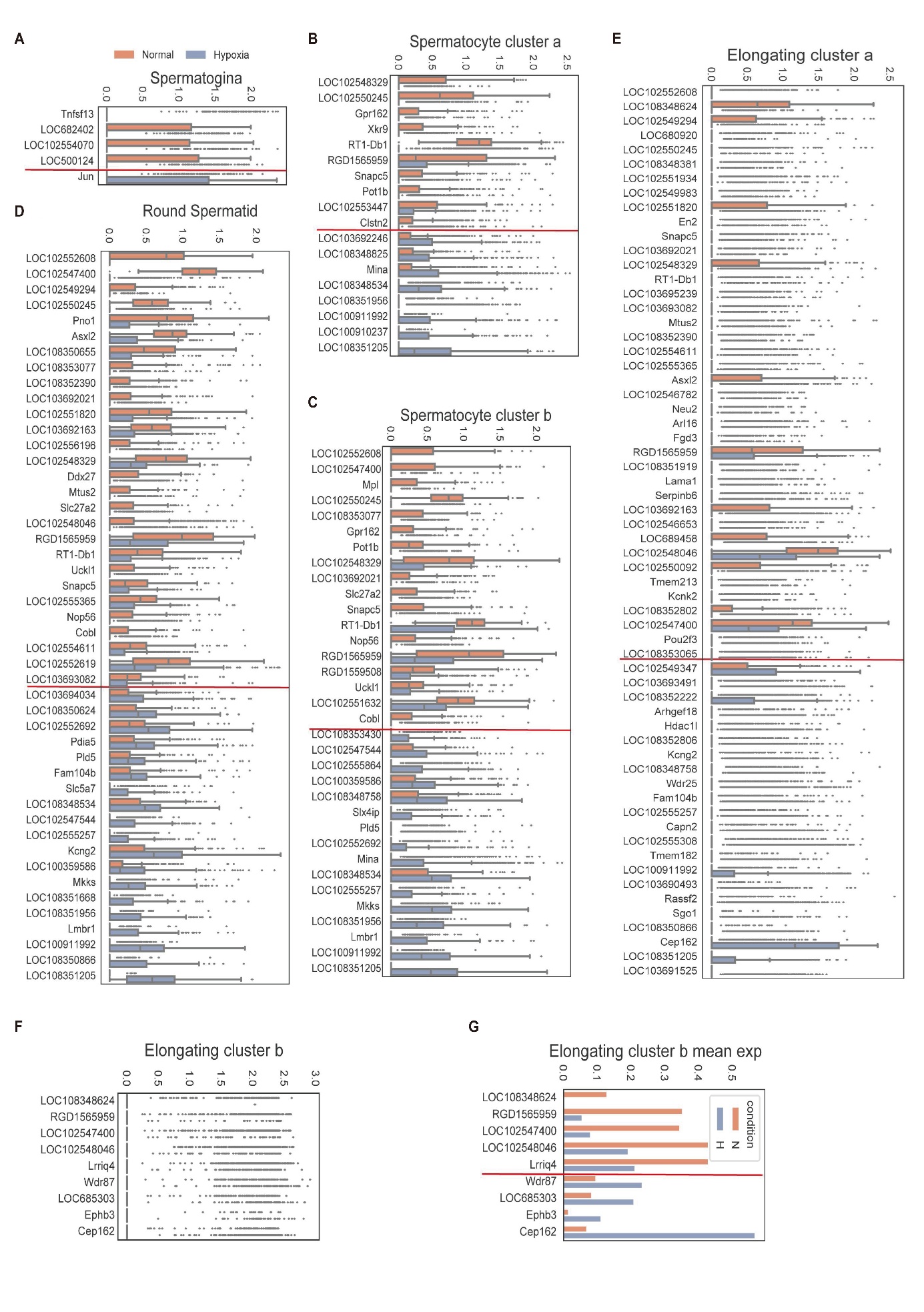
**

**Supplementary Figure 3a (A-G).** All DEGs between the control and hypoxia treatments. Boxplots of DEGs between control and hypoxia-treated spermatogonia **(A)**, Cluster A spermatocytes **(B)**, Cluster B spermatocytes **(C)**, round spermatids **(D)**, Cluster A elongating spermatids **(E)** and Cluster B elongating spermatids **(F)**. **(G)** Bar plots of the mean expression of identified DEGs in elongating spermatid Cluster B; boxplots are not shown in (F). Genes above the red lines are expressed at relatively high levels in normal rat testes, whereas genes below the red lines are expressed at relatively high levels in hypoxia-treated rat testes.

**Supplementary Figure 3b**
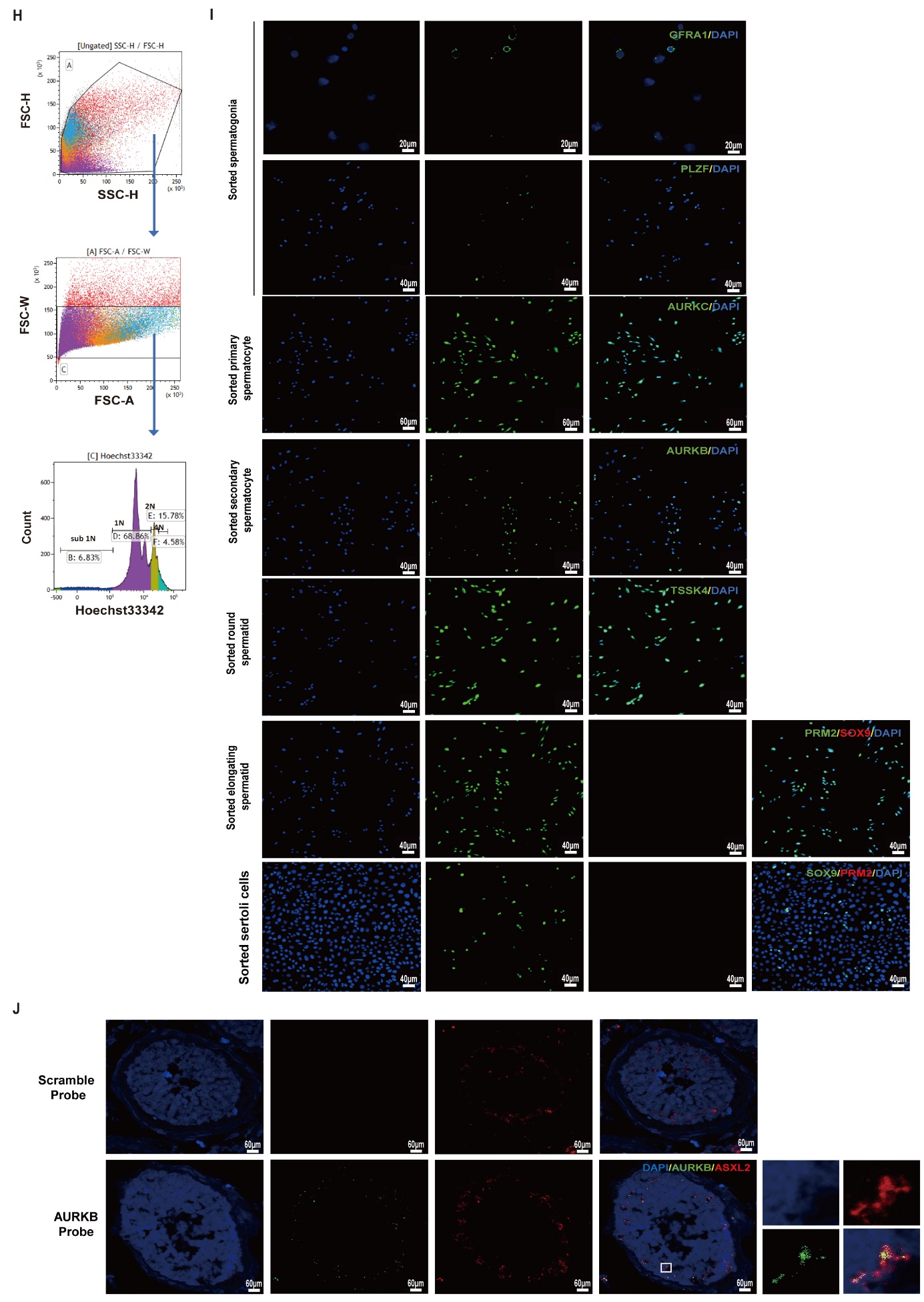


**Supplementary Figure 3b (H-J).** Validation of spermatogenic cell identity and ASXL2 expression analysis. **(H)** Spermatogenic cells were stained with Dye780 and Hoechst 33342, and round spermatids and elongating spermatids were sorted using flow cytometry. Live cells were identified using Dye780, while round spermatids and elongating spermatids were sorted on the basis of the fluorescence intensity of Hoechst 33342. **(I)** Immunofluorescence staining demonstrates cell-type-specific expression of TSSK4, a marker for round spermatids, and PRM2, an elongating spermatid-specific nuclear protein. Both proteins are visualized in green, with nuclei counterstained using DAPI (blue); scale bar = 40 μm. Spermatogonia were identified based on co-expression of GFRA1 and PLZF. Confocal microscopy images show that both markers localize to the same cell population (green), with nuclear staining by DAPI (blue); scale bar for GFRA1 is 20 μm and scale bar for PLZF is 40 μm. AURKC, specific to primary spermatocytes, and AURKB, specific to secondary spermatocytes, were further validated through immunofluorescence. Confocal micrographs of AURKC-positive primary spermatocytes and AURKB-positive secondary spermatocytes (green). Nuclei are labeled with DAPI (blue). Scale bars: 60 μm (AURKC), 40 μm (AURKB). Sertoli cells were specifically identified by SOX9 immunostaining; scale bar = 40 μm. **(J)** Sequential immunofluorescence (IF)/smFISH analysis reveals ASXL2 expression in secondary spermatocytes within seminiferous tubules. AURKB serves as a specific marker for secondary spermatocytes. The boxed regions highlight AURKB-positive cells exhibiting detectable ASXL2 signals. Nuclei were counterstained with DAPI; scale bar = 40 μm.

**Supplementary Figure 4a**

**
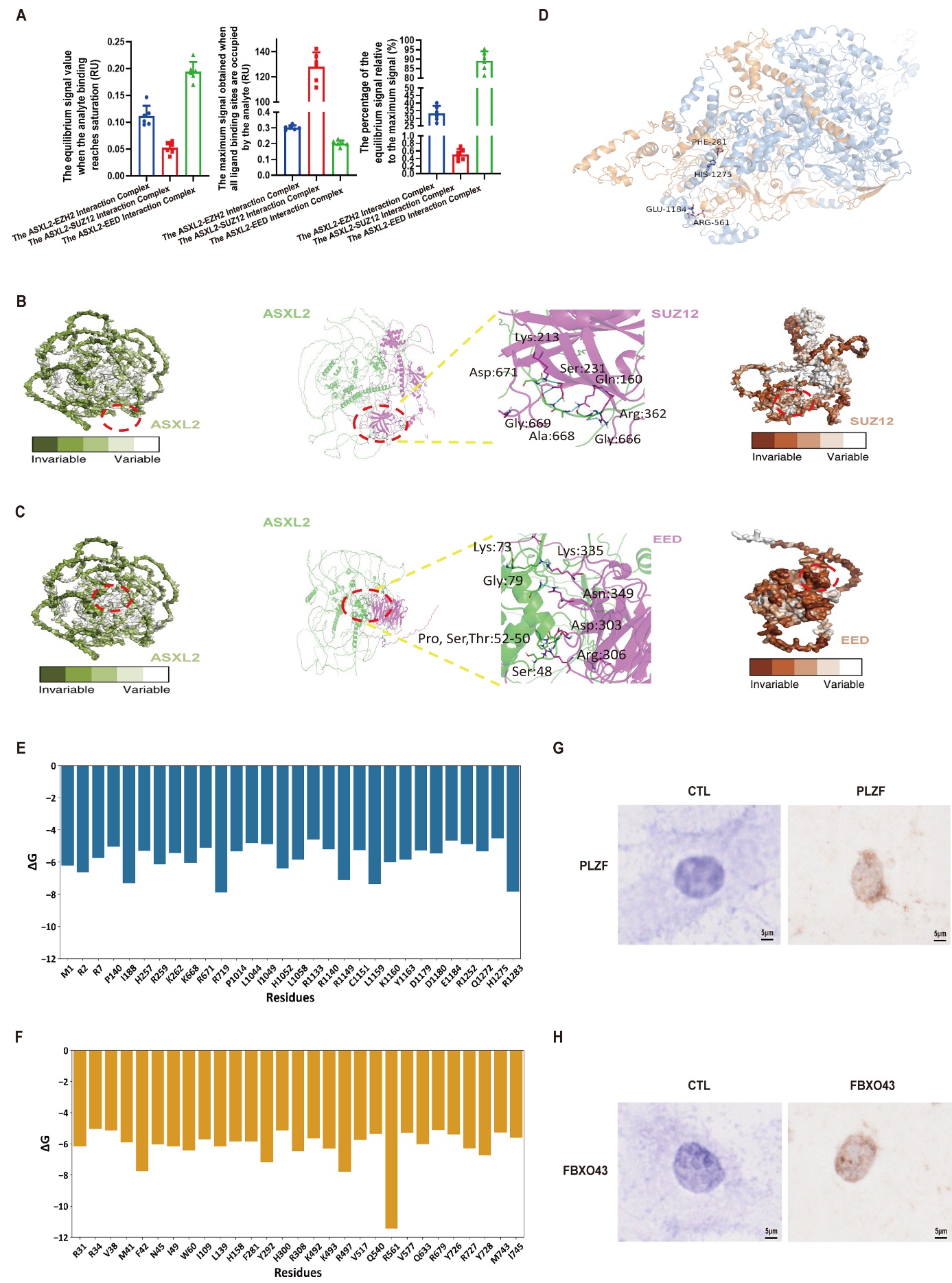
**

**Supplementary Figure 4a (A-H).** Bioinformatic analysis of ASXL2/EZH2 targets under hypoxia.

**(A)** The figure shows the signal values when ASXL2 binds to EZH2, SUZ12, and EED at equilibrium (Req), the maximum signal value when all the ligands are occupied by the analyte (Rmax), and the percentage of Req relative to Rmax (Req/Rmax (%). Note: Req/Rmax (%) reflects the saturation level, indicating the percentage of maximum binding capacity reached under the given conditions (n = 6)**.** The data are presented as the means ± SDs. This panel elucidates the interaction processes between **(B)** ASXL2 and SUZ12, as well as **(C)** ASXL2 and EED, along with the subsequent formation of hydrogen bonds. **(D)** Structural snapshot of the ASXL2-EZH2 complex at 100 ns MD simulation. ASXL2 is shown in cyan, EZH2 in magenta. Key interacting residues (ASXL2: HIS-1275, GLU-1184; EZH2: PHE-281, ARG-561) are highlighted as sticks. Hydrogen bonds are depicted as yellow dashed lines. **(E)** Binding free energy contribution (MMGBSA) of key ASXL2 residues interacting with EZH2. Residues contributing ≤ −4.0 kcal/mol are labeled. **(F)** Corresponding binding free energy contribution (MMGBSA) of key EZH2 residues interacting with ASXL2. Residues with contributions ≤ −4.0 kcal/mol are indicated. Residues contributing ≤ −4.0 kcal/mol are labeled. **(G)** Immunocytochemical staining of PLZF in G1 cells. **(H)** Immunocytochemical staining of FBXO43 in G2 cells.

**Supplementary Figure 4b**


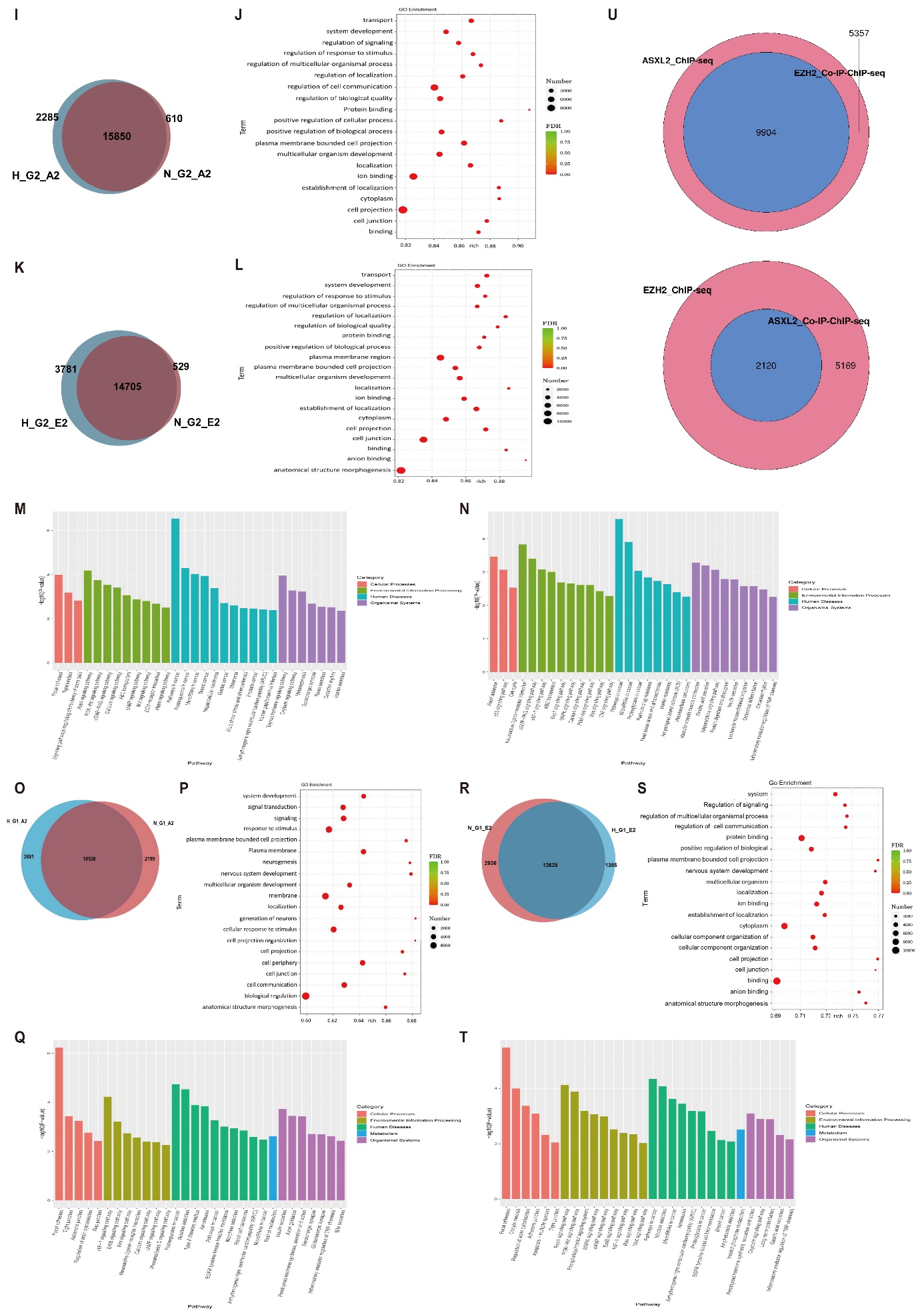


**Supplementary Figure 4b (I-U).** Identification of ASXL2/EZH2 targets via high-throughput CUT&Tag-seq.

**(I)** Venn diagram analysis of 2,285 DEGs, which are unique target genes of ASXL2, in G2 cells subjected to 1% hypoxia for 48 h. **(J)** GO analysis of the 2,285 DEGs with a |log2FC| ≥ 2. **(K)** Venn diagram analysis of 3,781 differential peak nearest genes, which are the unique target genes of EZH2 in G2 cells after exposure to 1% hypoxia for 48 h. **(L)** GO analysis of the 3,781 DEGs with a |log2FC| ≥ 2. N, normal condition; H, hypoxia; G1, G1 cells; G2, G2 cells; E2, EZH2; A2, ASXL2. **(M)** KEGG enrichment analysis of the 2285 DEGs, which are unique target genes of ASXL2, in G2 cells subjected to 1% hypoxia for 48 h. **(N)** KEGG enrichment analysis of 3781 DEGs, which are unique target genes of EZH2, in G2 cells subjected to 1% hypoxia for 48 h. Biological bioinformatics analyses of ASXL2 target genes induced in G1 cells by hypoxia. G1 cells were exposed to 1% hypoxia for 48 h, and the cells were harvested for CUT&Tag-seq. **(O)** Venn diagram analysis of the 2881 DEGs, which are unique target genes of ASXL2, in G1 cells subjected to 1% hypoxia for 48 h. **(P)** GO analysis of the 2881 DEGs with a |log2FC| ≥ 2. **(Q)** KEGG enrichment analysis of the 2881 DEGs. Biological bioinformatics analyses of EZH2 target genes induced in G1 cells by hypoxia. G1 cells were exposed to 1% hypoxia for 48 h, and the cells were harvested for CUT&Tag-seq. **(R)** Venn diagram analysis of 1385 DEGs, which are unique target genes of EZH2 in G1 cells after exposure to 1% hypoxia for 48 h. **(S)** GO analysis of 1385 DEGs with a |log2FC| ≥ 2. **(T)** KEGG enrichment analysis of 1385 DEGs. **(U)** Venn diagram quantifying genomic co-occupancy of ASXL2 and EZH2 identified by Co-IP of ChIP-seq.

**Supplementary Figure 5**


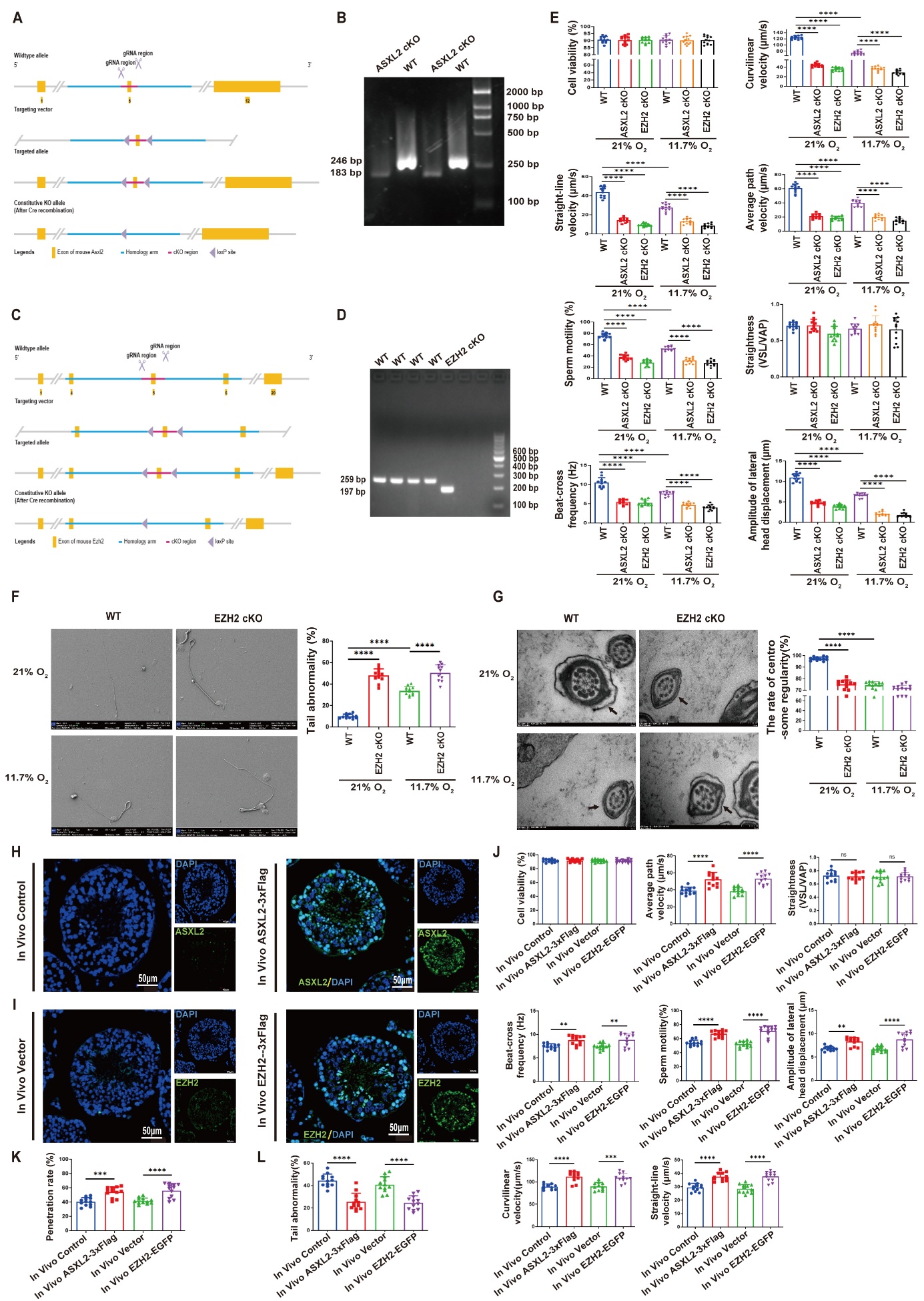


**Supplementary Figure 5.** Exogenously stimulating the ASXL2‒PRC2 axis notably corrects sperm tail deformities and enhances sperm motion.

**(A)** Schematic diagram depicting the construct used to generate spermatogenic cell-specific conditional Asxl2-knockout (KO) mice. Exon 5 of the mouse Asxl2 gene is flanked by loxP sites in these Asxl2 conditional knockout mice. Cre recombinase-mediated excision of the floxed region results in a knockout allele. **(B)** Genotype identification of sperm-specific knockout ASXL2 mice. Note: The size of the ASXL2 gene fragment in wild-type mice is 246 bp, whereas the size of the ASXL2 gene fragment in ASXL2 conditional knockout mice is 183 bp. **(C)** Schematic diagram depicting the construct used to generate spermatogenic cell-specific conditional EZH2-knockout (KO) mice. In these EZH2 conditional knockout mice, exon 5 of the mouse EZH2 gene is flanked by loxP sites. Cre recombinase-mediated excision of the floxed region results in a knockout allele. **(D)** Genotype identification of EZH2 knockout mice. Note: The size of the EZH2 gene fragment in the wild-type mice was 259 bp, whereas the size of the EZH2 gene fragment in the EZH2 conditional knockout mice was 197 bp. **(E)** WT, ASXL2^-/-^ and EZH2^-/-^ mice were housed under normal (21% O2 content) or hypoxic (11.7% O2) conditions for 10 weeks, the testes were harvested, and the spermatogenic cells in the testes were separated via digestion. The sperm in the epididymis were collected for serial examinations. Viability of sperm subjected to 11.7% oxygen for 10 weeks (n = 12). The sperm motility parameters, including progressive motility (n = 12), curvilinear velocity (n = 12), straightness (n = 12), straight-line velocity (n = 12), beat-cross frequency (n = 12), average path velocity (n = 12) and amplitude of lateral head displacement (n = 12), were examined with a sperm class analyzer system (CSA, Microptic S.L., Barcelona, Spain). Generation of spermatogenic cell-specific conditional Asxl2/EZH2 knockout (KO) mice. Exogenous overexpression of ASXL2/EZH2 in mice. **(F)** Papanicolaou staining (×400) was used to observe sperm flagella morphology and quantify tail abnormalities between WT and EZH2^-/-^ mice (n = 12). **(G)** TEM images of sperm flagella were analyzed to quantify tail abnormalities (n = 12). Cross-sectional TEM of spermatozoa from EZH2^-/-^ mice revealed missing DMTs (indicated by black arrows). Additionally, centrosome regularities were quantitatively assessed in EZH2^-/-^ mice. **(H)** Immunostaining of ASXL2 10 weeks after microinjection of In Vivo ASXL2-3xFlag. **(I)** Immunostaining of EZH2 10 weeks after microinjection of in vivo EZH2-3xFlag. **(J)** Mice were exposed to 11.7% oxygen for 10 weeks and injected with In Vivo ASXL2-3xFlag or In Vivo EZH2-3xFlag. The viability of sperm subjected to 11.7% oxygen for 10 weeks (n = 12). The sperm motility parameters, including progressive motility (n = 12), curvilinear velocity (n = 12), straightness (n = 12), straight-line velocity (n = 12), beat-cross frequency (n = 12), average path velocity (n = 12) and amplitude of lateral head displacement (n = 12), were examined with a sperm class analyzer system (CSA, Microptic S.L., Barcelona, Spain). **(K)** Acetic acid magenta staining was used to observe the entry of spermatogonia into oocytes, and the percentage of sperm that penetrated into oocytes was calculated (%) (n = 12). **(L)** Sperm flagellar abnormalities (%) were identified through Papanicolaou staining (n = 12). The data are presented as the means ± SDs; ns nonsignificant, ***P* < 0.01, ****P* < 0.001, *****P* < 0.0001; 2-way ANOVA followed by Tukey’s post hoc test (E-G, J-L).

**Supplementary Figure 6**

**
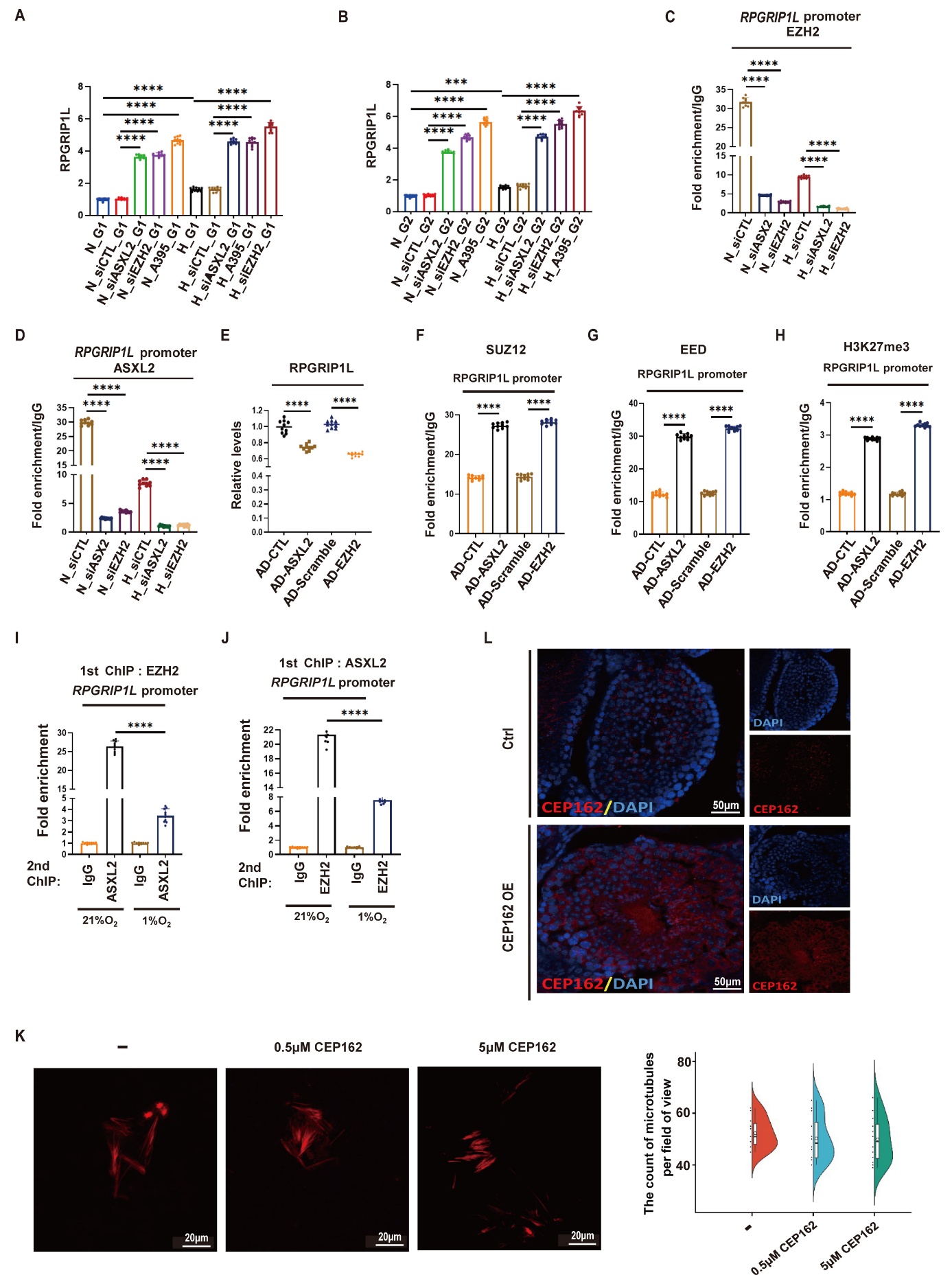
**

**Supplementary Figure 6. The ASXL2–EZH2 complex exerts inhibitory control over RPGRIP1L transcription.**

G1 and G2 cells deficient in ASXL2/EZH2 were subjected to 1% oxygen hypoxic conditions for 48 hours. qPCR was used to evaluate CEP162 mRNA levels in both control and ASXL2/EZH2-deficient cells, with a focus on **(A)** G1 (n = 10) and **(B)** G2 cells (n = 10). The mRNA expression in these cells was normalized to that of β-actin and reported as a ratio to the control group's expression. ChIP coupled with qPCR was used to examine the binding of **(C)** EZH2 (n = 10) and **(D)** ASXL2 (n = 10) to the CEP162 promoter in G2 cells treated with control, ASXL2 siRNA, or EZH2 siRNA. The overexpression of exogenous ASXL2/EZH2 in G2 cells was induced via adenoviral transfection. **(E)** qPCR was also used to assess CEP162 mRNA levels in the control group compared with the AD-ASXL2/EZH2 group in G2 cells, with normalization to β-actin and expression as a ratio to the control group (n = 10). ChIP‒qPCR analysis was performed to determine the occupancy of **(F)** SUZ12 (n = 10), **(G)** EED (n = 10), and **(H)** H3K27me3 (n = 10) on the CEP162 promoter in G2 cells treated with control, AD-ASXL2, or AD-EZH2. Sequential ChIP‒qPCR analysis was conducted to identify the colocalization of **(I)** ASXL2 (n = 10) and **(J)** EZH2 (n = 10) on the CEP162 promoter in G2 cell lines under both normoxic and hypoxic conditions. The specific ChIP antibodies used in each step are indicated in the chart titles and x-axis labels. **(K)** Microtubules were assembled from a mixture of rhodamine-labeled and unlabeled tubulin at a ratio of 1:9 in the presence of CEP162 at concentrations of 0.5 μM and 5 μM (n = 12). **(L)** Lentivirus-mediated in vivo delivery to the testis for the exogenous overexpression of CEP162. The data are presented as the means ± SDs, *****P* < 0.0001, 1-way ANOVA followed by Tukey’s post hoc test (I, and J); *****P* < 0.0001; 2-way ANOVA followed by Tukey’s post hoc test (A-H).

**Supplementary Figure 7**


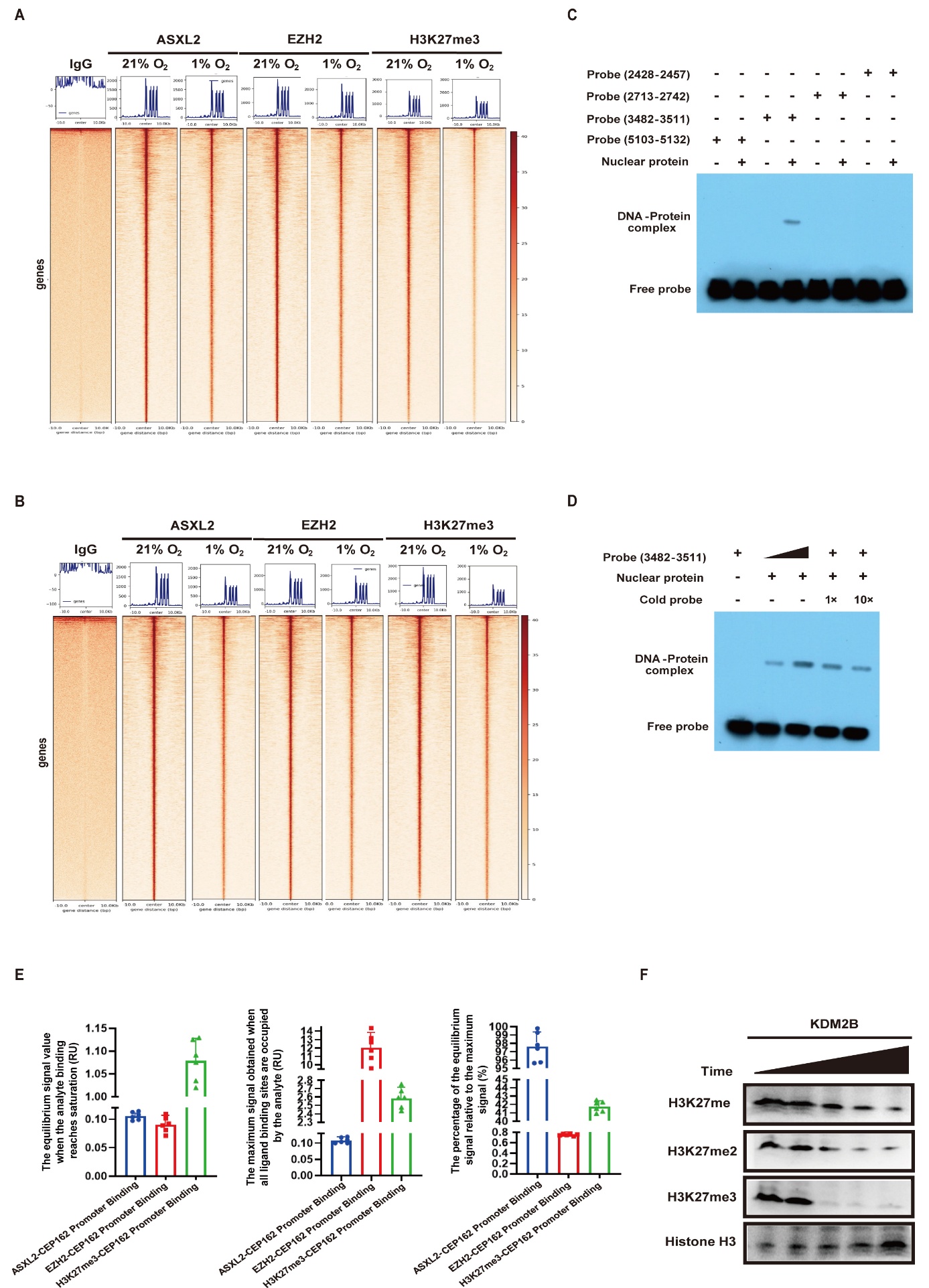


**Supplementary Figure 7. Supplementary Figure 7. ASXL2–EZH2 Interaction at the CEP162 Promoter (3482–3511 bp) for H3K27me3 Catalysis.**

The heatmap analysis presents the Cut&Tag sequencing data for the ASXL2, EZH2, and H3K27me3 peaks within a 6 kb region centered on their respective transcription start sites. These data are displayed for two distinct cell lines, **(A)** G1 cells and **(B)** G2 cells, highlighting the distribution of these epigenetic marks in relation to the transcriptional activity of ASXL2, EZH2, and H3K27me3. Note: The genes represent the number of genes to which ASXL2, EZH2, and H3K27me3 bind. Using deepTools (v3.3.1) to generate a heatmap[26], the transcription start site (TSS) was annotated with normalized read density counts (RPKM) from Cut&Tag Seq experiments, which were calculated as Read Per Kilobase per Million mapped reads (RPKM), which represents the number of sequences contained on every 1 kb exon sequence and the number of sequences enriched in exons per million aligned sequences), with a margin of ± 500 bp. MACS2 was used to identify ASXL2, EZH2, and H3K27me3 peaks [27]. **(C)** EMSA analysis was used to assess the binding of nuclear proteins to four distinct probes within the promoter region of CEP162, spanning sequences ranging from 2428–2457 bp, 2713–2742 bp, 3482–3511 bp, and 5103–5132 bp. **(D)** EMSA was used to evaluate the binding of nuclear proteins to the CEP162 promoter probe spanning 3482–3511 bp, as well as to a cold probe. **(E)** The graphs depict the binding kinetics of the CEP162 promoter segment (3482–3511 bp) with ASXL2, EZH2, and H3K27me3, showing equilibrium (Req), maximum signal (Rmax), and saturation level (Req/Rmax%). The saturation level indicates the percentage of maximum binding capacity achieved under the given conditions (n = 6). **(F)** Western blot analysis was performed using antibodies against H3K27me, H3K27me2, H3K27me3, and total histone H3 to monitor the methylation status of lysine 27 over time, with KDM2B-mediated demethylation assessed at specific time points (0, 10, 20, 40, and 80 minutes).

**Supplementary Figure 8a**

**
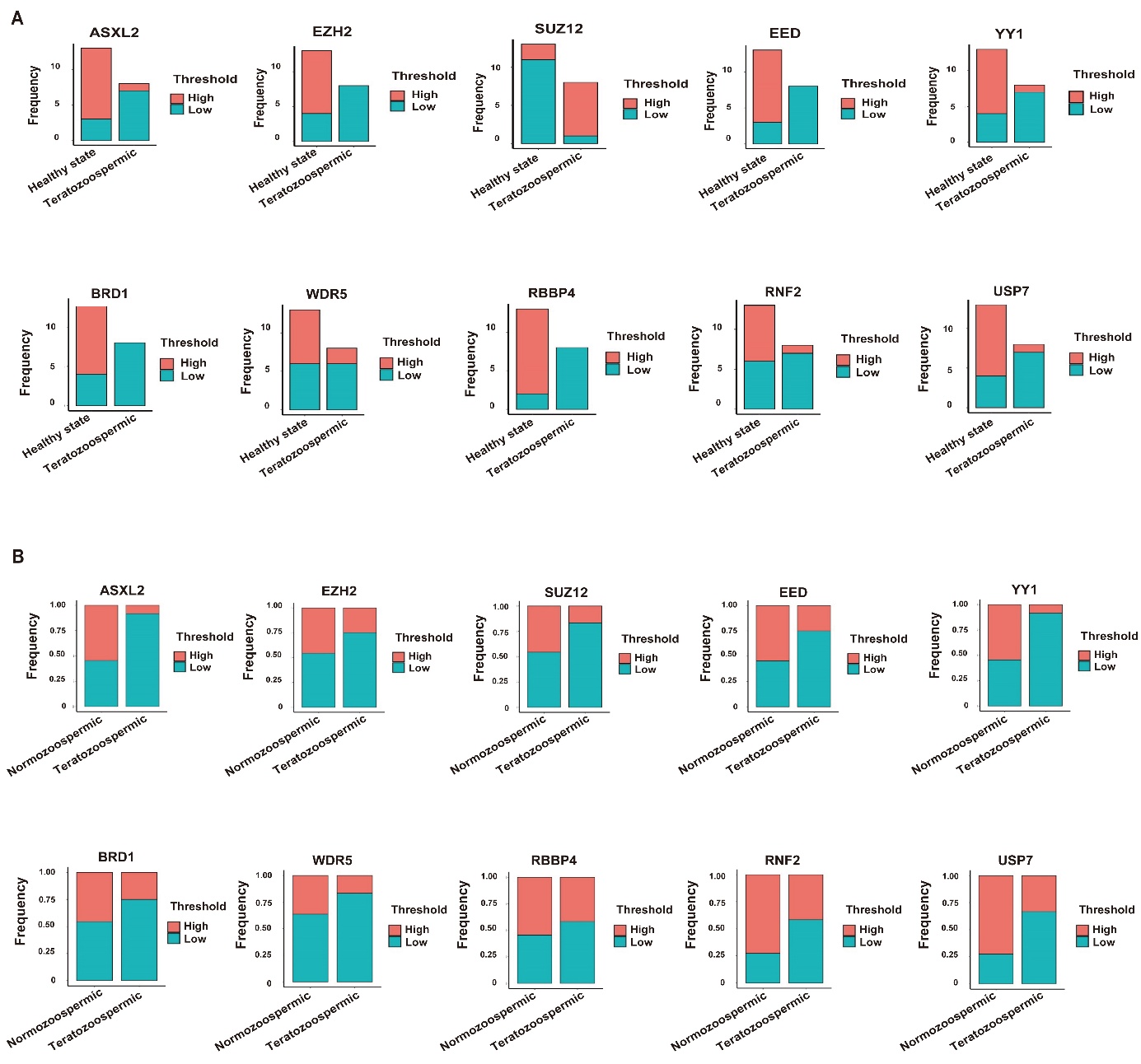
**

**Supplementary Figure 8a (A & B). Analysis of median gene expression data for patients with teratozoospermia in the GEO dataset.**

Patients were categorized on the basis of high or low gene expression associated with **(A)** teratozoospermia (using Affymetrix platform data from GSE6872) (n = 13 for Healthy state, n = 8 for Teratozoospermic) and **(B)** teratozoospermia (Illumina platform data from GSE6967 and GSE6968) (n = 9 for Normozoospermic, n = 14 for Teratozoospermic). Genes were stratified as highly or poorly expressed on the basis of the average expression values across all samples.

**Supplementary Figure 8b**


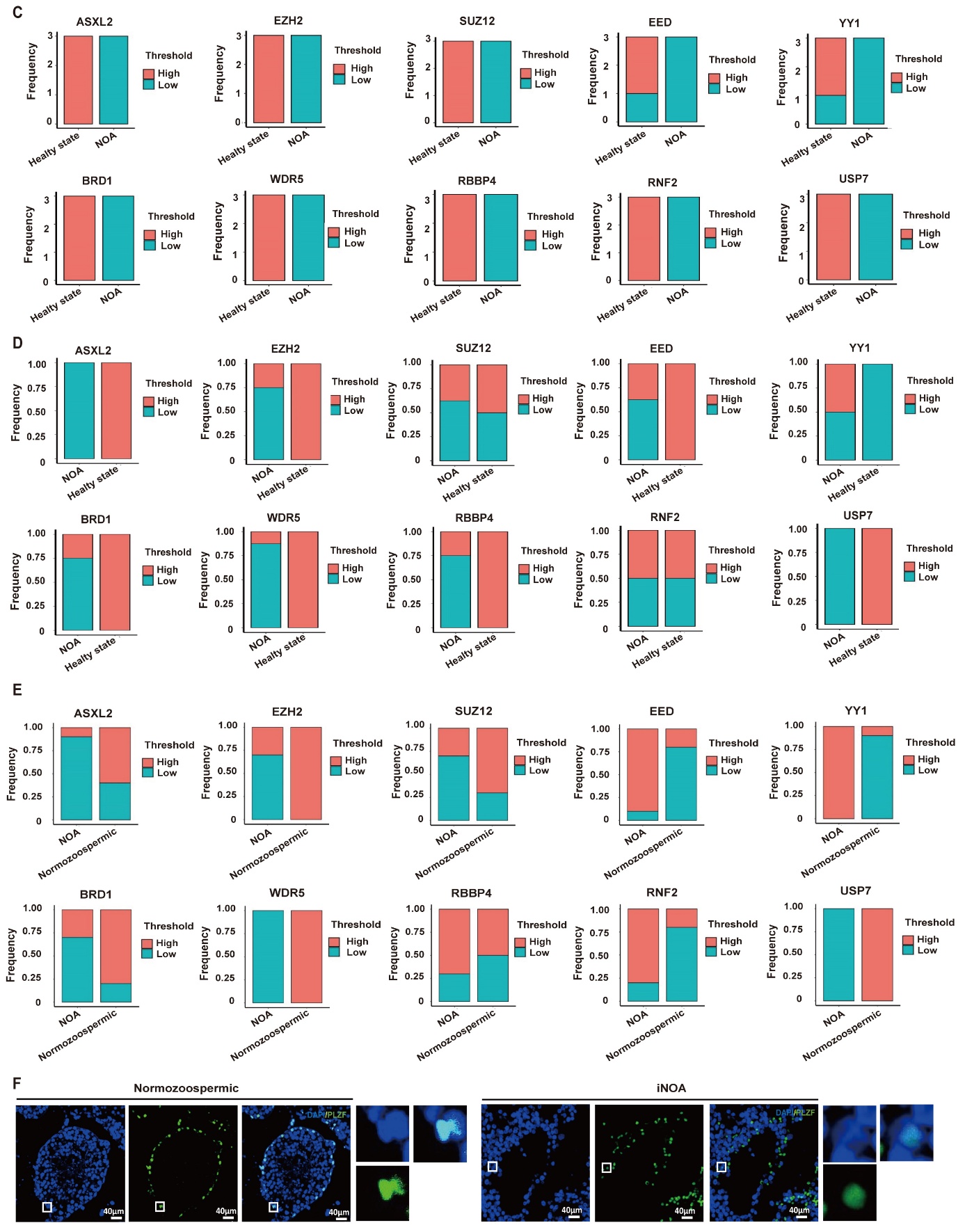


**Supplementary Figure 8b (C-F). Analysis of median gene expression data for patients with idiopathic non-obstructive azoospermia (iNOA) in the GEO dataset.**

Patients were categorized on the basis of high or low gene expression associated with **(C)** NOA (GSE190752, with expression levels standardized using FPKM) (n = 3 for Healthy state, n = 3 for NOA), **(D)** NOA (GSE216907, employing TPM for gene expression normalization) (n = 2 for Healthy state, n = 8 for NOA), and **(E)** NOA (Agilent PCR array platform data from GSE145467) (n = 15 for Healthy state, n = 5 for NOA). Genes were stratified as highly or poorly expressed on the basis of the average expression values across all samples. **(F)** Immunofluorescence staining of PLZF in seminiferous tubules from normozoospermic individuals and patients with idiopathic non-obstructive azoospermia (iNOA). Nuclei were counterstained with DAPI. Scale bar, 40 μm.

**Supplementary Figure 8c**


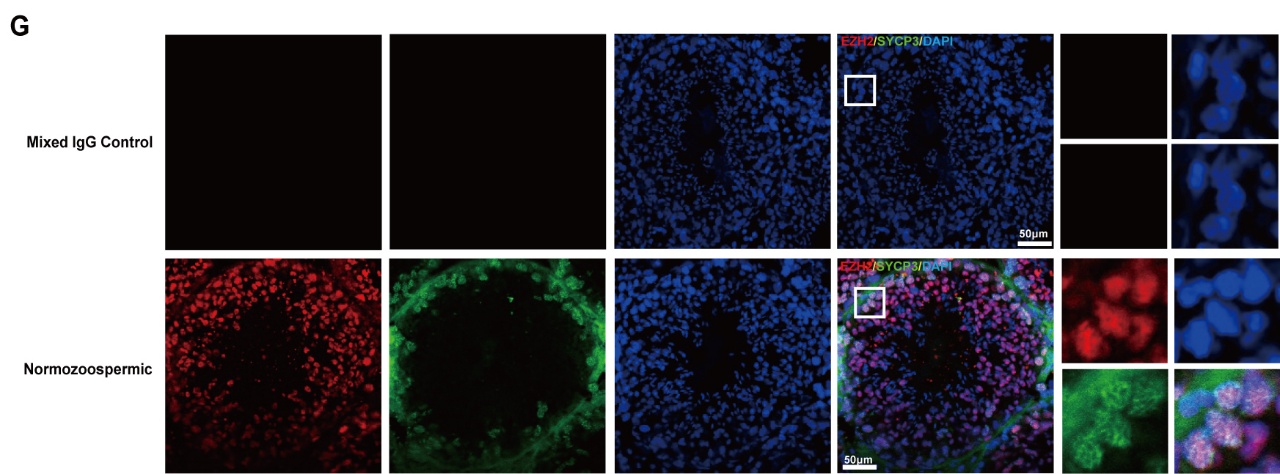


**Supplementary Figure 8c Co-localization of EZH2 and SYCP3 in seminiferous tubules of normozoospermic controls. (G)** Immunofluorescence analysis of EZH2 (red) and SYCP3 (green) co-localization in seminiferous tubules of the normozoospermic group. DAPI (blue) stains nuclei.
