# Supplementary material for: Chronic Hypoxia Disrupts Spermatogenesis Through ASXL2–EZH2–Mediated Microtubule Destabilization
Source: Adv Sci (Weinh). 2026 Mar 4;13(26):e01266. doi: 10.1002/advs.202501266 (PMC13159132; doi:10.1002/advs.202501266)
Supplement: Supplementary file 5 — Supporting File 5: advs74564‐sup‐0005‐Data.zip [file ADVS-13-e01266-s001.zip › advs74564-sup-0005-Data/Supplementary_Methods_DoubletFinder_Analysis.docx]

Sample1 (CH_Testis 1)

#质控


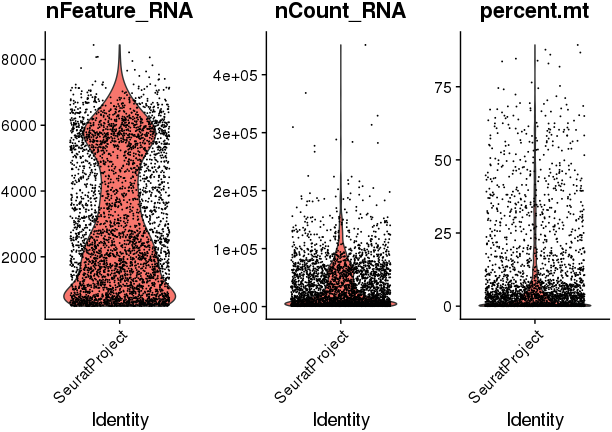


ch1 <- subset(ch1, subset = nFeature_RNA > 300 & percent.mt < 20 & nFeature_RNA < 7000 & nCount_RNA > 1000)

#双细胞去除

easy_clustering <- function(object = object){

object <- NormalizeData(object)

object <- FindVariableFeatures(object, nfeatures = 2000)

object <- ScaleData(object, features = VariableFeatures(object))

object <- RunPCA(object, features = VariableFeatures(object = object), npcs = 50)

object <- FindNeighbors(object, dims = 1:30)

object <- FindClusters(object, resolution = c(0.5,1,2))

object <- RunUMAP(object, dims = 1:30)

return(object)

}

data = easy_clustering(ch1)

sweep.res.list <- paramSweep_v3(data, PCs = 1:30, sct = F)

sweep.stats <- summarizeSweep(sweep.res.list, GT = FALSE)

bcmvn <- find.pK(sweep.stats)

pK_bcmvn <- bcmvn$pK[which.max(bcmvn$BCmetric)] %>% as.character() %>% as.numeric()

DoubletRate = ncol(data)*8*1e-6

nExp_poi <- round(DoubletRate*ncol(data))

seu_colon <- doubletFinder_v3(data, PCs = 1:30, pN = 0.25, pK = 0.01, nExp = nExp_poi, reuse.pANN = FALSE, sct = FALSE)

seu_colon$doublet.class <- seu_colon[[paste0("DF.classifications_0.25_0.01_",nExp_poi)]]

seu_colon[[paste0("DF.classifications_0.25_0.01_",nExp_poi)]] <- NULL

pann <- grep(pattern="^pANN", x=names(seu_colon@meta.data), value=TRUE)

seu_colon$pANN <- seu_colon[[pann]]

seu_colon[[pann]] <- NULL

DimPlot(seu_colon, reduction = "umap", group.by = "doublet.class", cols = c("#D51F26", "#272E6A"))


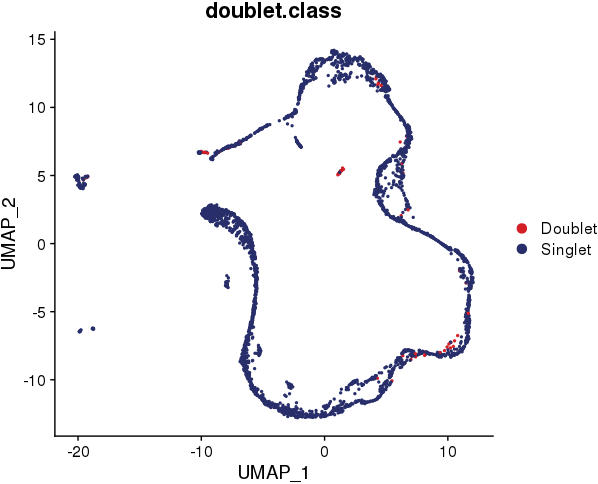


seu_colon <- subset(seu_colon, subset = doublet.class != "Doublet")

seu_colon <- DietSeurat(seu_colon, counts=TRUE, data=TRUE, scale.data=FALSE, assays="RNA")

ch1 = seu_colon

Sample2 (CH_Testis 2)

#质控


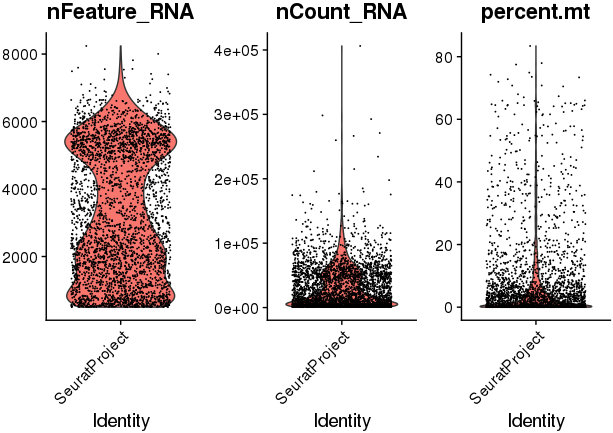


ch2 <- subset(ch2, subset = nFeature_RNA > 300 & percent.mt < 20 & nFeature_RNA < 7000 & nCount_RNA > 1000)

#双细胞去除

easy_clustering <- function(object = object){

object <- NormalizeData(object)

object <- FindVariableFeatures(object, nfeatures = 2000)

object <- ScaleData(object, features = VariableFeatures(object))

object <- RunPCA(object, features = VariableFeatures(object = object), npcs = 50)

object <- FindNeighbors(object, dims = 1:30)

object <- FindClusters(object, resolution = c(0.5,1,2))

object <- RunUMAP(object, dims = 1:30)

return(object)

}

data = easy_clustering(ch2)

sweep.res.list <- paramSweep_v3(data, PCs = 1:30, sct = F)

sweep.stats <- summarizeSweep(sweep.res.list, GT = FALSE)

bcmvn <- find.pK(sweep.stats)

pK_bcmvn <- bcmvn$pK[which.max(bcmvn$BCmetric)] %>% as.character() %>% as.numeric()

DoubletRate = ncol(data)*8*1e-6

nExp_poi <- round(DoubletRate*ncol(data))

seu_colon <- doubletFinder_v3(data, PCs = 1:30, pN = 0.25, pK = 0.03, nExp = nExp_poi, reuse.pANN = FALSE, sct = FALSE)

seu_colon$doublet.class <- seu_colon[[paste0("DF.classifications_0.25_0.03_",nExp_poi)]]

seu_colon[[paste0("DF.classifications_0.25_0.03_",nExp_poi)]] <- NULL

pann <- grep(pattern="^pANN", x=names(seu_colon@meta.data), value=TRUE)

seu_colon$pANN <- seu_colon[[pann]]

seu_colon[[pann]] <- NULL

DimPlot(seu_colon, reduction = "umap", group.by = "doublet.class", cols = c("#D51F26", "#272E6A"))


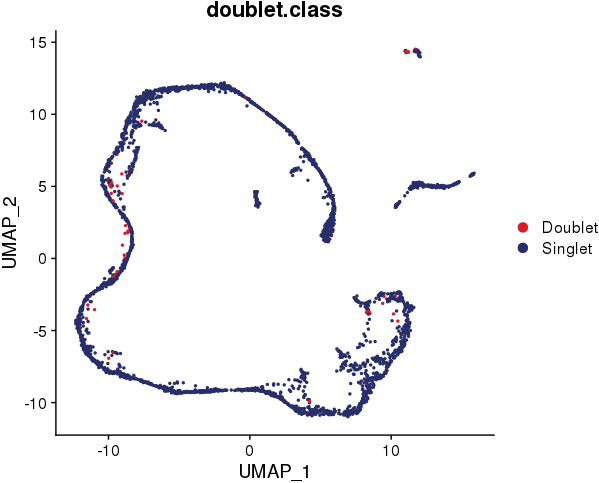


seu_colon <- subset(seu_colon, subset = doublet.class != "Doublet")

seu_colon <- DietSeurat(seu_colon, counts=TRUE, data=TRUE, scale.data=FALSE, assays="RNA")

ch2 = seu_colon

Sample3 (CH_Testis 3)

#质控


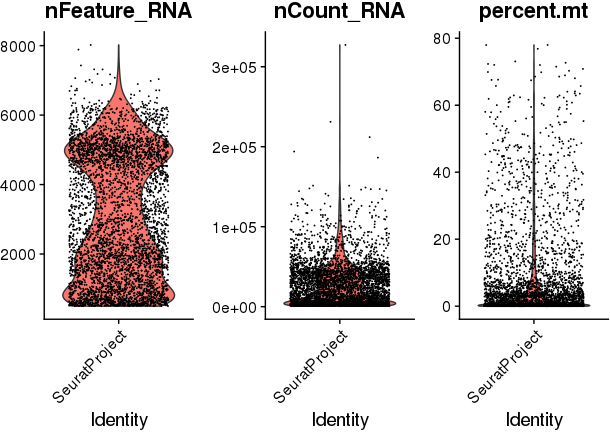


ch3 <- subset(ch3, subset = nFeature_RNA > 300 & percent.mt < 20 & nFeature_RNA < 7000 & nCount_RNA > 1000)

#双细胞去除

easy_clustering <- function(object = object){

object <- NormalizeData(object)

object <- FindVariableFeatures(object, nfeatures = 2000)

object <- ScaleData(object, features = VariableFeatures(object))

object <- RunPCA(object, features = VariableFeatures(object = object), npcs = 50)

object <- FindNeighbors(object, dims = 1:30)

object <- FindClusters(object, resolution = c(0.5,1,2))

object <- RunUMAP(object, dims = 1:30)

return(object)

}

data = easy_clustering(ch3)

sweep.res.list <- paramSweep_v3(data, PCs = 1:30, sct = F)

sweep.stats <- summarizeSweep(sweep.res.list, GT = FALSE)

bcmvn <- find.pK(sweep.stats)

pK_bcmvn <- bcmvn$pK[which.max(bcmvn$BCmetric)] %>% as.character() %>% as.numeric()

DoubletRate = ncol(data)*8*1e-6

nExp_poi <- round(DoubletRate*ncol(data))

seu_colon <- doubletFinder_v3(data, PCs = 1:30, pN = 0.25, pK = 0.09, nExp = nExp_poi, reuse.pANN = FALSE, sct = FALSE)

seu_colon$doublet.class <- seu_colon[[paste0("DF.classifications_0.25_0.09_",nExp_poi)]]

seu_colon[[paste0("DF.classifications_0.25_0.09_",nExp_poi)]] <- NULL

pann <- grep(pattern="^pANN", x=names(seu_colon@meta.data), value=TRUE)

seu_colon$pANN <- seu_colon[[pann]]

seu_colon[[pann]] <- NULL

DimPlot(seu_colon, reduction = "umap", group.by = "doublet.class", cols = c("#D51F26", "#272E6A"))


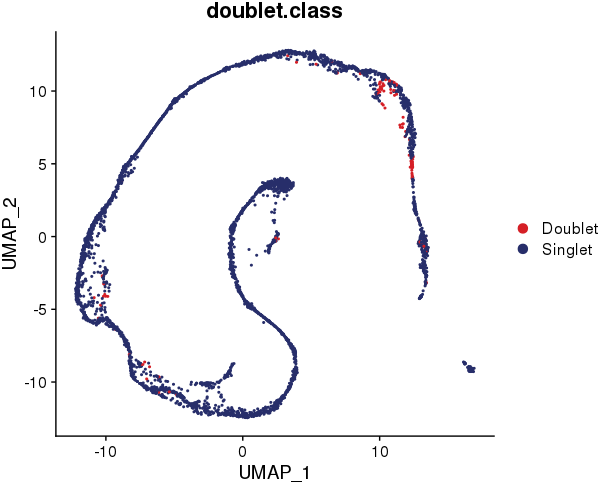


seu_colon <- subset(seu_colon, subset = doublet.class != "Doublet")

seu_colon <- DietSeurat(seu_colon, counts=TRUE, data=TRUE, scale.data=FALSE, assays="RNA")

ch3 = seu_colon

Sample4 (PC_Testis 1)

#质控


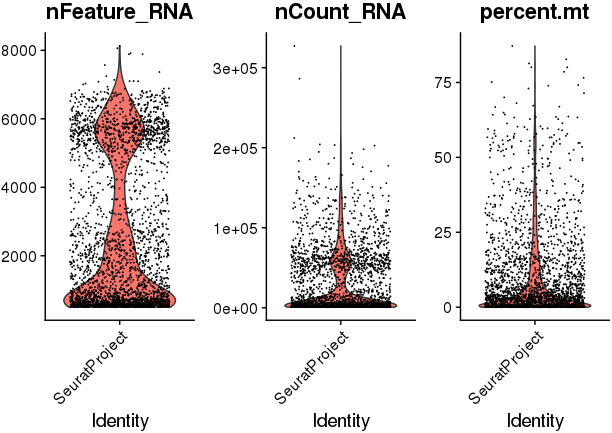


pc1 <- subset(pc1, subset = nFeature_RNA > 300 & percent.mt < 20 & nFeature_RNA < 7000 & nCount_RNA > 1000)

#双细胞去除

easy_clustering <- function(object = object){

object <- NormalizeData(object)

object <- FindVariableFeatures(object, nfeatures = 2000)

object <- ScaleData(object, features = VariableFeatures(object))

object <- RunPCA(object, features = VariableFeatures(object = object), npcs = 50)

object <- FindNeighbors(object, dims = 1:30)

object <- FindClusters(object, resolution = c(0.5,1,2))

object <- RunUMAP(object, dims = 1:30)

return(object)

}

data = easy_clustering(pc1)

sweep.res.list <- paramSweep_v3(data, PCs = 1:30, sct = F)

sweep.stats <- summarizeSweep(sweep.res.list, GT = FALSE)

bcmvn <- find.pK(sweep.stats)

pK_bcmvn <- bcmvn$pK[which.max(bcmvn$BCmetric)] %>% as.character() %>% as.numeric()

DoubletRate = ncol(data)*8*1e-6

nExp_poi <- round(DoubletRate*ncol(data))

seu_colon <- doubletFinder_v3(data, PCs = 1:30, pN = 0.25, pK = 0.28, nExp = nExp_poi, reuse.pANN = FALSE, sct = FALSE)

seu_colon$doublet.class <- seu_colon[[paste0("DF.classifications_0.25_0.28_",nExp_poi)]]

seu_colon[[paste0("DF.classifications_0.25_0.28_",nExp_poi)]] <- NULL

pann <- grep(pattern="^pANN", x=names(seu_colon@meta.data), value=TRUE)

seu_colon$pANN <- seu_colon[[pann]]

seu_colon[[pann]] <- NULL

DimPlot(seu_colon, reduction = "umap", group.by = "doublet.class", cols = c("#D51F26", "#272E6A"))


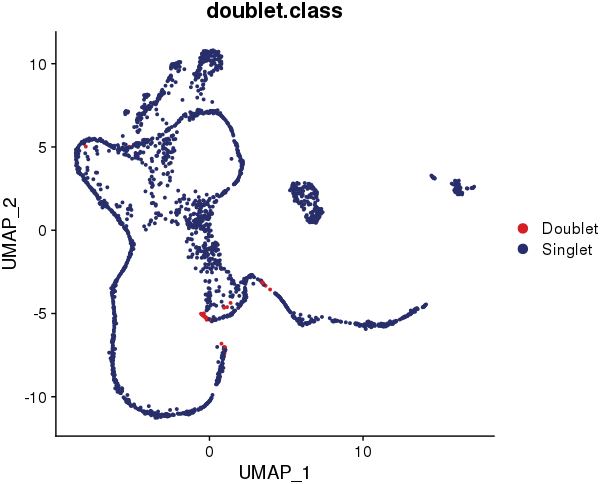


seu_colon <- subset(seu_colon, subset = doublet.class != "Doublet")

seu_colon <- DietSeurat(seu_colon, counts=TRUE, data=TRUE, scale.data=FALSE, assays="RNA")

pc1 = seu_colon

Sample5 (PC_Testis 2)

#质控


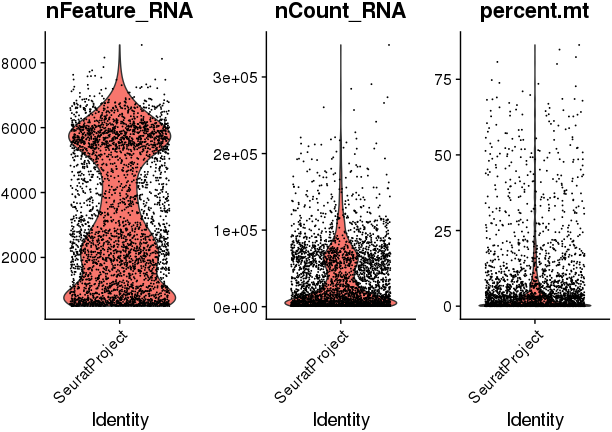


pc2 <- subset(pc2, subset = nFeature_RNA > 300 & percent.mt < 20 & nFeature_RNA < 7000 & nCount_RNA > 1000)

#双细胞去除

easy_clustering <- function(object = object){

object <- NormalizeData(object)

object <- FindVariableFeatures(object, nfeatures = 2000)

object <- ScaleData(object, features = VariableFeatures(object))

object <- RunPCA(object, features = VariableFeatures(object = object), npcs = 50)

object <- FindNeighbors(object, dims = 1:30)

object <- FindClusters(object, resolution = c(0.5,1,2))

object <- RunUMAP(object, dims = 1:30)

return(object)

}

data = easy_clustering(pc2)

sweep.res.list <- paramSweep_v3(data, PCs = 1:30, sct = F)

sweep.stats <- summarizeSweep(sweep.res.list, GT = FALSE)

bcmvn <- find.pK(sweep.stats)

pK_bcmvn <- bcmvn$pK[which.max(bcmvn$BCmetric)] %>% as.character() %>% as.numeric()

DoubletRate = ncol(data)*8*1e-6

nExp_poi <- round(DoubletRate*ncol(data))

seu_colon <- doubletFinder_v3(data, PCs = 1:30, pN = 0.25, pK = 0.005, nExp = nExp_poi, reuse.pANN = FALSE, sct = FALSE)

seu_colon$doublet.class <- seu_colon[[paste0("DF.classifications_0.25_0.005_",nExp_poi)]]

seu_colon[[paste0("DF.classifications_0.25_0.005_",nExp_poi)]] <- NULL

pann <- grep(pattern="^pANN", x=names(seu_colon@meta.data), value=TRUE)

seu_colon$pANN <- seu_colon[[pann]]

seu_colon[[pann]] <- NULL

DimPlot(seu_colon, reduction = "umap", group.by = "doublet.class", cols = c("#D51F26", "#272E6A"))


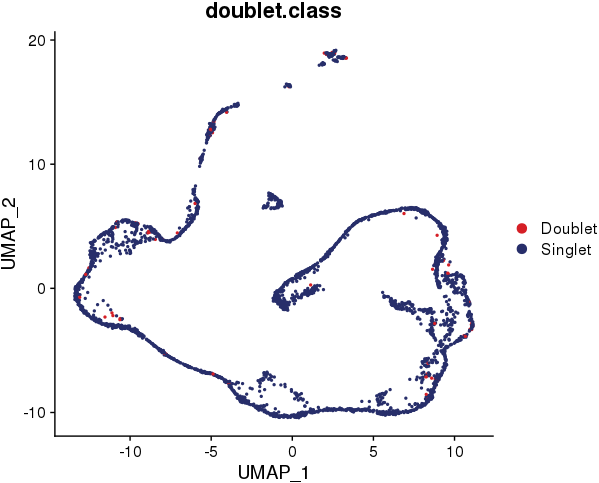


seu_colon <- subset(seu_colon, subset = doublet.class != "Doublet")

seu_colon <- DietSeurat(seu_colon, counts=TRUE, data=TRUE, scale.data=FALSE, assays="RNA")

pc2 = seu_colon

Sample6 (PC_Testis 3)

#质控


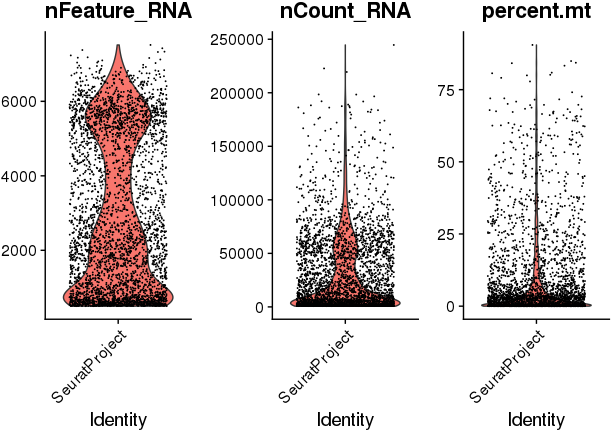


pc3 <- subset(pc3, subset = nFeature_RNA > 300 & percent.mt < 20 & nFeature_RNA < 7000 & nCount_RNA > 1000)

#双细胞去除

easy_clustering <- function(object = object){

object <- NormalizeData(object)

object <- FindVariableFeatures(object, nfeatures = 2000)

object <- ScaleData(object, features = VariableFeatures(object))

object <- RunPCA(object, features = VariableFeatures(object = object), npcs = 50)

object <- FindNeighbors(object, dims = 1:30)

object <- FindClusters(object, resolution = c(0.5,1,2))

object <- RunUMAP(object, dims = 1:30)

return(object)

}

data = easy_clustering(pc3)

sweep.res.list <- paramSweep_v3(data, PCs = 1:30, sct = F)

sweep.stats <- summarizeSweep(sweep.res.list, GT = FALSE)

bcmvn <- find.pK(sweep.stats)

pK_bcmvn <- bcmvn$pK[which.max(bcmvn$BCmetric)] %>% as.character() %>% as.numeric()

DoubletRate = ncol(data)*8*1e-6

nExp_poi <- round(DoubletRate*ncol(data))

seu_colon <- doubletFinder_v3(data, PCs = 1:30, pN = 0.25, pK = 0.005, nExp = nExp_poi, reuse.pANN = FALSE, sct = FALSE)

seu_colon$doublet.class <- seu_colon[[paste0("DF.classifications_0.25_0.005_",nExp_poi)]]

seu_colon[[paste0("DF.classifications_0.25_0.005_",nExp_poi)]] <- NULL

pann <- grep(pattern="^pANN", x=names(seu_colon@meta.data), value=TRUE)

seu_colon$pANN <- seu_colon[[pann]]

seu_colon[[pann]] <- NULL

DimPlot(seu_colon, reduction = "umap", group.by = "doublet.class", cols = c("#D51F26", "#272E6A"))


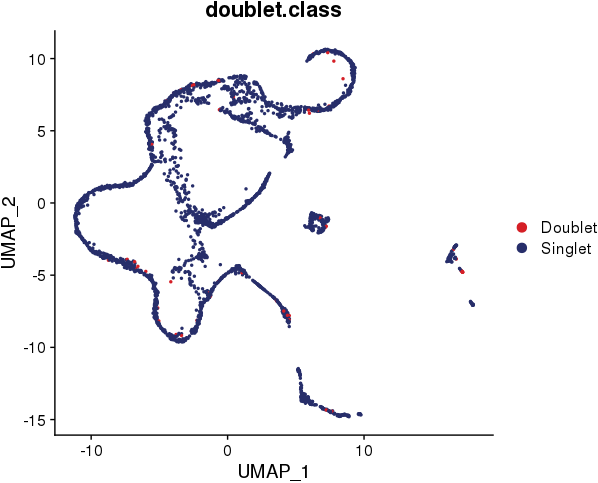


seu_colon <- subset(seu_colon, subset = doublet.class != "Doublet")

seu_colon <- DietSeurat(seu_colon, counts=TRUE, data=TRUE, scale.data=FALSE, assays="RNA")

pc3 = seu_colon
