# Supplementary figures and images for: Chronic Hypoxia Disrupts Spermatogenesis Through ASXL2–EZH2–Mediated Microtubule Destabilization
Source: Adv Sci (Weinh). 2026 Mar 4;13(26):e01266. doi: 10.1002/advs.202501266 (PMC13159132; doi:10.1002/advs.202501266)

Figure 2

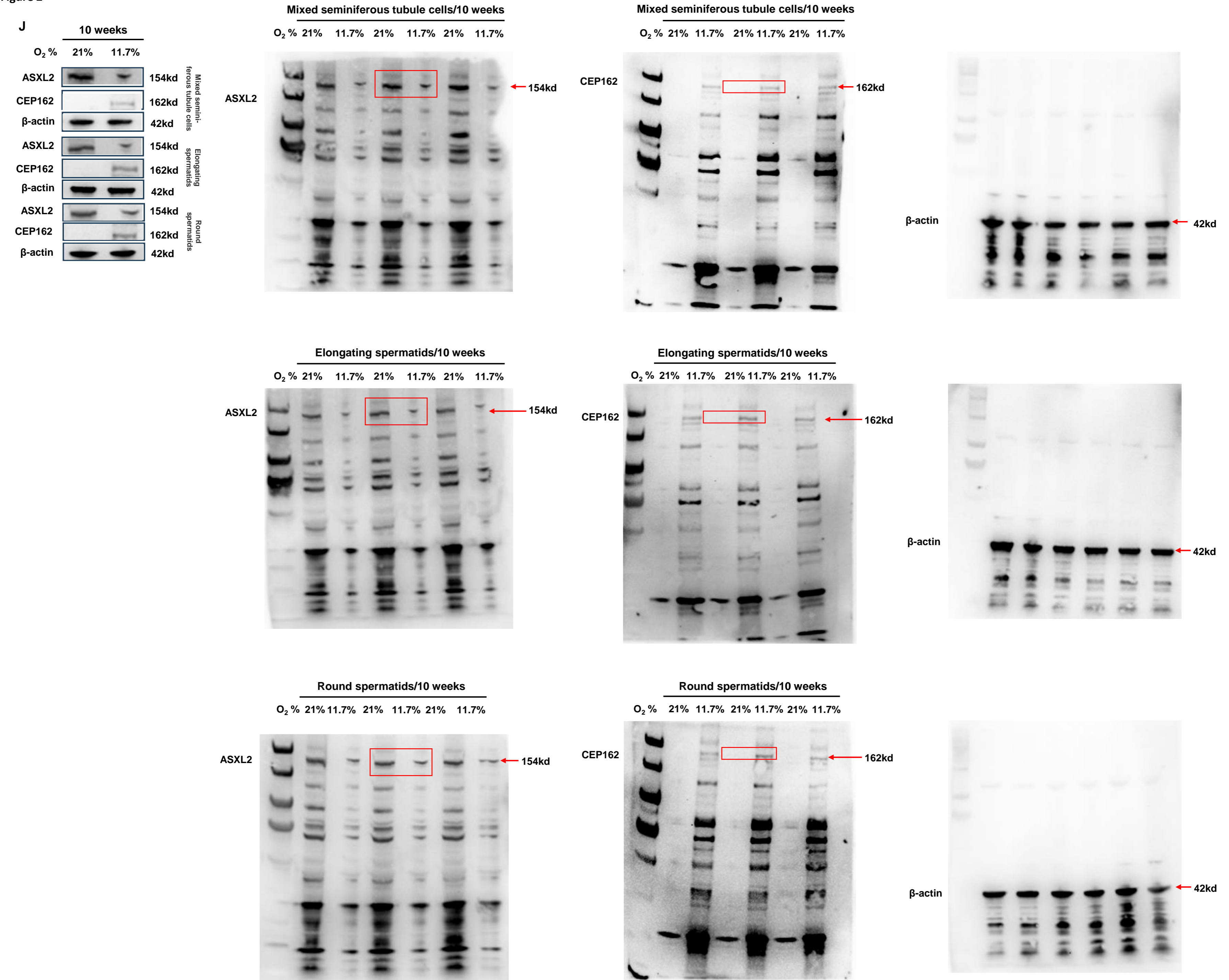

Figure 3

A

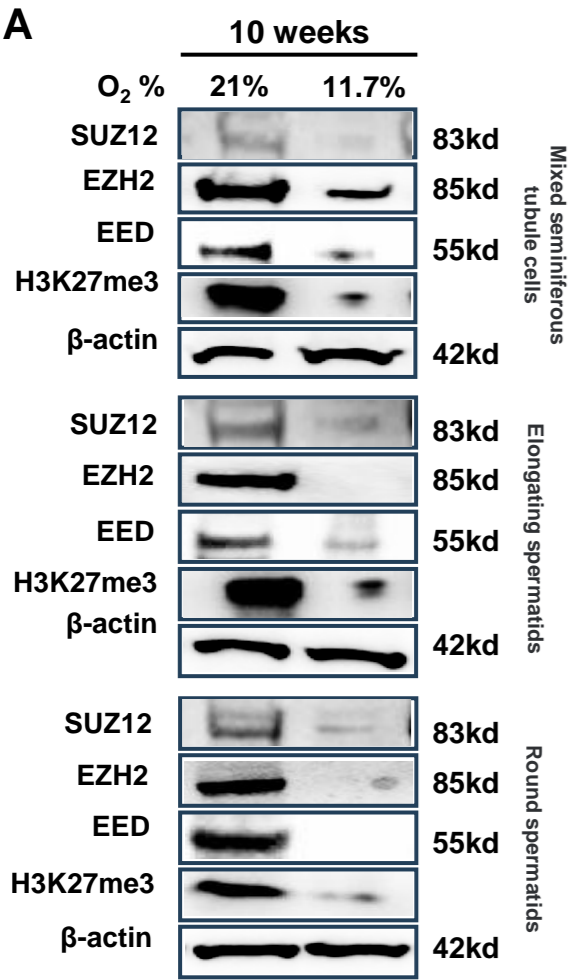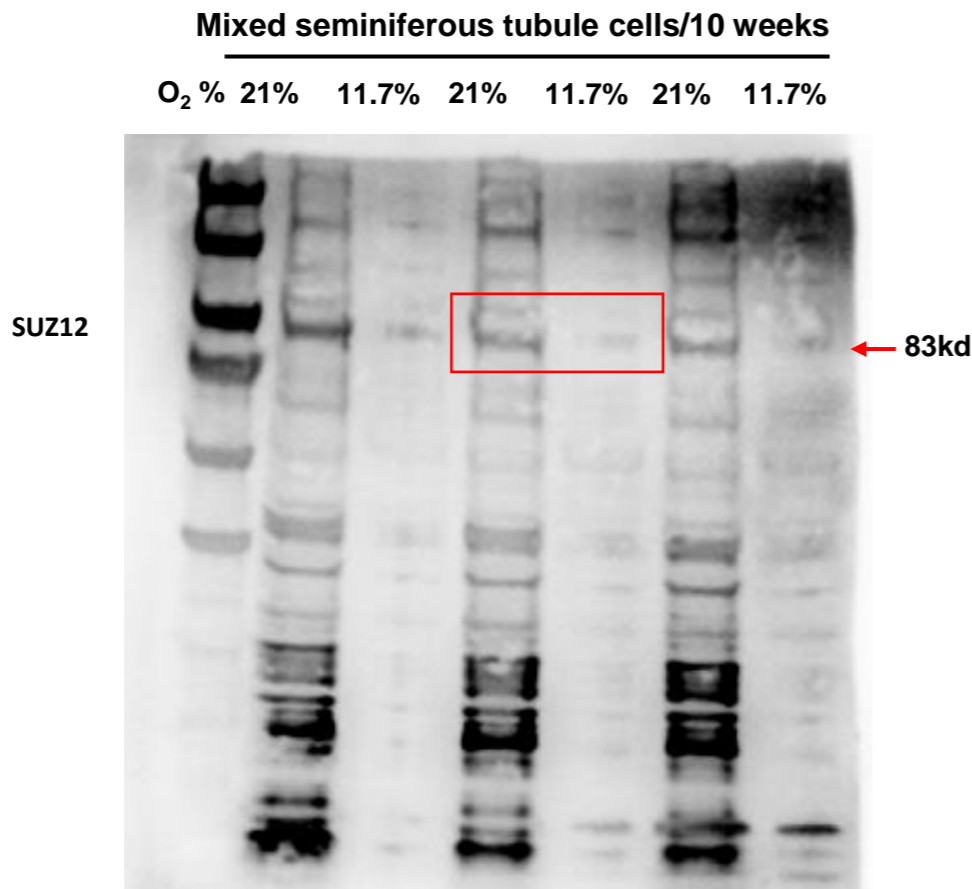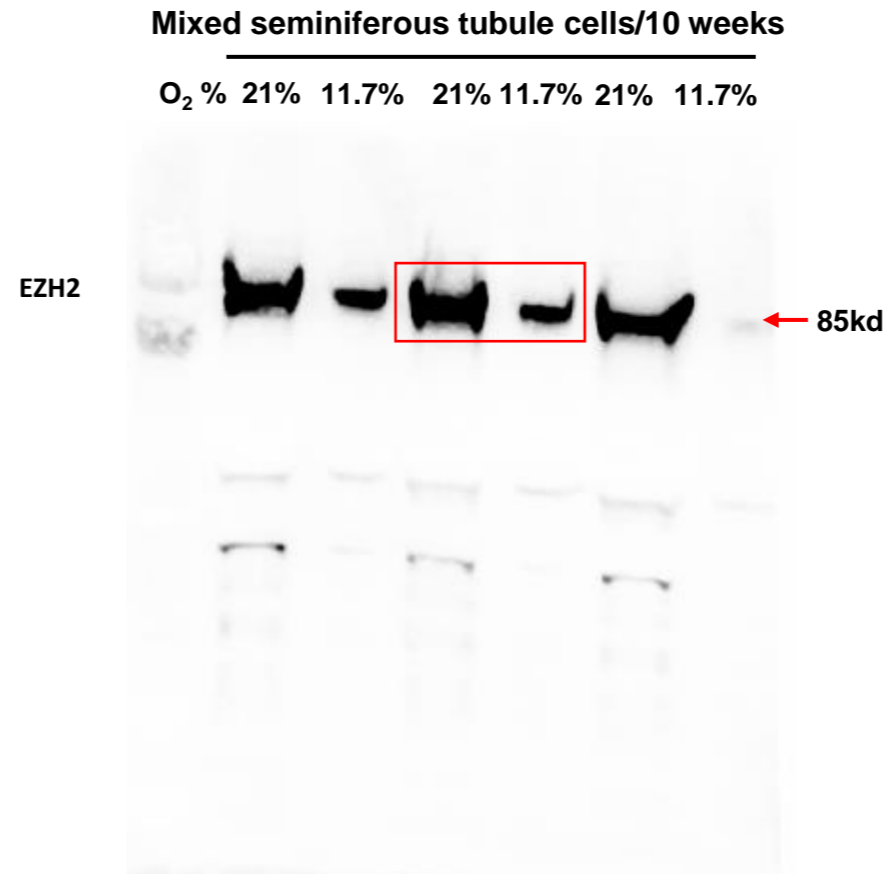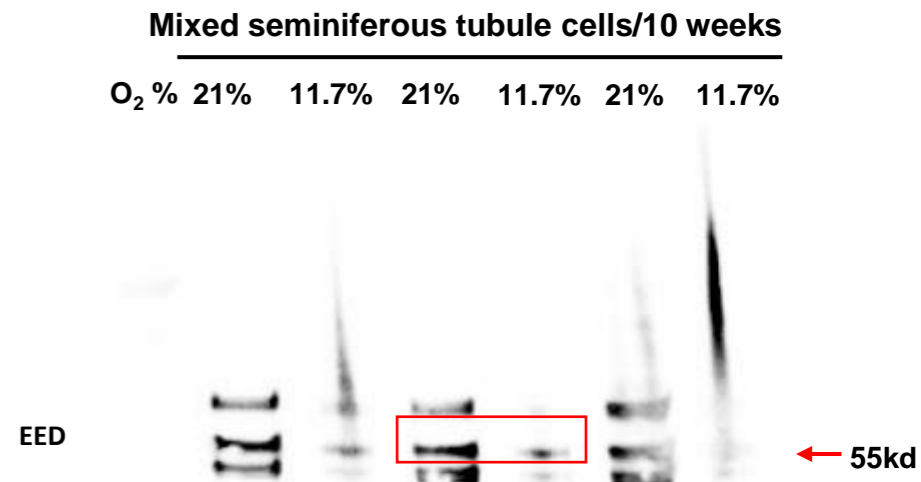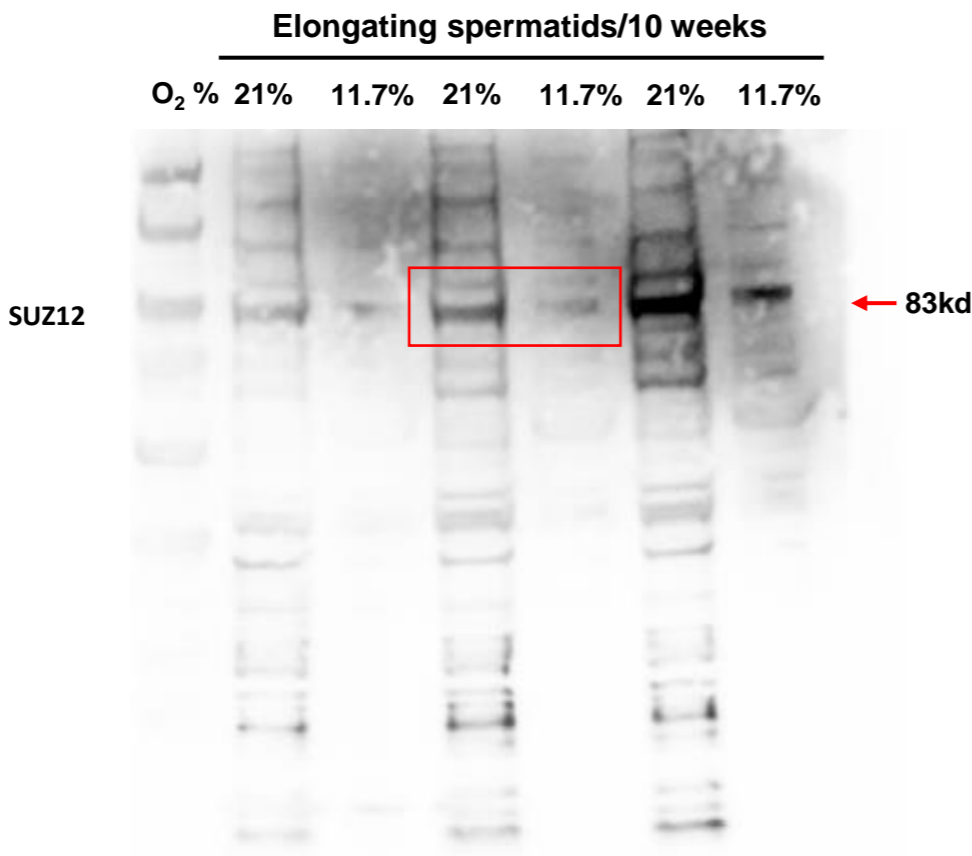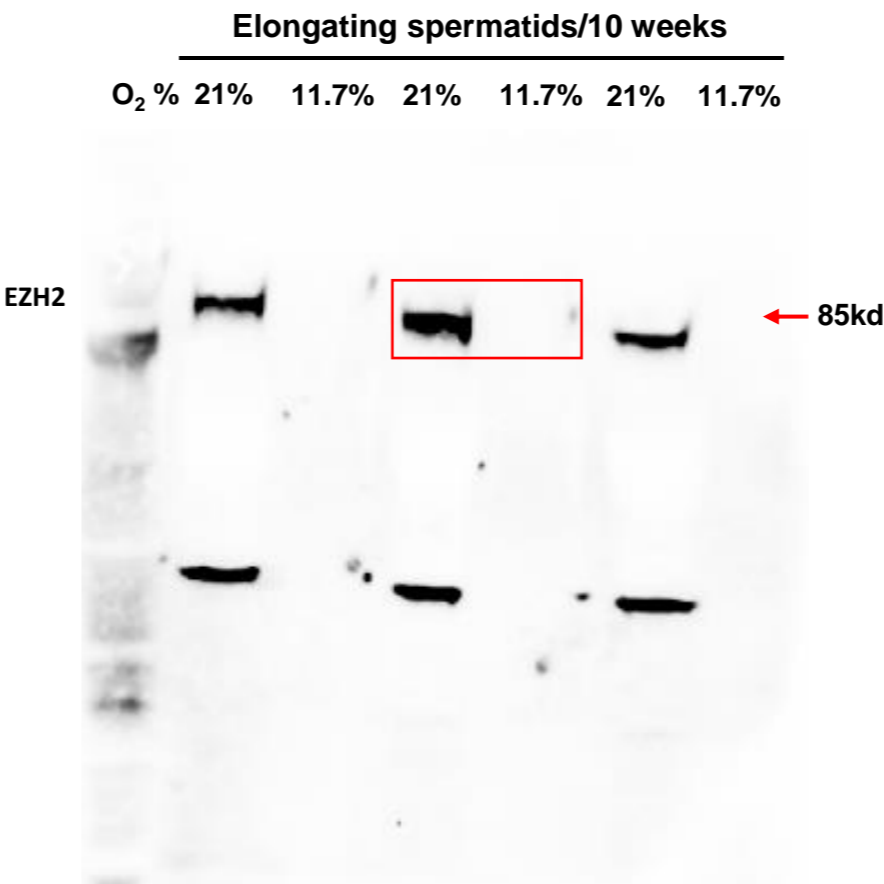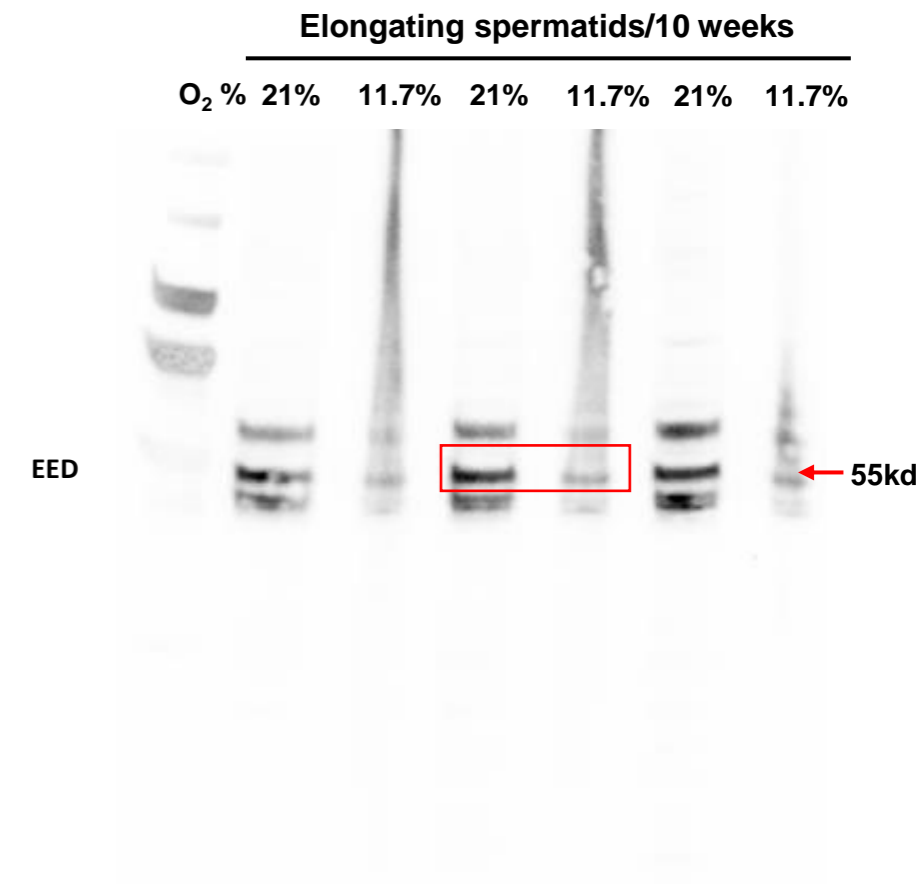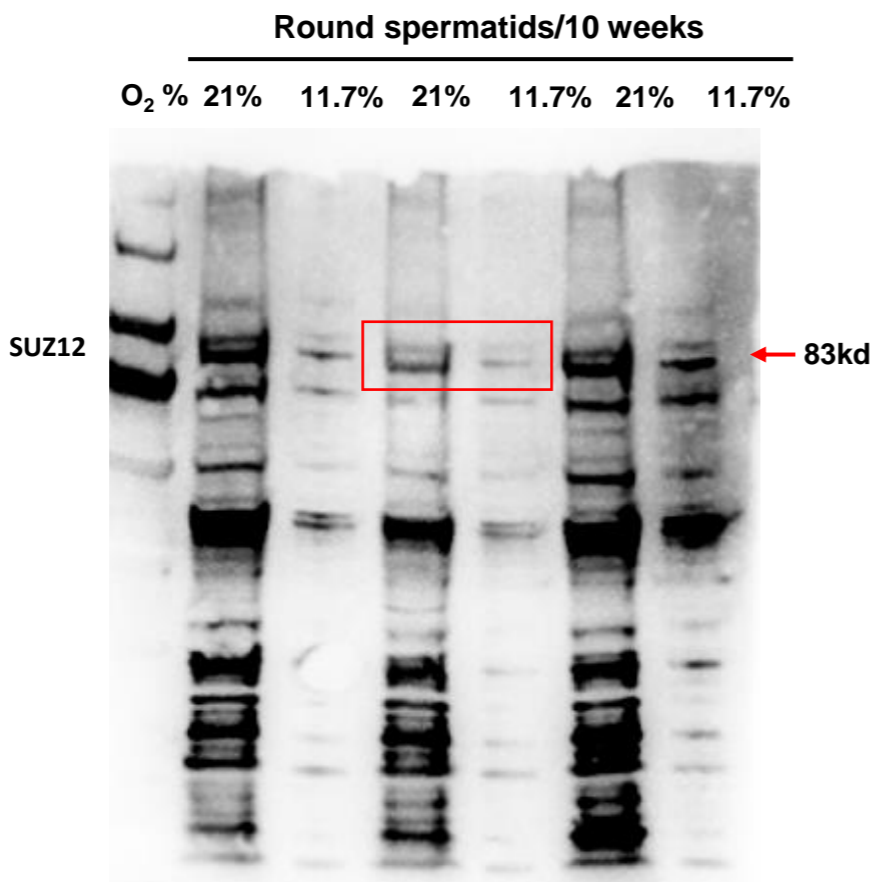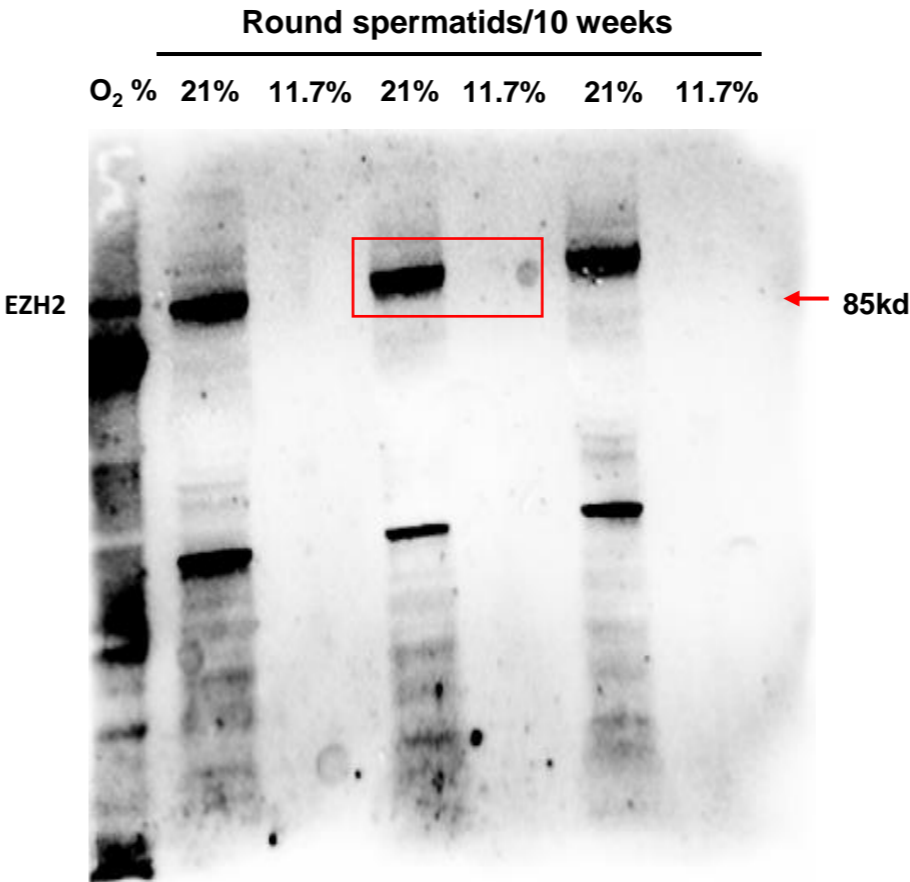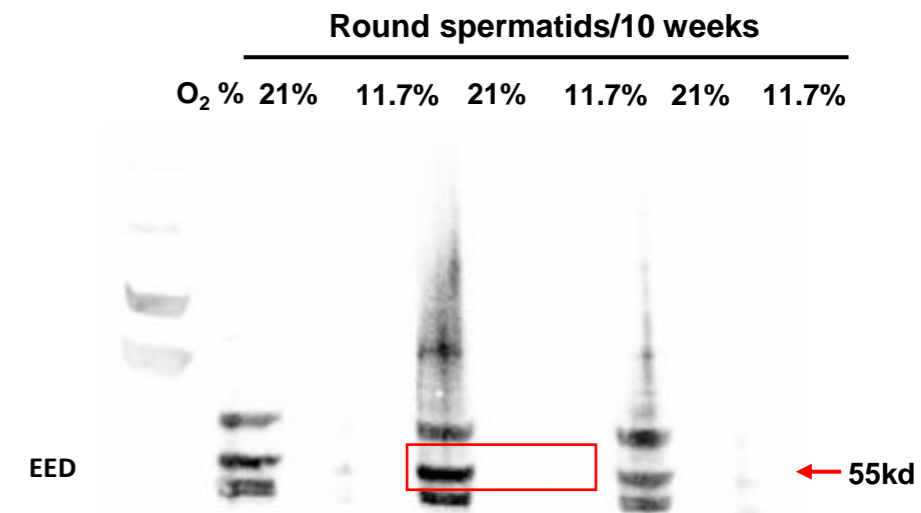

Figure 3

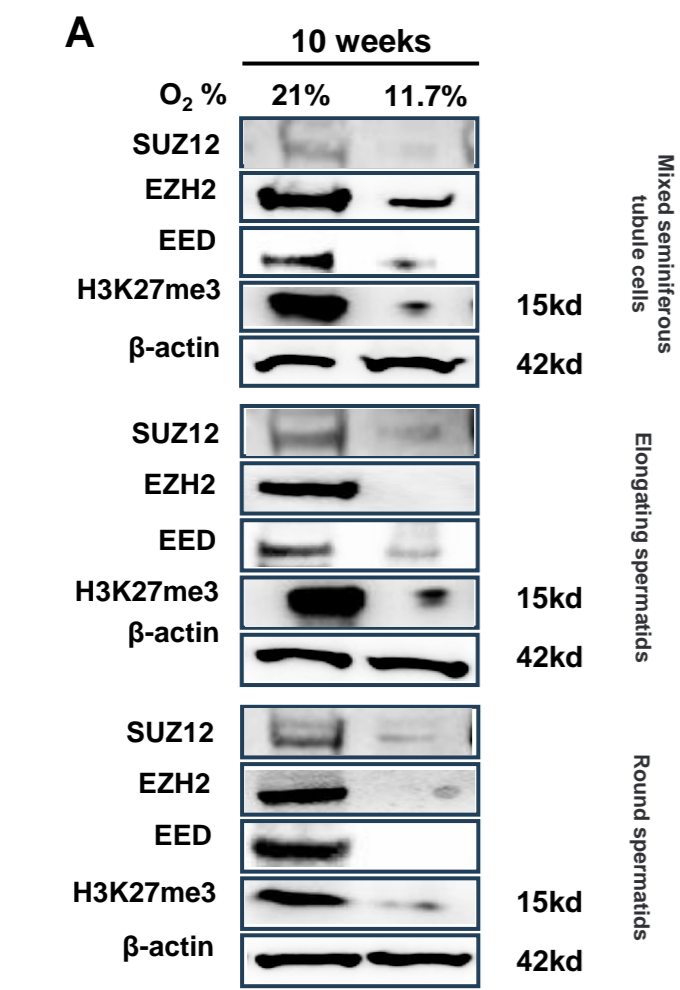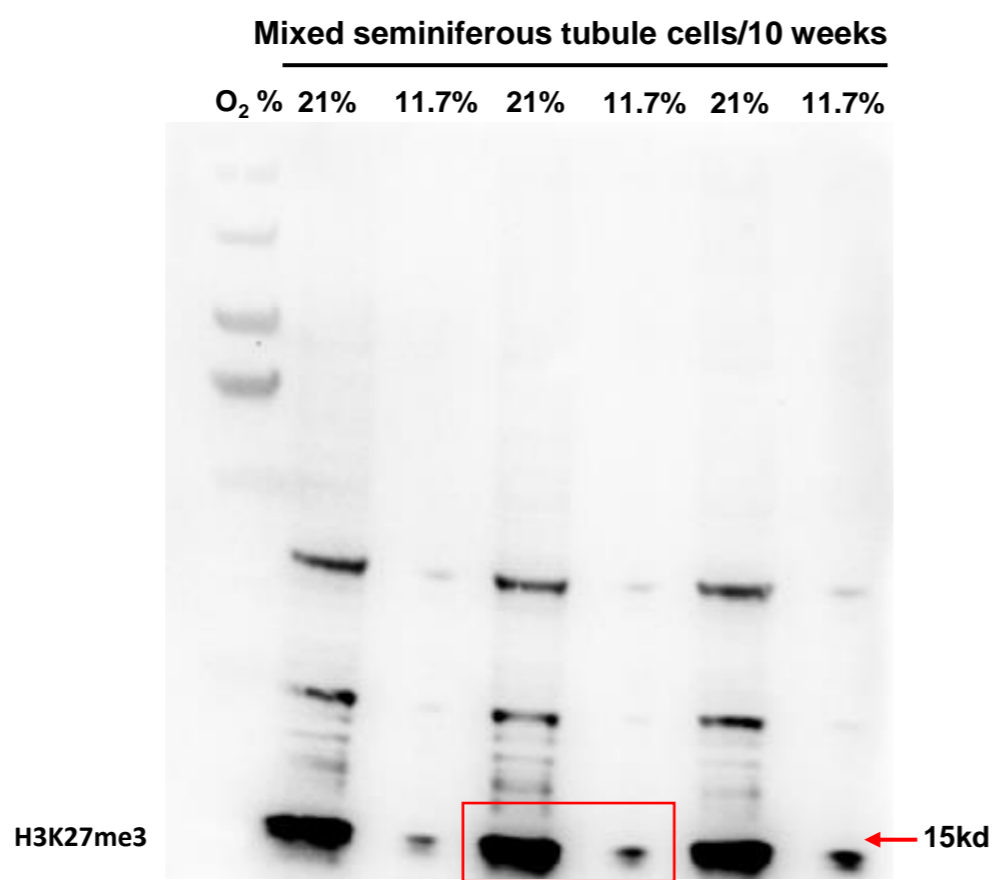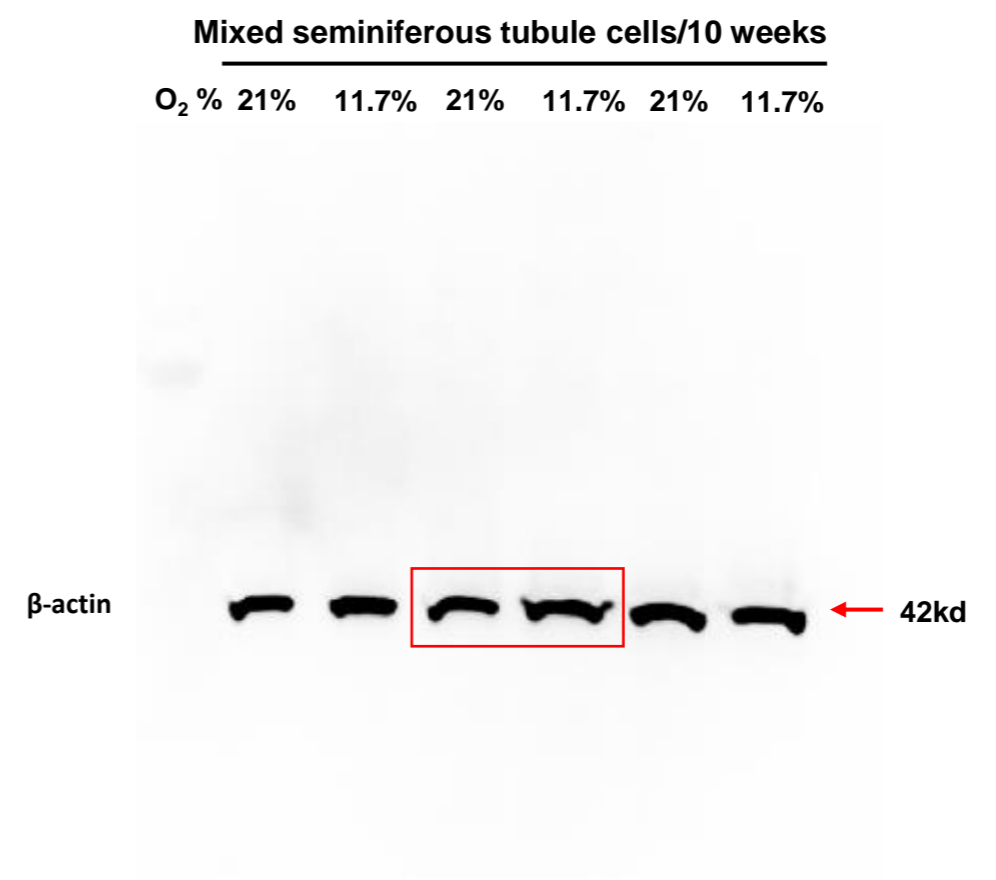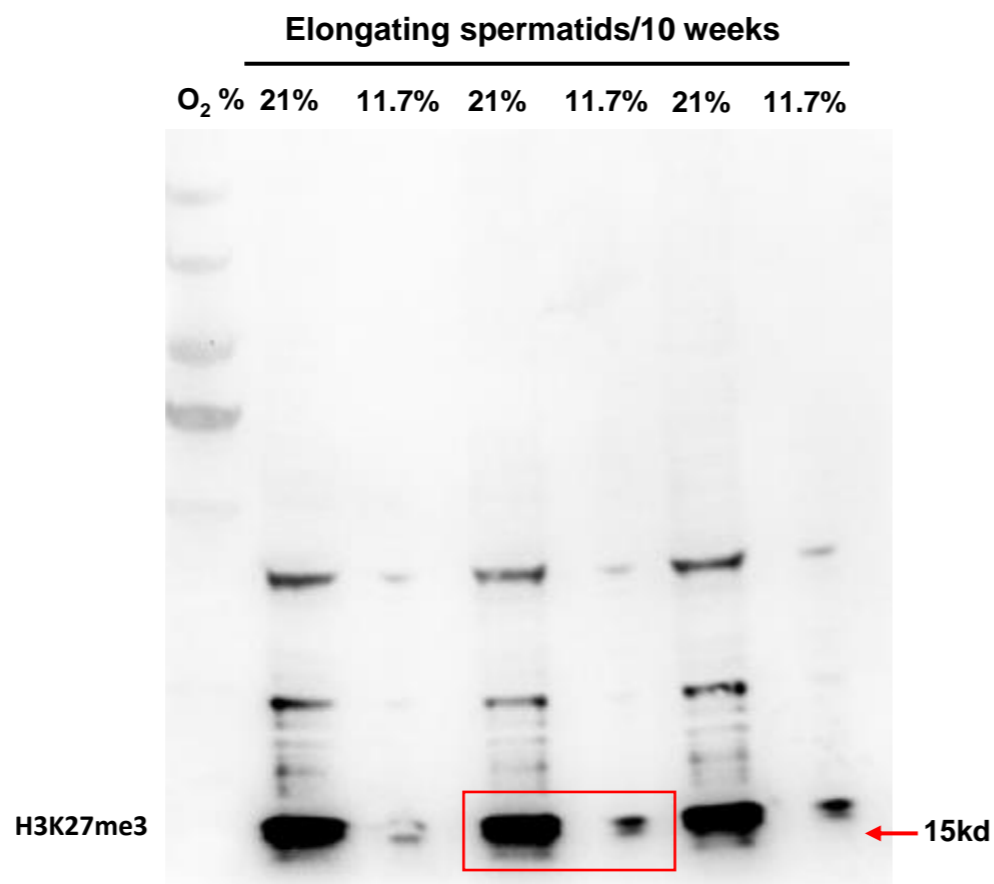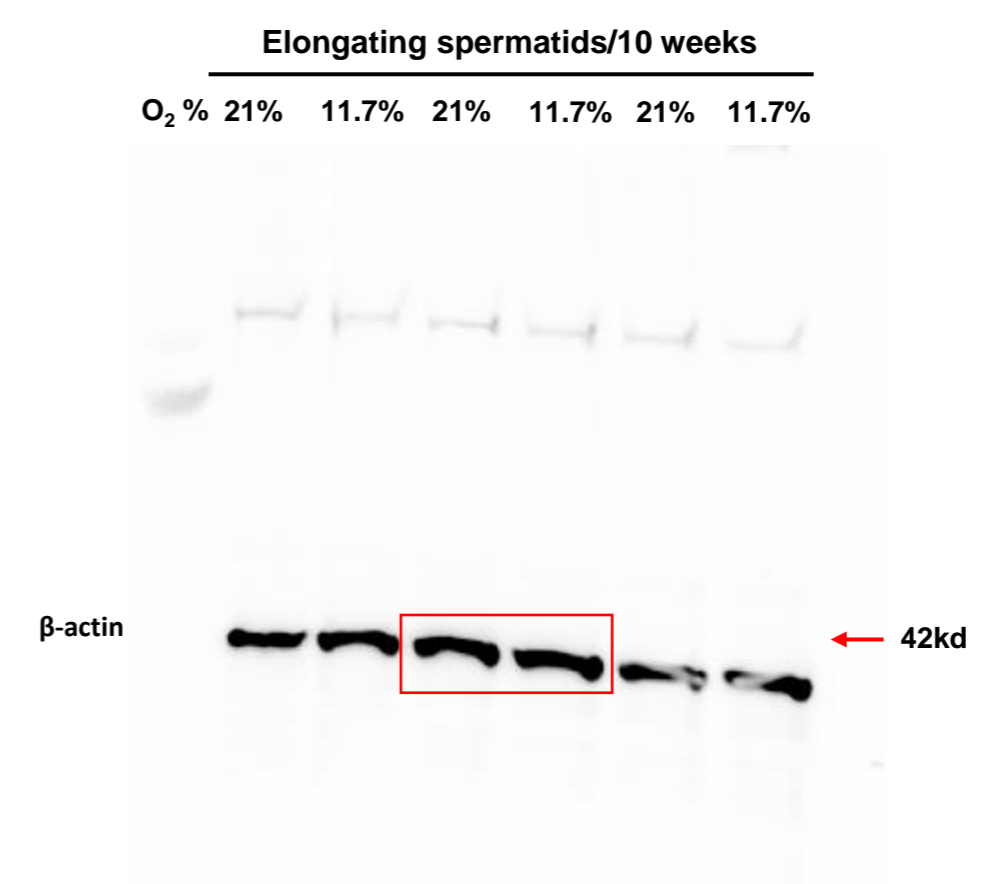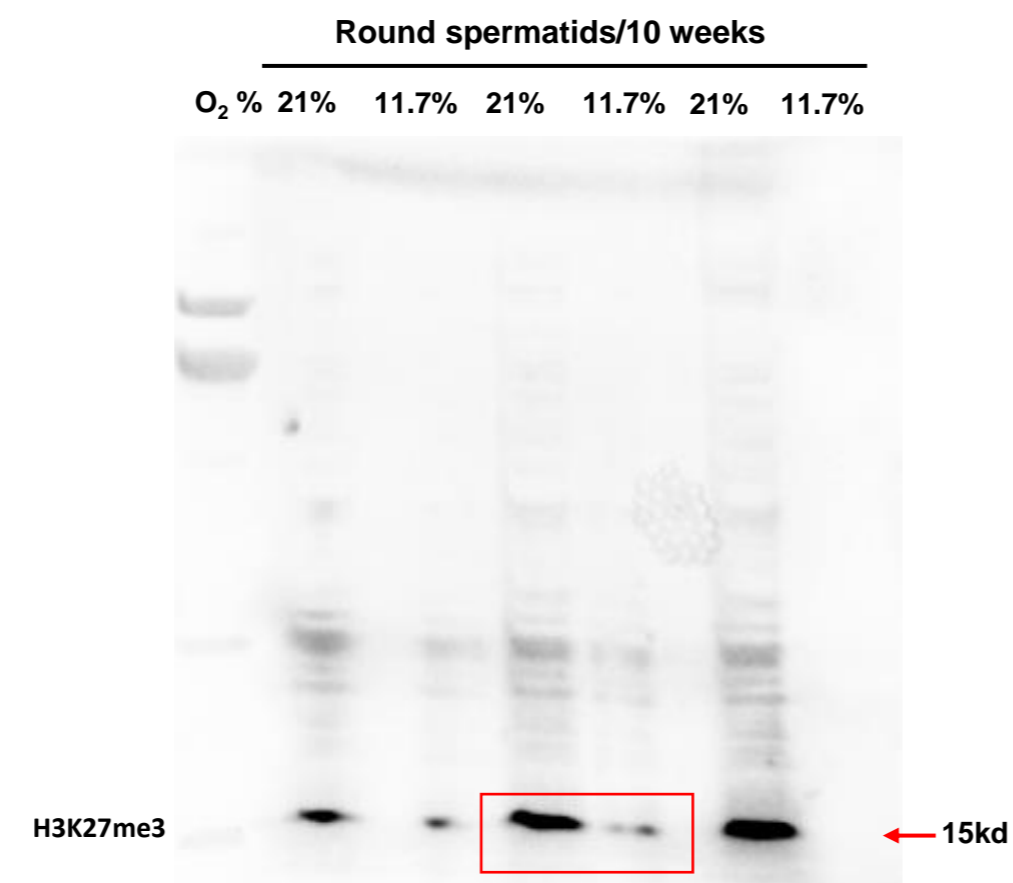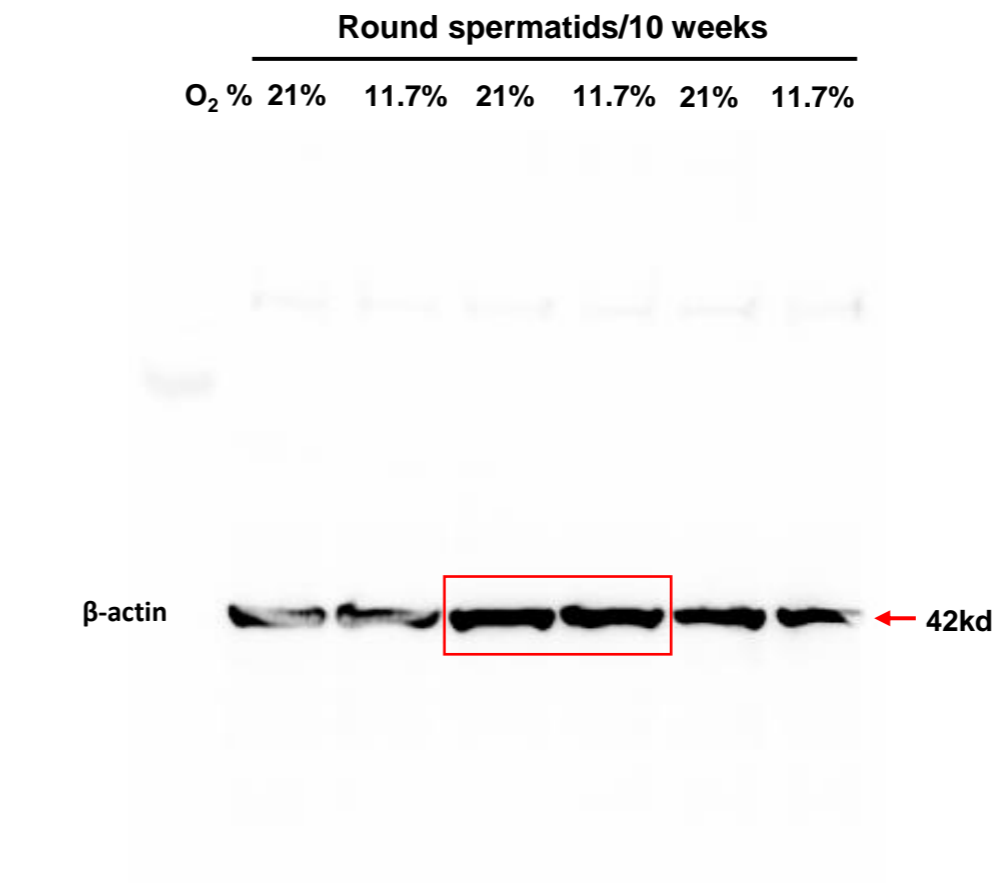

Figure 3

C

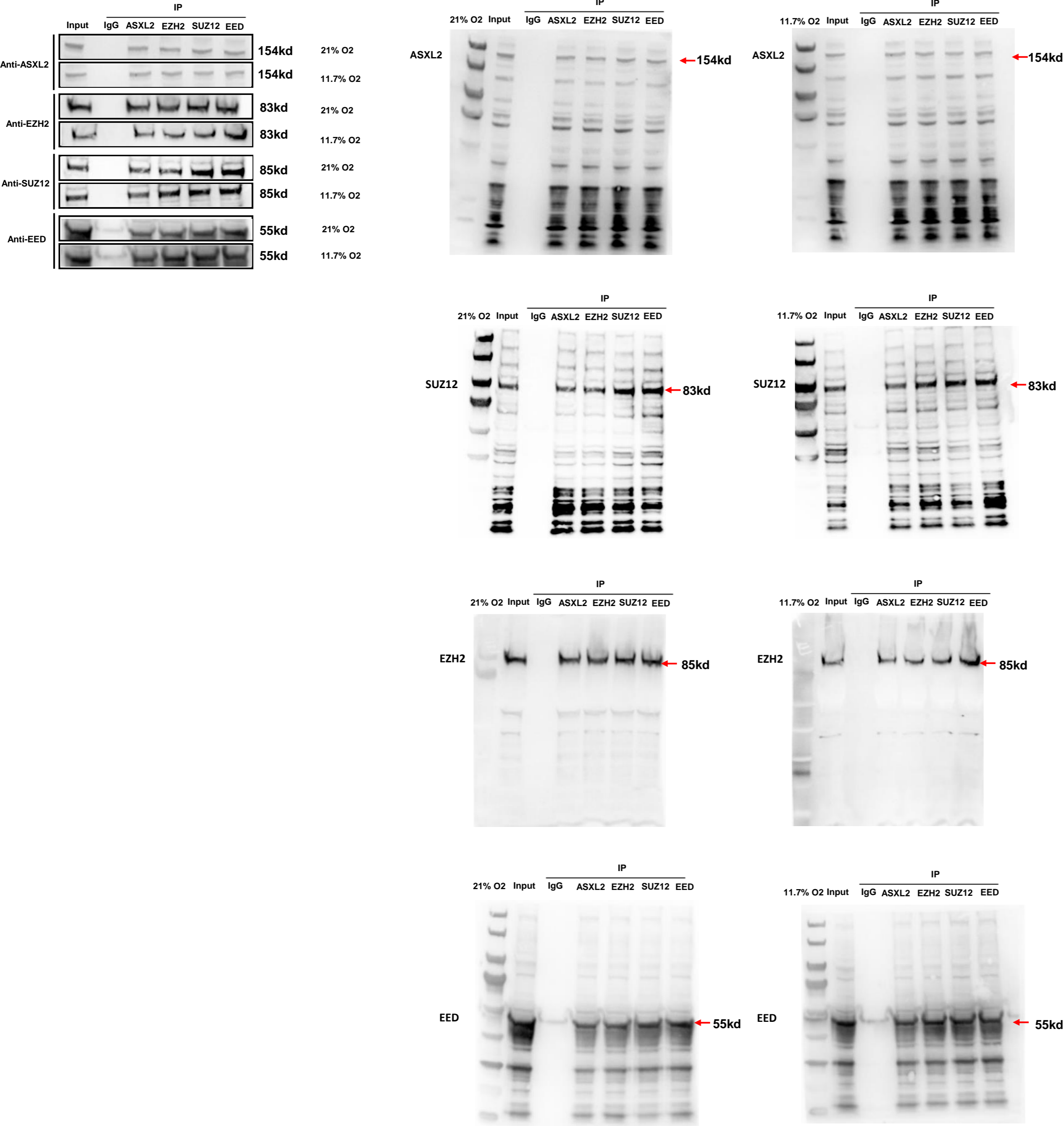

Figure 4

G

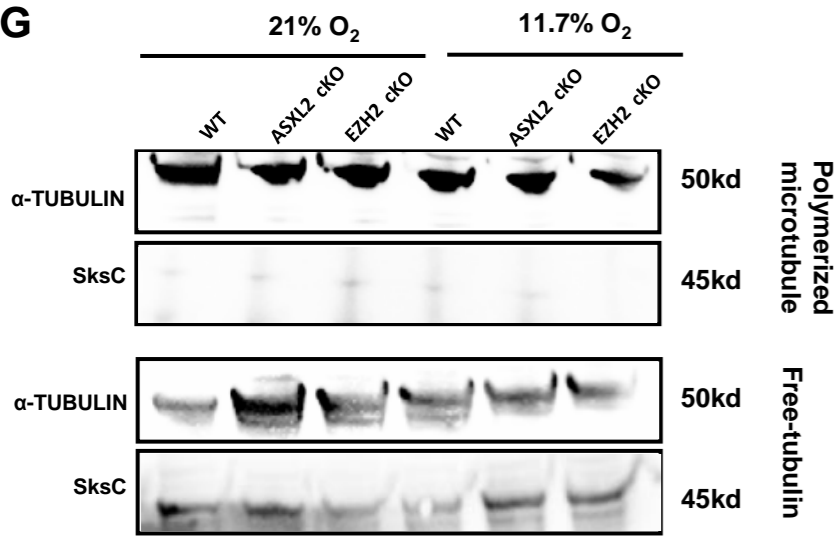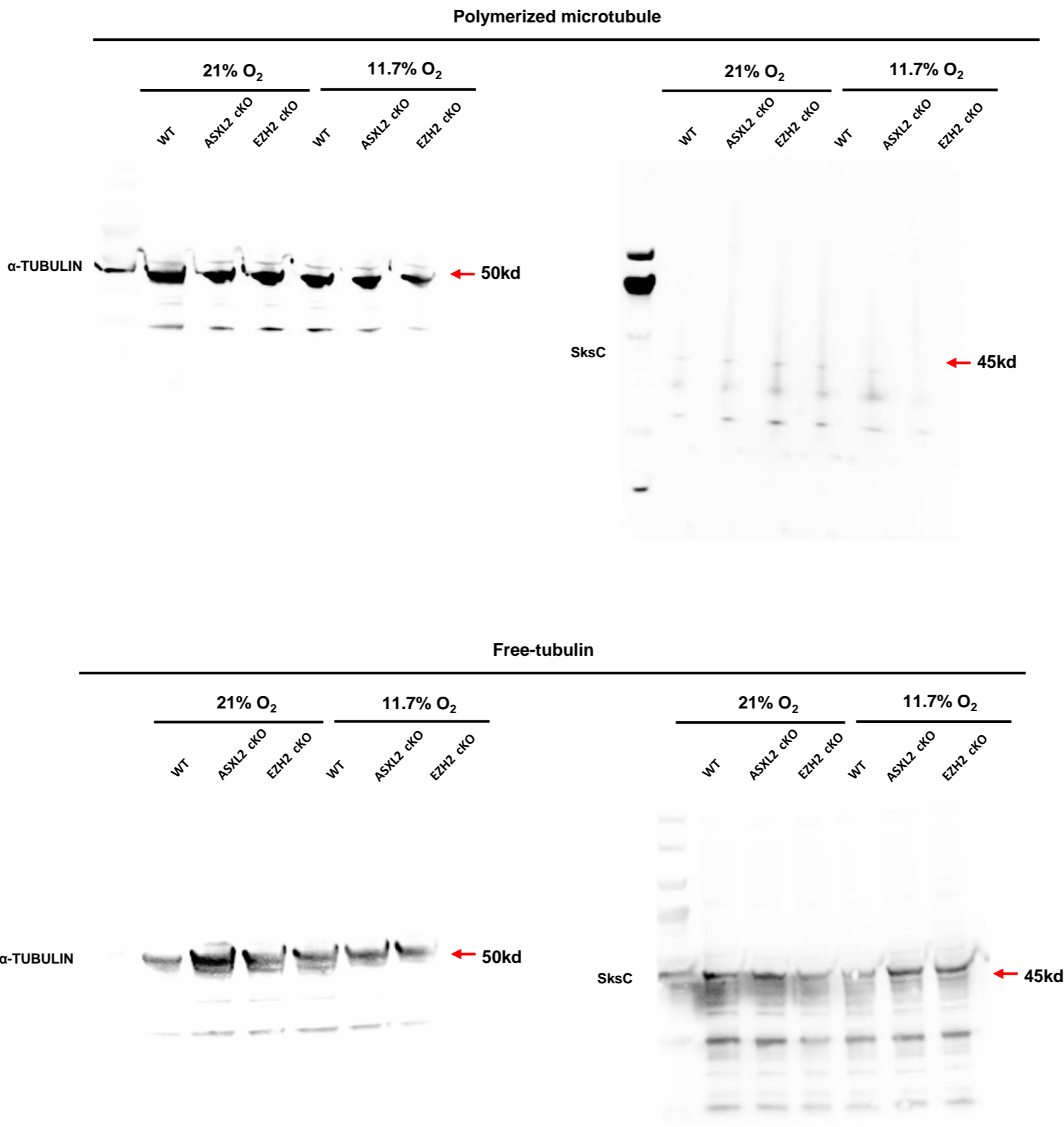

Figure 4

L

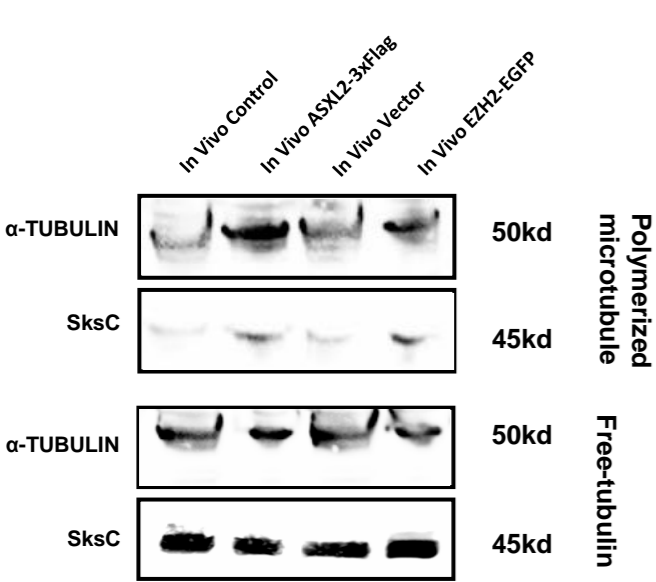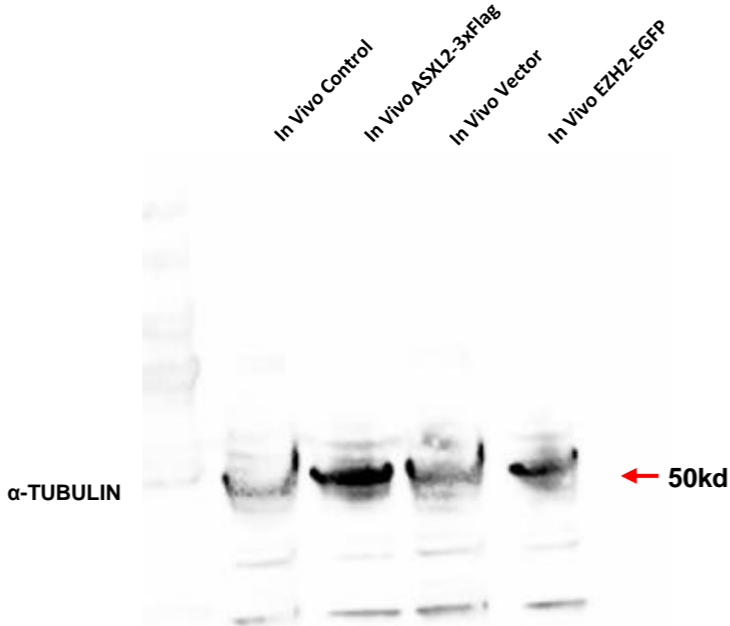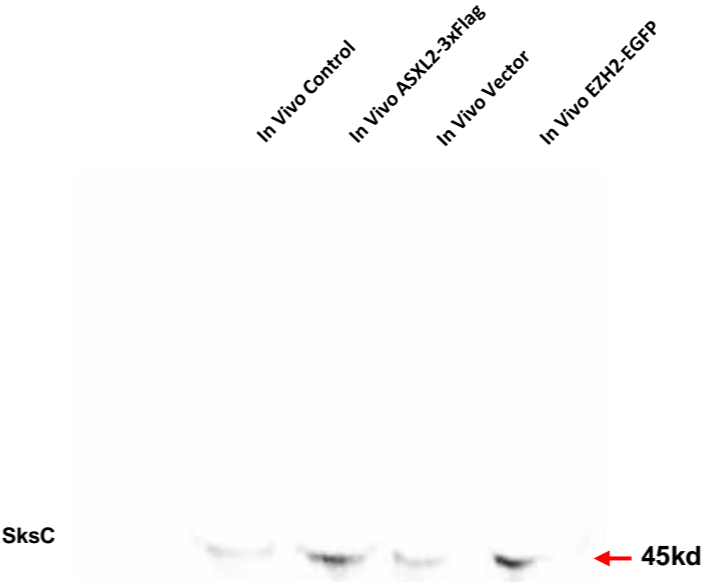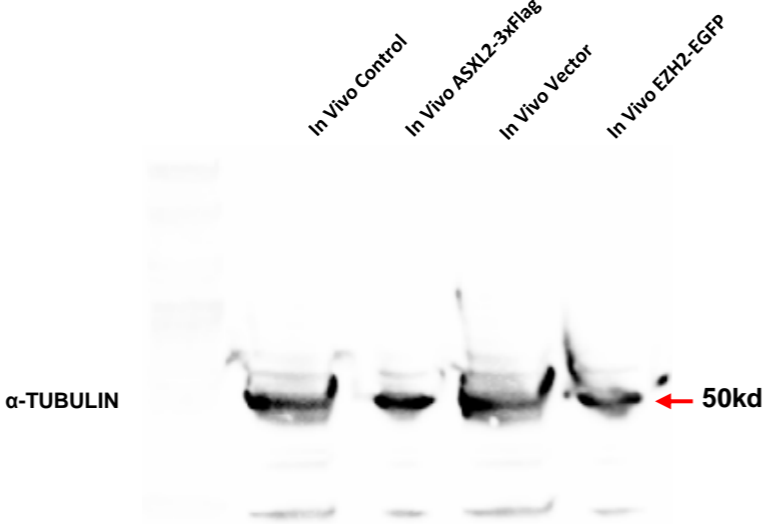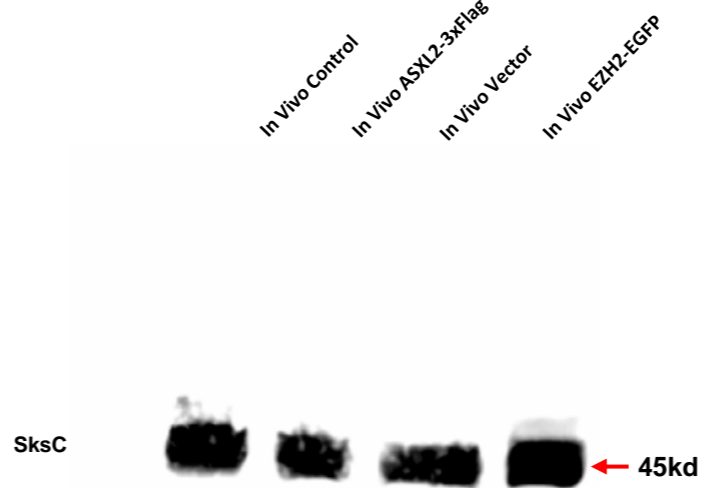

Figure 6

G

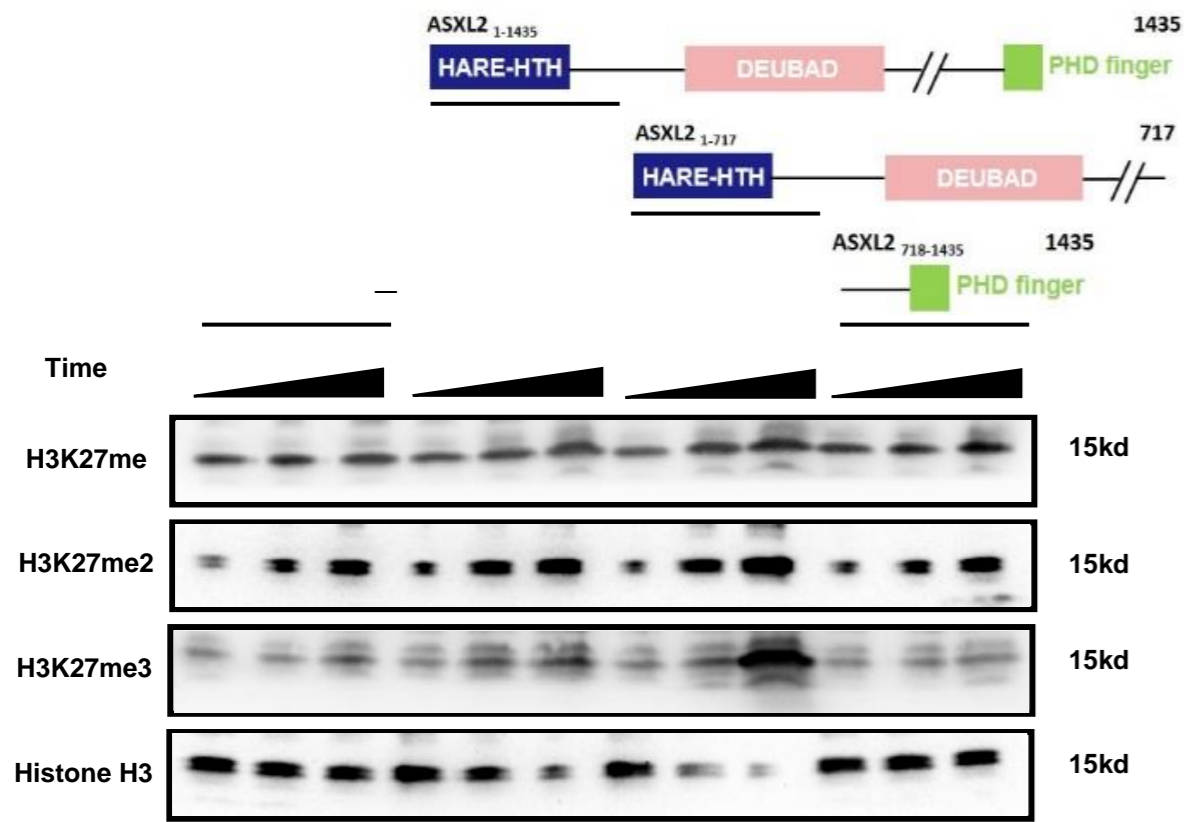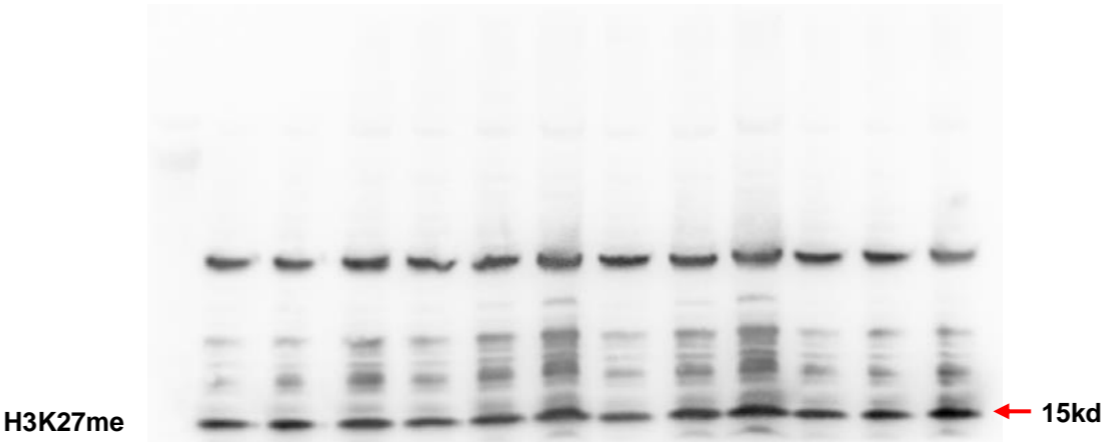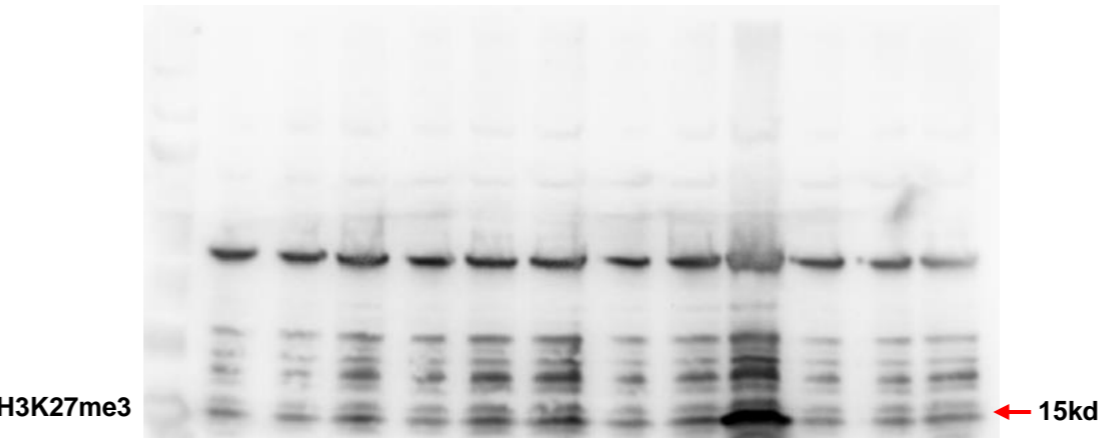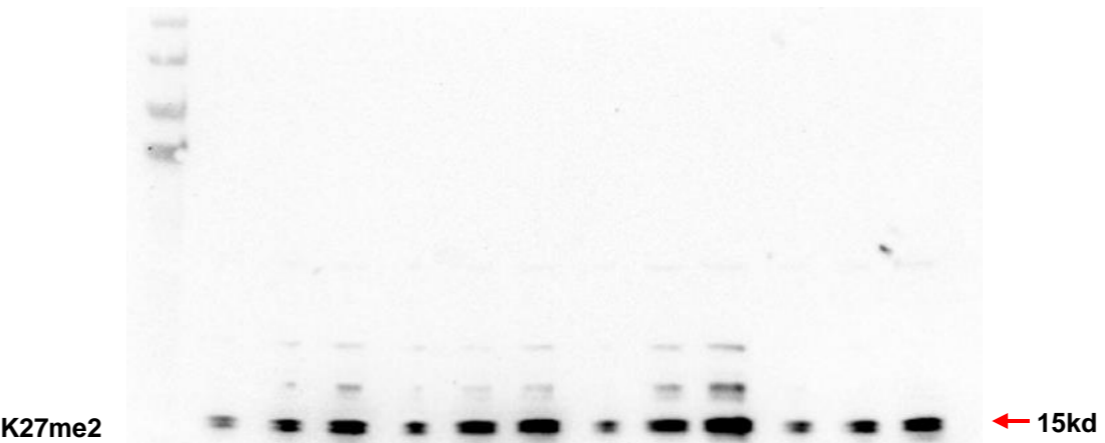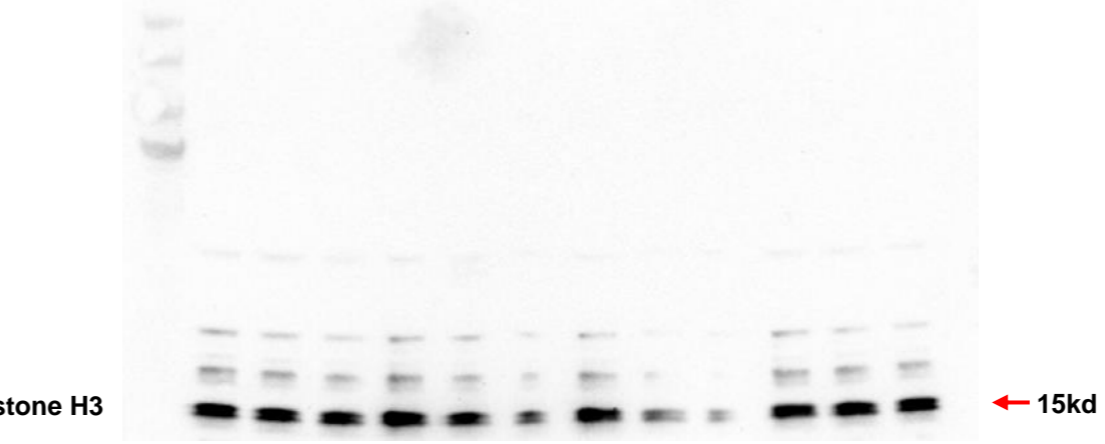

Figure 7b

I

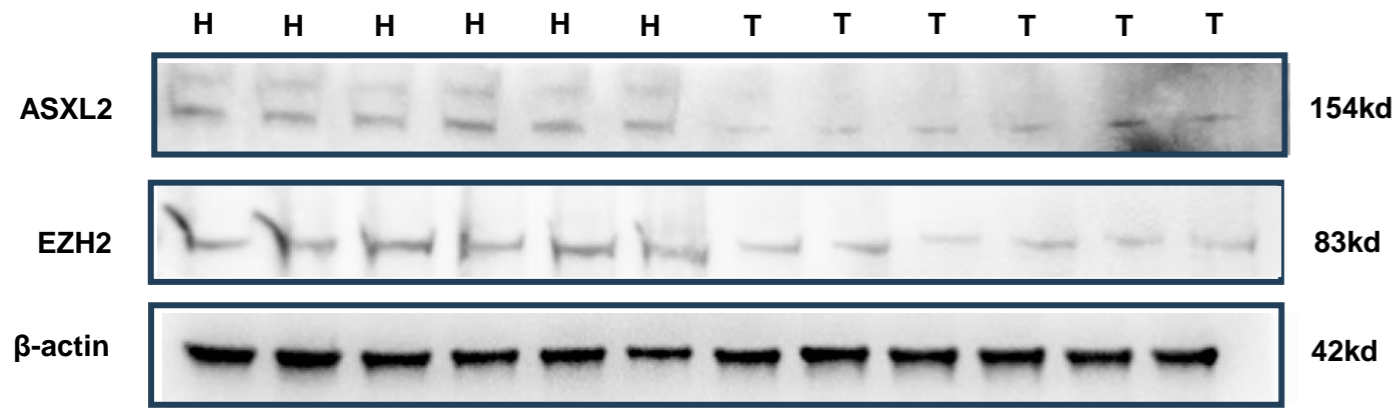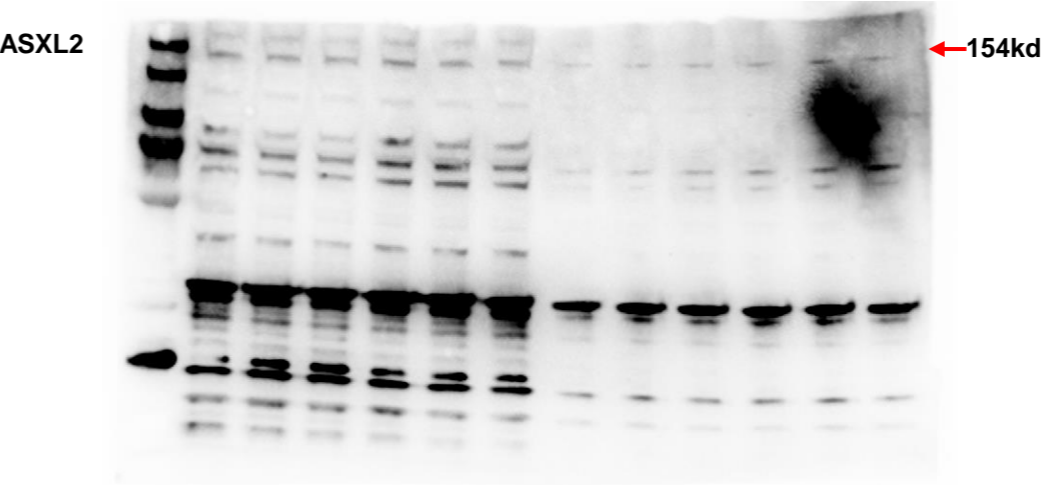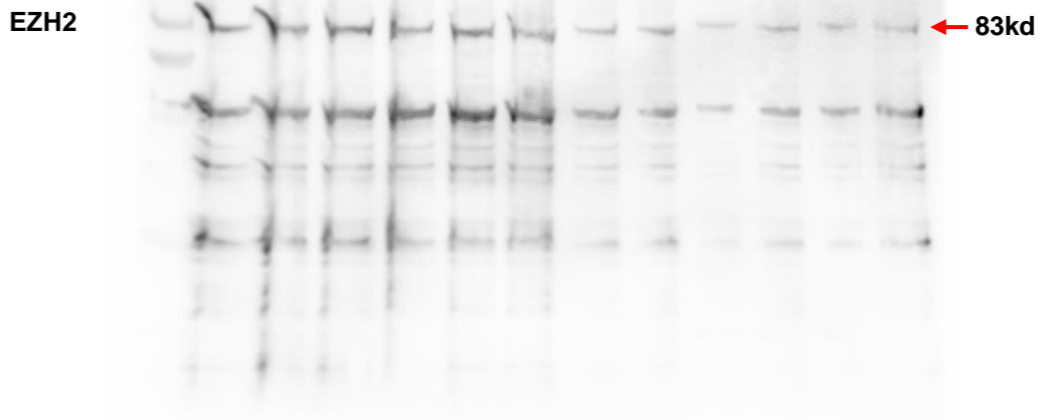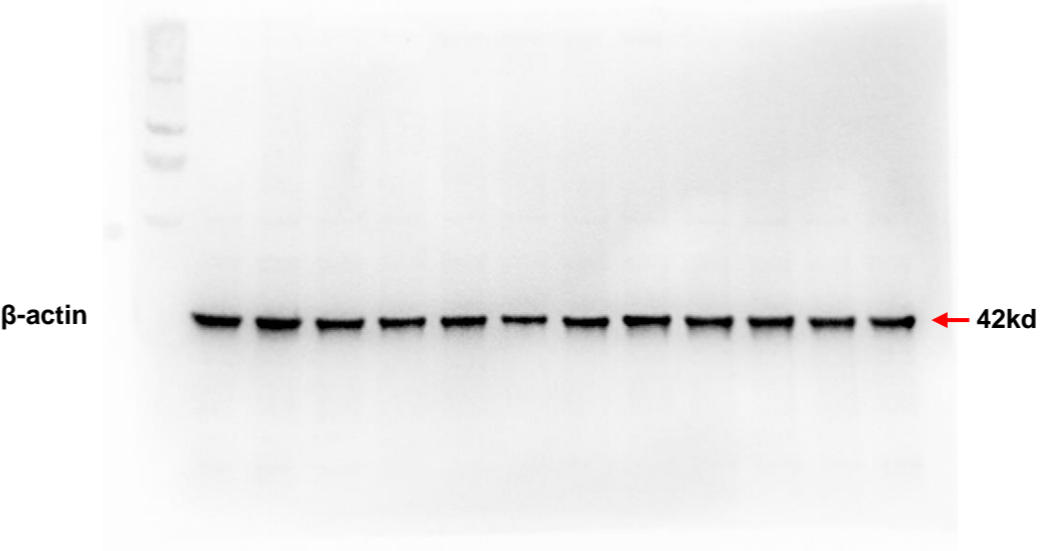

Figure S7

F

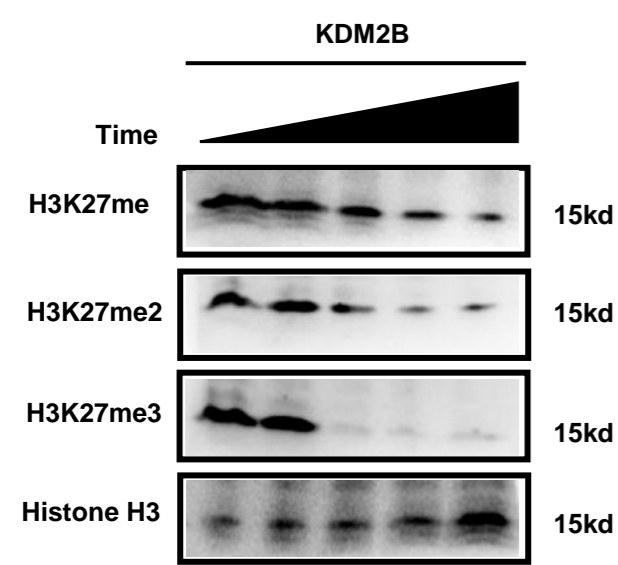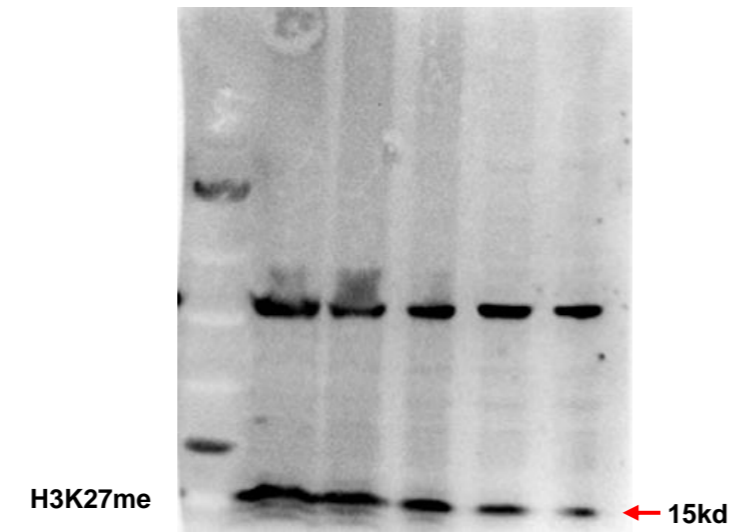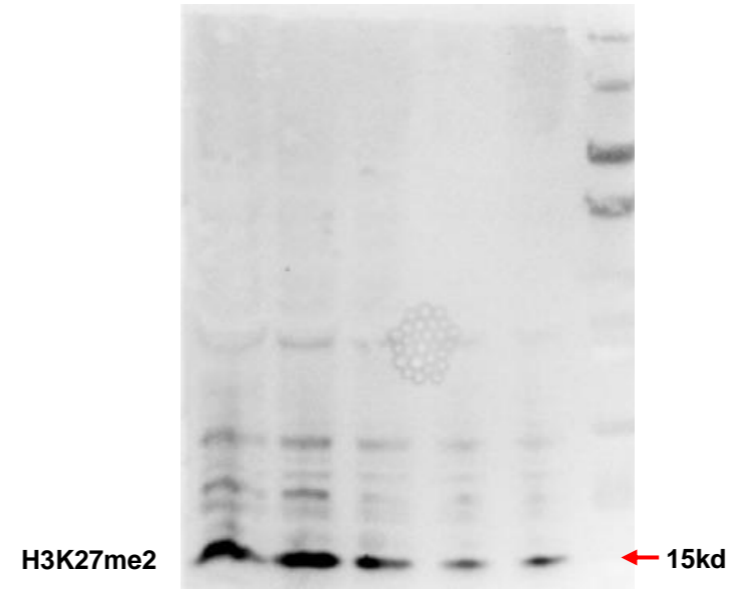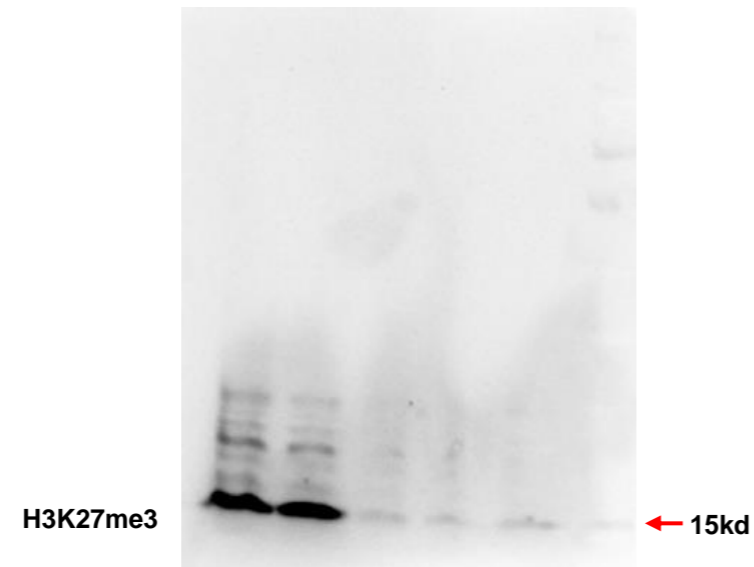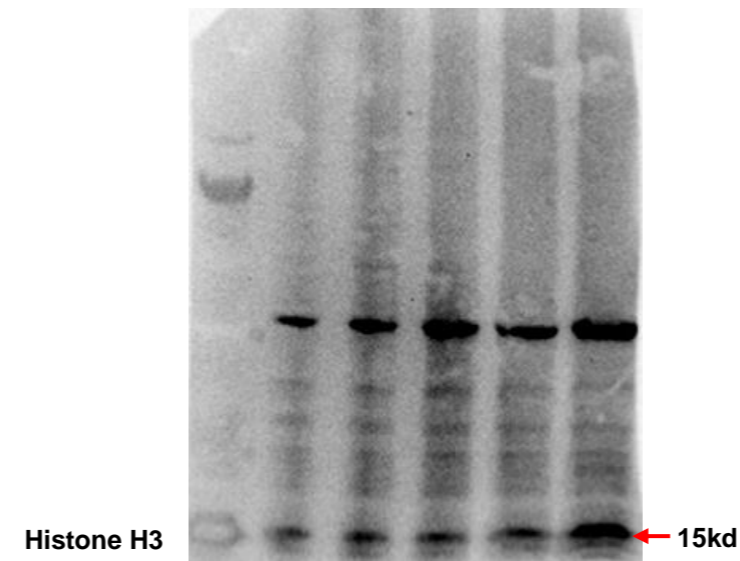

Supplement: Supplementary file 5 — Supporting File 5: advs74564‐sup‐0005‐Data.zip [file ADVS-13-e01266-s001.zip › advs74564-sup-0005-Data/Jun Yin_et_al_Supplementary_Data_FullMembrane_and_Omics/WB_Full_Membranes.pdf]
